# Molecular mechanisms underlying the extreme mechanical anisotropy of the flaviviral exoribonuclease-resistant RNAs (xrRNAs)

**DOI:** 10.1038/s41467-020-19260-4

**Published:** 2020-10-30

**Authors:** Xiaolin Niu, Qiuhan Liu, Zhonghe Xu, Zhifeng Chen, Linghui Xu, Lilei Xu, Jinghong Li, Xianyang Fang

**Affiliations:** 1 Beijing Advanced Innovation Center for Structfural Biology, School of Life Sciences, Tsinghua University, Beijing, 100084 China; 2 Key Laboratory of Bioorganic Phosphorus Chemistry and Chemical Biology, Department of Chemistry, Tsinghua University, Beijing, 100084 China

**Keywords:** Biophysical chemistry, RNA, Molecular biophysics, Single-molecule biophysics, Nanoscience and technology

## Abstract

Mechanical anisotropy is an essential property for many biomolecules to assume their structures, functions and applications, however, the mechanisms for their direction-dependent mechanical responses remain elusive. Herein, by using a single-molecule nanopore sensing technique, we explore the mechanisms of directional mechanical stability of the xrRNA1 RNA from ZIKA virus (ZIKV), which forms a complex ring-like architecture. We reveal extreme mechanical anisotropy in ZIKV xrRNA1 which highly depends on Mg^2+^ and the key tertiary interactions. The absence of Mg^2+^ and disruption of the key tertiary interactions strongly affect the structural integrity and attenuate mechanical anisotropy. The significance of ring structures in RNA mechanical anisotropy is further supported by steered molecular dynamics simulations in combination with force distribution analysis. We anticipate the ring structures can be used as key elements to build RNA-based nanostructures with controllable mechanical anisotropy for biomaterial and biomedical applications.

## Abstract (web summary)

The xrRNA1 RNA from ZIKA virus (ZIKV) forms a complex ring-like architecture and is known for its mechanical anisotropy, but the mechanism for its direction-dependent mechanical responses remain unclear. Here authors use a single-molecule nanopore sensing technique combined with molecular dynamics simulations and show that the anisotropy in ZIKV xrRNA1 depends on Mg^2+^ and the key tertiary interactions.

## Introduction

RNAs’ diverse roles in many cellular processes are dictated by their propensities to fold into stable three-dimensional structures driven by numerous tertiary interactions^1,2^. These tertiary interactions are important to RNAs’ structure, stability, dynamics, and folding kinetics^3^. Although RNA folds into stable structure as it is synthesized, it undergoes unfolding and refolding events during many cellular processes such as translation, replication, reverse transcription, etc., during which molecular motors exert forces on the RNA in a directional manner (i.e., ribosome proceeds along the RNA template in the 5′→3′ direction)^4^. For many nucleic acids, mechanical anisotropy is an inherent property which mechanical behavior varies with the direction of applied forces and is of high biological significance. For instance, the three-way-junction (3WJ)-pRNA derived from φ29 DNA packaging motor has been shown to exhibit high mechanical anisotropy upon Mg^2+^ binding, which relates to its capability to withstand the strain caused by DNA condensation^5,6^. The mechanical anisotropy of the human telomeric DNA G-quadruplex can be changed by ligand binding, corresponding to its regulation on replication or transcription^7^. Understanding how RNA responds to a mechanical stretching force and the molecular mechanisms defining its mechanical anisotropy is important for not only elucidating key principles governing various mechano-biological processes but developing novel RNA-based biomaterials with tailored mechanical properties and RNA-targeted therapeutics^8^, thus, has been an important research topic in the field of RNA mechanics^9^.

**Fig. 1:**
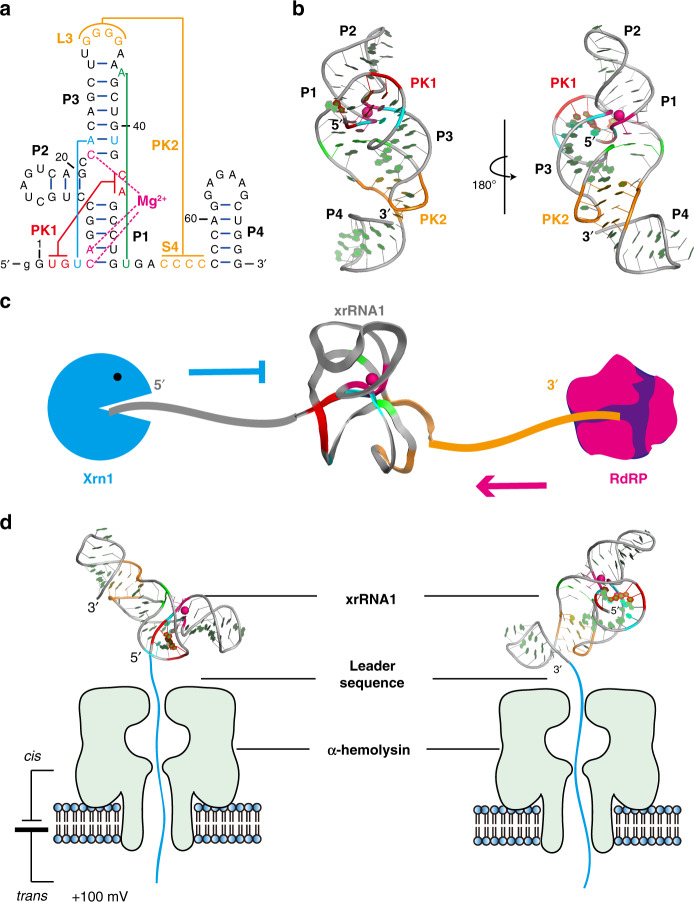
Mimicking directional unfolding of ZIKV xrRNA1 in the α-HL nanopore. **a**, **b** Secondary (**a**) and tertiary structure (**b**) of ZIKV xrRNA1, which contains several tertiary interactions (cyan: U4·A23:U41 base triple, green: A36:U50 reverse pair) and two pseudoknots, PK1 (red, U2G3:C43G44) and PK2 (orange, G30-G33:C53-C56). In addition, one Mg^2+^ is found to coordinate with the phosphate of C5, A6, and C22 (pink). **c** A cartoon shows that xrRNA1 can resist the degradation by exoribonuclease Xrn1 from 5′→3′ direction, yet be traversed by the viral RdRP from 3′→5′ direction. **d** Cartoons showing the directional unfolding and translocation of ZIKV xrRNA1 through the α-HL nanopore, where a 36 nt poly(rA) leader sequence was co-transcribed with xrRNA1 at the 5′- or 3′-end to facilitate its translocation through the nanopore.

Exoribonuclease-resistant RNAs (xrRNAs) are a group of RNA elements, which are capable of resisting the degradation by exonuclease^10,11^. The ability to resist Xrn1 is surprising, as Xrn1 is capable of processively degrading highly structured RNAs^12^. Recent crystal structures of xrRNAs from Murray Valley encephalitis virus (MVEV, xrRNA2)^13^ and ZIKA virus (ZIKV, xrRNA1)^14^ reveal a stable and compact RNA fold centered on a 3WJ that forms an unusual ring-like structure through which the 5′-end of the RNA is encircled protectively by a continuous stretch of RNA and the base pairs are “tucked away” behind backbone within the ring, forming a brace-like element that is unique to these structures (Fig. 1a, b). These structures suggest a “molecular brace” model for high resistance to directional degradation by the 5′–3′ exonucleases including Xrn1, Dxo1, and RNase J1, etc.^15,16^. Upon encountering the xrRNAs, Xrn1 must pull the 5′-end of the RNA through the ring, therefore creating a mechanical unfolding problem that the enzyme cannot resolve. In contrast, the viral RNA-dependent RNA polymerase (RdRP) approaching in the 3′→5′ direction during (−) strand synthesis can readily traverse this structure (Fig. 1c). It is hypothesized that the xrRNAs exhibit significant mechanical anisotropy^13,16^, which however has not been experimentally proved and the underlying mechanisms remain largely unknown.

Various single-molecule force spectroscopy techniques, e.g., optical tweezers, magnetic tweezers and atomic force microscope have been successfully applied to study RNA mechanical unfolding^7,8,17^. By pulling the RNAs from both ends, these approaches can identify the intermediate states along the folding pathway, but they also have obvious limitations in which the unfolding process is not unidirectional, different from that in vivo. Fortunately, recent developments in single-molecule nanopore-sensing technology have allowed the unfolding and translocation of RNAs through biological pores in a defined direction to be observed with fine details^18–20^. By taking advantage of the ability to electrically detect charged biomolecules through a nanometer-wide channel, several types of nanopores including α-hemolysin (α-HL) nanopore have been developed^21,22^. The narrowest constriction of α-HL is 1.4 nm in the stem domain (Supplementary Fig. S1) and, thus, it only allows the translocation of single-stranded nucleic acids, but folded nucleic acids must unfold to pass through the pore^21^.

In this work, using the α-HL nanopore-sensing technique, we investigate the directional mechanical stability of the xrRNA1 from ZIKV at single-molecule level. A 36 nt poly(rA) leader sequence is co-transcribed at the 5′- or 3′-end of ZIKV xrRNA1, respectively, to direct the translocation through the nanopore (Fig. 1d). The duration time that xrRNA1 is trapped in the nanocavity before unraveling provides a measure of the structure’s mechanical stability^19,20^. Extreme mechanical anisotropy is observed for ZIKV xrRNA1, which highly depends on the presence of Mg^2+^ and the tertiary interactions that stabilize the ring-like topological structure. The absence of Mg^2+^ and disruption of the key tertiary interactions strongly affect the structural integrity of the ring structure and attenuate mechanical anisotropy. The correlation is further supported by steered molecular dynamics (SMD) simulation combined with force distribution analysis (FDA) on ZIKV xrRNA1^23^, suggesting an important role of the ring-like architecture of ZIKV xrRNA1 in defining its mechanical anisotropy.

## Results

### ZIKV xrRNA1 exhibits extreme mechanical anisotropy

Mg^2+^ ions are known to be important for the structure and stability of RNA molecules^24^. As shown in Fig. 1a, b, a Mg^2+^ ion is found to coordinate with the phosphates of C5, A6, and C22 in the crystal structure of ZIKV xrRNA1, implying that Mg^2+^ ions may play an important role in the folding of xrRNA1. To better understand how Mg^2+^ affect the overall structure and stability of ZIKV xrRNA1, small-angle X-ray scattering (SAXS) and differential scanning calorimetry (DSC) experiments were performed (further details are provided in the Supplementary Information). SAXS data indicate a Mg^2+^-induced structural transition between the unfolded and folded states, and the midpoint of the transition is about 0.3 mM Mg^2+^ (Supplementary Fig. S2). DSC experiments demonstrated that the cooperative unfolding of xrRNA1 highly depends on Mg^2+^ concentrations (Supplementary Fig. S3). Both experiments show that xrRNA1 is fully folded in 5 mM Mg^2+^.

**Fig. 2:**
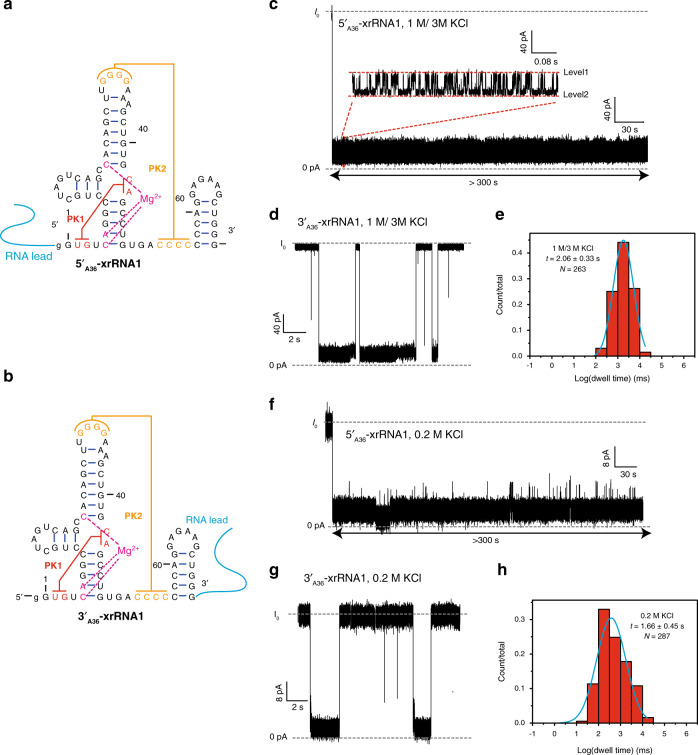
ZIKV xrRNA1 exhibits significant mechanical anisotropy in the presence of 5 mM Mg^2+^. **a**, **b** Secondary structure of 5′_A36_-xrRNA1 (**a**) and 3′_A36_-xrRNA1 (**b**), which a 36 nt poly(A) was co-transcribed at the 5′- or 3′-end of the xrRNA1. **c**, **d**, **f**, **g** Representative current blockade traces for unfolding of 5′_A36_-xrRNA1 in 1 M/3 M KCl (**c**) and 0.2 M KCl (**f**), 3′_A36_-xrRNA1 in 1 M/3 M KCl (**d**), and 0.2 M KCl (**g**) in the α-hemolysin nanopore. **e**, **h** Dwell time distribution histograms for the unfolding of 3′_A36_-xrRNA1 in 1 M (*cis*)/3 M (*trans*) KCl solutions (**e**) and 0.2 M KCl solution (**h**). The dwell time were presented as the mean ± SEM, which is calculated from *N* > 200 events and 3 independent repeat experiments.

To facilitate directional translocation of ZIKV xrRNA1 through the α-HL nanopore, a 36 nt poly(rA) leader sequence is co-transcribed with the xrRNA1 at the 5′- or 3′-end (hereafter as 5′_A36_-xrRNA1 and 3′_A36_-xrRNA1, respectively) (Fig. 2a, b). Similar designs have been used to facilitate directional translocation of the human telomere i-motif DNA and T2 pseudoknot RNA through α-HL nanopore^19,20,25^. DSC experiments on these xrRNA1s show that the leader sequence has minor effects on the thermal stability of xrRNA1s (Supplementary Fig. S4 and Supplementary Table S2). Under a transmembrane voltage, the single-stranded RNA leader sequence (36 nt, ~18 nm), which is much longer than the nanopore passage (10 nm), will be captured and occupy the entire nanopore channel first, then guides the directional unfolding and translocation of the downstream structured RNA elements through the nanopore and releases into the *trans* solution (Supplementary Fig. S5). While being captured and translocated through the confined space of the nanopore, the RNAs are subjected to significant electric forces and undergo structural changes then unfolding; the ions occupying the nanopore are excluded and therefore results in various characteristic current blockade signatures characterized by amplitude and duration time.

Initial nanopore-sensing studies were conducted for the ZIKV xrRNA1 sequences in 1 M (*cis*)/3 M (*trans*) KCl supplemented with 5 mM MgCl_2_ at +100 mV. Such an asymmetric solution design has been applied in other studies, which can enhance the capture rate of nucleic acid molecules^19^. Under such condition, the force applied to leader sequence is around 8 pN^19^, close to that exerted by molecular machines^26,27^. Figure 2c shows a representative current blockade signature for the 5′_A36_-xrRNA1 through the nanopore, for which only a very long current blockade is observed. The initial drop of the ionic current to <20% of the open-pore current (*I*_0_) is interpreted as entry of the 5′-RNA leader sequence into the pore and occupation of the β-barrel channel. This is further supported by nanopore-sensing experiments for xrRNA1s with different poly(rA) leader sequences in length, in which the initial drop of the ionic current becomes smaller as the leader sequences become longer (from 13% *I*_0_ to 10% *I*_0_ for 5′_A13_ and 5′_A15_), but does not change significantly across 5′_A20_, 5′_A30_, and 5′_A36_ (about 5% *I*_0_) (Supplementary Fig. S6). After the initial drop, the current switches frequently between two different levels with the relative conductance (*I*/*I*_0_) of 13% (Level 1) and 6% (Level 2), respectively. This may be due to the different orientations of xrRNA1 when threaded within the vestibule of the pore and similar phenomena were observed in the previous research^28^. After several attempts, we cannot observe any complete 5′_A36_-xrRNA1 unfolding and translocation event during 300 s time window, which is limited by vulnerability of biological nanopore system^29^. Further increasing the transmembrane voltage to 160 or 180 mV shows similar current blockade signatures (Supplementary Fig. S7). These initial observations suggest that the kinetics of 5′_A36_-xrRNA1 unfolding and translocation through the α-HL nanopore is too slow to be monitored in a practicable time window, preventing collecting enough single-molecule measurements for good statistics. In contrast, 3′_A36_-xrRNA1 in the same condition (5 mM Mg^2+^) is readily translocated through the α-HL nanopore, generating current blockade signatures distinct from that for 5′_A36_-xrRNA1 (Fig. 2d). Analysis of >200 of the events reveals four types of current blockade signatures, which are shown in Supplementary Fig. S8. Type I represents the most typical events and constitutes >85% of all the events. Type II is characterized with mean duration time <1 ms, which could be the translocation signals of single-stranded RNAs. Type III typically shows simple deep blockade current level (%*I*/*I*_0_ = 10%) and short duration time (~0.16 s), which should be the signals for partially folded xrRNA1. The remaining 6% forms type IV, which generates a two-stage blockade current pattern, similar to that produced by xrRNA1 without a leader sequence (Supplementary Fig. S9). Types II–IV do not feature the directional unfolding and translocation guided by the leader sequence. Therefore, type I signal is attributed to 3′→5′ directional translocation guided by the leader sequence and the following dwell time statistical analysis are all based on type I. The mean duration time for the 3′→5′ directional translocation of 3′_A36_-xrRNA1 in the nanopore is estimated as 2.06 ± 0.58 s (Fig. 2e), much shorter than that of 5′→3′ directional translocation of 5′_A36_-xrRNA1 in the nanopore, which is longer than 300 s. Due to the complexity of xrRNA1 structure and unfolding pathway^30^, it is difficult to have a thorough molecular interpretation of the blockade current traces; however, the duration time that xrRNA1 is trapped in the nanocavity before unraveling provides a measure for the structure’s mechanical stability^19,28,31^. The dramatic variation of the mean duration time of xrRNA1 between the 5′→3′ and 3′→5′ directional translocation in α-HL nanopore suggests that xrRNA1 exhibits extreme mechanical anisotropy.

To investigate whether ZIKV xrRNA1 exhibits mechanical anisotropy in low salt concentrations, we also performed nanopore-sensing experiments for 5′_A36_-xrRNA1 and 3′_A36_-xrRNA1 in near-physiological conditions (0.2 M KCl, 5 mM Mg^2+^)^19^. As no signals for the entry of leaders into the pore can be observed at +100 mV, the experiments were carried out at +180 mV instead. The current blockade signals for both RNA constructs were similar to that in 1 M/3 M KCl (Fig. 2f, g). Although about 20% of 5′_A36_-xrRNA1 translocate through the nanopore channel with duration time longer than 100 s, the remaining 80% are still not observed to translocate through the pore even in a 300 s time window. In contrast, the mean duration time for 3′_A36_-xrRNA1 in 0.2 M KCl is 1.66 ± 0.45 s, slightly shorter than that in 1 M/3 M KCl (Fig. 2h). Thus, the duration time for 5′_A36_-xrRNA1 remains much longer than that for 3′_A36_-xrRNA1 and the extreme mechanical anisotropy of xrRNA1 preserved even at 0.2 M KCl. However, due to the poor signal-to-noise ratio and lower translocation frequency in low salt concentration, the following nanopore-sensing experiments were all conducted in the asymmetric solution of 1 M (*cis*)/3 M (*trans*) KCl at +100 mV.

### The mechanical anisotropy of ZIKV xrRNA1 highly depends on Mg^2+^

The prolonged duration time (>300 s) for 5′_A36_-xrRNA1 in 5 mM Mg^2+^ prevents obtaining required populations of current blockages for statistical analysis; thus, mutants of xrRNA1 were designed and screened to reduce its 5′→3′ mechanical stability mildly but maintain overall structure, which results in a quadruple mutant (U2A + U28A + A36C + C53G, hereafter as xrRNA1-X), which resembles ZIKV xrRNA1 (Supplementary Fig. S10). In the crystal structure of ZIKV xrRNA1, U2 (U2-A44) and C53 (C53-G33) are involved in long-range base-pairing interactions in the first (PK1) and second (PK2) pseudoknots formation, respectively; A36 and U50 form a reverse Watson–Crick long-range base pair that closes the ring structure to “lasso” the RNA that passes through; U28 and A35 form a Hoogsteen base pair, which is adjacent to PK2 (Fig. 1a, b). DSC experiments show that the quadruple mutation cause marginal reduction in the thermal stability of ZIKV xrRNA1; the melting temperature for xrRNA1-X is only slightly lower than ZIKV xrRNA1 (from 75.2 to 78.52 °C) (Supplementary Fig. S10c). SAXS analysis shows that xrRNA1-X shares similar pair distance distribution function (PDDF) (*R*_*g*_, *D*_max_), as xrRNA1 and the experimental scattering curve of xrRNA1-X in 5 mM Mg^2+^ can fit nicely with ZIKV xrRNA1 crystal structure (Supplementary Figs. S10d and 11a). These data clearly show that the quadruple mutations in xrRNA1-X marginally reduce xrRNA1’s overall stability and cause minimal effects on its overall structure.

**Fig. 3:**
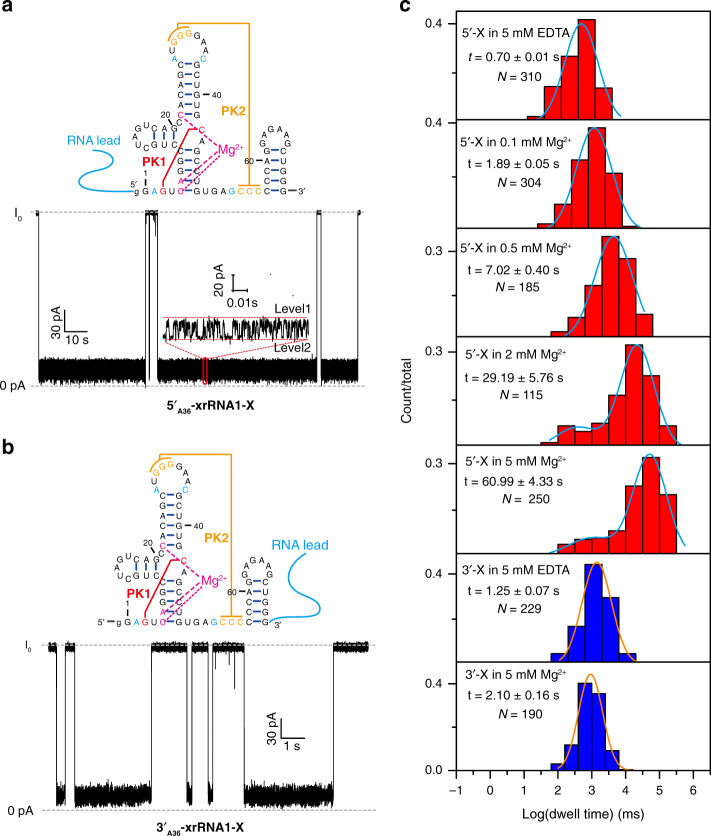
Mechanical anisotropy of ZIKV xrRNA1-X highly depends on Mg^2+^. **a**, **b** Secondary structures and representative current traces for the unfolding and translocation of 5′_A36_-xrRNA1-X (**a**) and 3′_A36_-xrRNA1-X (**b**) through the nanopore in the presence of 5 mM Mg^2+^. **c** Dwell time distribution histograms for 5′_A36_-xrRNA1-X (red) and 3′_A36_-xrRNA1-X (blue) in different Mg^2+^ concentrations.

Both 5′_A36_-xrRNA1-X and 3′_A36_-xrRNA1-X were analyzed by nanopore-sensing studies. As expected, 5′_A36-_xrRNA1-X in 5 mM Mg^2+^ readily undergoes 5′→3′ directional translocation through the α-HL nanopore at +100 mV within a measurable time window. 5′_A36_-xrRNA1-X shares some similarity in blockade current signatures as 5′_A36_-xrRNA1, e.g., the leader sequence of 5′_A36_-xrRNA1-X is first captured by the nanopore and occupies the β-barrel channel; therefore, this causes rapid drop of the current to <20% of the open channel current and the current switches frequently between two different levels with the relative conductance (*I*/*I*_0_) of 13% (Level 1) and 6% (Level 2), respectively (Fig. 3a). The mean duration time of 5′_A36_-xrRNA1-X through the nanopore is estimated to be 60.78 ± 5.32 s (Fig. 3c). The blockade current signature for 3′_A36_-xrRNA1-X is similar to that in 5 mM Mg^2+^ (Fig. 3b) and the mean duration time is estimated to be 2.00 ± 0.15 s, which is about 30 times shorter than that for 5′_A36_-xrRNA1-X (Fig. 3c). Obviously, similar to xrRNA1, xrRNA1-X exhibits significant mechanical anisotropy in 5 mM Mg^2+^; thus, xrRNA1-X could be used to represent xrRNA1 in the subsequent quantitative analysis. It should be noted that the disruption of some tertiary interactions by the quadruple mutations in xrRNA1-X may cause some undetected differences in folding kinetics or intermediates as compared with xrRNA1, although their stability and overall structures are very similar.

It is interesting to know how Mg^2+^ affects the directional mechanical stability of xrRNA1^19^. To conduct the Mg^2+^-dependent directional translocation studies, 5′_A36_-xrRNA1-X was placed in electrolyte solution containing 1 M (*cis*)/3 M (*trans*) KCl supplemented with various concentrations of Mg^2+^. The blockade current signature for 5′_A36_-xrRNA1-X in different Mg^2+^ concentrations are very similar (Supplementary Fig. S12) but the mean duration time becomes longer as Mg^2+^ concentration increases. xrRNA1 shows a well-defined distribution centered at ~0.7 s in 5 mM EDTA, as Mg^2+^ increases a second peak emerged and shifts to longer dwell times above 0.5 mM Mg^2+^, which is consistent with Mg^2+^-induced folding of xrRNA1 derived from SAXS and DSC data (Fig. 3c). These data indicated that the 5′→3′ directional mechanical stability of xrRNA1 highly depends on Mg^2+^. In contrast, the 3′→5′ directional mechanical stability of xrRNA1 is less dependent on Mg^2+^. The mean duration time for 3′_A36_-xrRNA1-X in 5 mM EDTA is estimated to be 1.21 ± 0.1 s, only slightly smaller than that in 5 mM Mg^2+^ (2.00 ± 0.15 s), and the blockade current signatures are very similar (Fig. 3c). Overall, the mechanical anisotropy of ZIKV xrRNA1, which is the difference between the 5′→3′ and 3′→5′ directional mechanical stability, highly depends on Mg^2+^.

### Loss of key tertiary interactions attenuates ZIKV xrRNA1 mechanical anisotropy

As shown in Fig. 1a, b, ZIKV xrRNA1 contains a ring-like architecture centered on a 3WJ, which is further stabilized by two pseudoknots^14^. The helical element in PK2 can form a continuous helix with the P4 duplex through coaxial stacking, which may further stabilize the overall fold^14^. To understand how these tertiary interactions affect the mechanical anisotropy, mutants of ZIKV xrRNA1 were generated and analyzed by nanopore sensing.

**Fig. 4:**
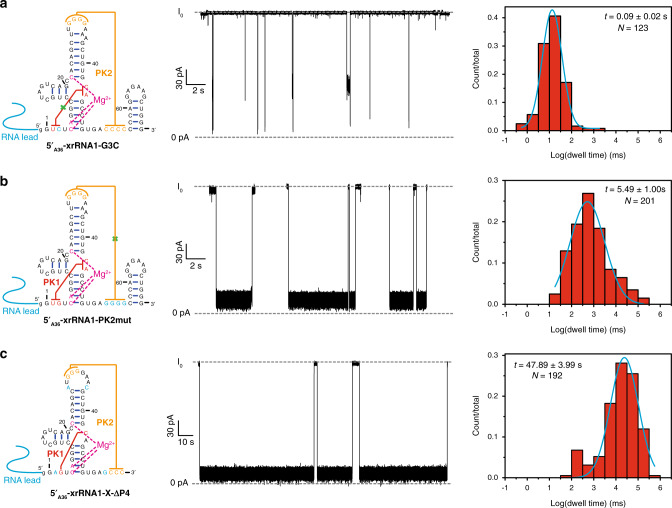
Contributions of key tertiary interactions to the directional mechanical stability of ZIKV xrRNA1. **a**–**c** Secondary structures (left), representative current blockade traces (middle), and the respective dwell time histogram (right) for the directional mechanical unfolding of 5′_A36_-xrRNA1-G3C (**a**), 5′_A36_-xrRNA1-PK2mut (**b**), and 5′_A36_-xrRNA1-X-∆P4(**c**) in the α-HL nanopore.

Previous studies on MVEV xrRNA2 suggested the importance of S1–S3 pseudoknot in stabilizing the active conformation of xrRNA2^13^. Disruption of the pseudoknot by mutation of either base (G3C and C40G) abolished the ability of the MVEV RNA to resist Xrn1 degradation^13^. Similarly, G3C mutant of ZIKV xrRNA1 also abolishes its Xrn1 resistance ability in our research (Supplementary Fig. S10b). SAXS data indicated that G3C mutant has larger *R*_*g*_ and *D*_max_, and Kratky plot with feature for partially folded molecules even in the presence of 5 mM Mg^2+^, as compared with ZIKV xrRNA1 (Supplementary Fig. S11 and Supplementary Table S3). These data suggest that G3C mutation could prevent PK1 formation and proper folding of xrRNA1 which results in disrupted ring structure. To study the significance of PK1 in ZIKV xrRNA1 mechanical anisotropy, we constructed both 5′_A36_-xrRNA1-G3C and 3′_A36_-xrRNA1-G3C, which were used in nanopore sensing. For 5′_A36_-xrRNA1-G3C, the blockade current pattern was distinct from that of 5′_A36_-xrRNA1 and the relative conductance was kept at a deep current state until RNA pass through the nanopore channel. As expected, the average duration time for 5′_A36_-xrRNA1-G3C (0.09 ± 0.04 s) decreased significantly with a reduction ratio of more than 1667 and 675 as compared with 5′_A36_-xrRNA1 (longer than 300 s) and 5′_A36_-xrRNA1-X (60.78 ± 5.32 s), respectively (Fig. 4a). These data suggest that the PK1 pseudoknot contributes greatly to the 5′→3′ directional mechanical stability of xrRNA1. Nonetheless, both the blockade pattern and duration time of 3′_A36_-xrRNA1-G3C in the nanopore were similar to that of 3′_A36_-xrRNA1 (Supplementary Fig. S13a); therefore, PK1 pseudoknot interaction has minor effect on the 3′→5′ directional mechanical stability of xrRNA1 and thus play an important role in the mechanical anisotropy of xrRNA1.

The PK2 pseudoknot formed by base pairing between L3 and S4 is also crucial to the tertiary structure by latching the ring-like architecture; mutations that disrupt PK2 for which 4Cs in S4 were replaced with 4Gs (xrRNA1-PK2mut) have been found to seriously weaken the resistance ability of ZIKV xrRNA1^14^. SAXS data indicate that xrRNA1-PK2mut has larger *R*_*g*_ and *D*_max_ as compared with ZIKV xrRNA1, and its Kratky plot has feature of partially folded molecule. The experimental scattering curve of xrRNA1-PK2mut fits poorly to ZIKV xrRNA1 crystal structure (Supplementary Fig. S11a). All these suggest the disruption of the ring structures. Similarly, 5′_A36_-xrRNA1-PK2mut and 3′_A36_-xrRNA1-PK2mut constructs were made to investigate the importance of the PK2 on the directional mechanical stability of ZIKV xrRNA1. Although both constructs share similarities in blockade current signature as that of 5′_A36_-xrRNA1-X and 3′_A36_-xrRNA1-X, in which the current switches between two levels frequently (Supplementary Figs. S13b and S14), the elimination of the PK2 pseudoknot result in a shorter duration time (5.49 ± 1.74 s) for 5′_A36_-xrRNA1-PK2mut compared to 5′_A36_-xrRNA1 (>300 s) with a reduction ratio of 53.6 (Fig. 4b), but similar duration time (4.05 ± 0.54 s) for 3′_A36_-xrRNA1-PK2mut compared to 3′_A36_-xrRNA1 (2.06 ± 0.58 s). Hence, PK2 tertiary interaction also contributes significantly to the 5′→3′ but has marginal effects on the 3′→5′ directional mechanical stability of ZIKV xrRNA1, in accordance with our Xrn1 resistance assay that PK2mut retains a weak resistance capability to Xrn1 while G3C totally can’t (Supplementary Fig. S11e).

To understand whether the coaxial stacking between the PK2 helix and P4 duplex contributes to the mechanical stability of ZIKV xrRNA1, we constructed P4 deletion mutant in the context of xrRNA1-X, which was referred as xrRNA1-X-ΔP4. The blockade current patterns for both 5′_A36_-xrRNA1-X-ΔP4 (Fig. 4c) and 3′_A36_-xrRNA1-X-ΔP4 (Supplementary Fig. S13c) are similar to that of 5′_A36_-xrRNA1-X and 3′_A36_-xrRNA1-X, respectively. However, deletion of P4 slightly shortens the duration time of 5′_A36_-xrRNA1-X as compared with 5′_A36_-xrRNA1-X in the nanopore (46.77 ± 8.81 s vs. 60.78 ± 5.32 s) (Fig. 4c). Thus, the coaxial stacking interaction between PK2 helix and P4 duplex contributes modestly to the 5′→3′ directional mechanical stability of xrRNA1. This result is consistent with our Xrn1 resistance assay that truncation of P4 marginally impairs ZIKV xrRNA1’s ability to resist Xrn1 exoribonuclease, although the biological function of P4 is unknown (Supplementary Fig. S11e). SAXS data analysis of xrRNA1-X-ΔP4 and xrRNA1-ΔP4 shows that the experimental scattering profiles can nicely fit with the crystal structure of ZIKV xrRNA1 without P4, indicating that the truncation of P4 has little effect on the structural integrity of the ring-like core architecture of xrRNA1 (Supplementary Fig. S11a). The effect of truncation of P4 on the 3′→5′ directional mechanical stability was also probed. However, the duration time for 3′_A36_-xrRNA1-X-ΔP4 (4.2 ± 1.2 s) is only slightly longer than that of 3′_A36_-xrRNA1-X (2.0 ± 0.2 s) (Supplementary Table S4).

### Mechanical anisotropy of ZIKV xrRNA1 probed by MD simulations

**Fig. 5:**
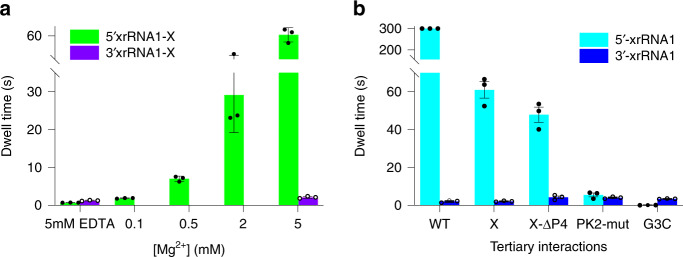
Mechanical anisotropy of ZIKV xrRNA1 is mainly dictated by its ring-like structure. **a** Plots of the dwell time for the unfolding and translocation of 5′_A36_-xrRNA1-X or 3′_A36_-xrRNA1-X in the α-HL nanopore against Mg^2+^ concentrations. **b** Plots of the dwell time for the unfolding and translocation of xrRNA1 mutants in the α-HL nanopore in the presence of 5 mM Mg^2+^. The dwell time for each of 3 independent experiments (*n* = 3), the mean and SEM are presented as dot, column, and error bar, respectively. Source data for **a** and **b** are provided as a Source Data file.

Plotting the duration time of 5′→3′ and 3′→5′ directional translocation of ZIKV xrRNA1-X through the nanopore against Mg^2+^ concentrations clearly shows that the presence of high concentration of Mg^2+^ significantly promote the mechanical anisotropy in ZIKV xrRNA1 (Fig. 5a), but the mechanical anisotropy is attenuated by disruption of the key tertiary interactions, even in the presence of Mg^2+^ (Fig. 5b). Both Mg^2+^ and the tertiary interactions are important to facilitate xrRNA1 folding into its ring structure, suggesting that the mechanical anisotropy of ZIKV xrRNA1 correlates with the structural integrity of its ring-like topology structure. To further explore the hypothesis and gain insights into the mechanisms underlying ZIKV xrRNA1 mechanical anisotropy, we used SMD simulation to investigate the mechanical unfolding of the ZIKV xrRNA1.

**Fig. 6:**
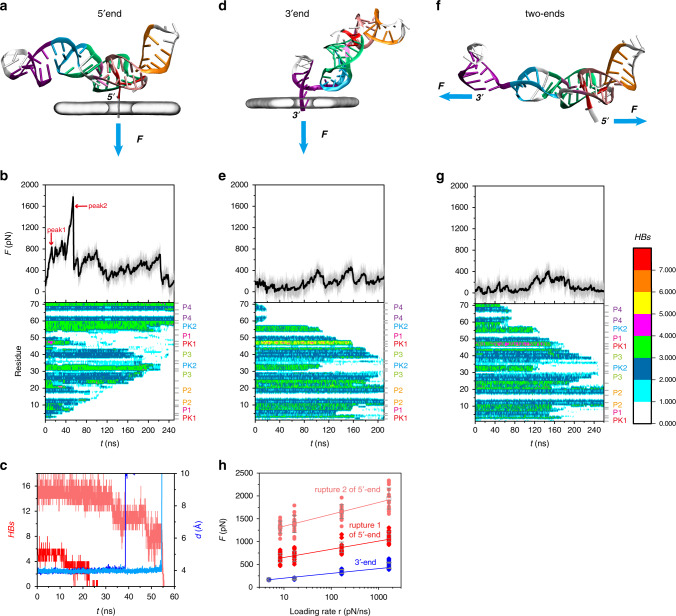
SMD simulations for ZIKV xrRNA1. **a**, **d**, **f** Schematic illustraion of translocation from 5′-end (**a**), 3′-end (**d**) through the nanopore, and two-ends pulling (**f**). **b**, **e**, **g** Unfolding force profiles (top) and hydrogen bonds (HBs) for each residue (bottom) in xrRNA1 over the course of pulling at loading rate of 166.1 pN/ns (*r* = *κ*·*ν*, pulling velocity *ν* and spring constant *κ* are 0.1 nm/ns and 1661 pN/nm, respectively) along 5-end (**b**), 3′-end (**e**), and two-ends pulling direction (**g**). **c** Hydrogen bonds of 5′-end structure (red) as well as P1 (pink), and sum of distance between Mg^2+^ with two phosphorous oxygen consisting Mg^2+^-clamps (blue: C5-Mg^2+^-C23; sky blue: A6-Mg^2+^-C23) during initial unfolding along 5′→3′ direction. (**h**) Dependence of averaged rupture forces on loading rates for 5′-end pulling of xrRNA1 and 3′-end pulling of xrRNA1-∆P4. The rupture forces and their mean with SD for each unfolding event at different loading rates are presented as point and box whisker, respectively. Source data for **b**, **e**, **g**, and **h** are provided as a Source Data file.

We first studied the unfolding of ZIKV xrRNA1 through an artificial nanopore system (see “Methods”) induced by the 5′-end pulling at loading rate of 166.1 pN/ns (Fig. 6a). ZIKV xrRNA1 exhibits high mechanical stability against external 5′-end pulling, and multiple force peaks are observed at the initial unfolding regime (0~60 ns). Further inspection reveals that these major force peaks are associated with two unfolding events: (1) breakage of one Watson–Crick base pair G3-C44 (PK1) along with one Hoogsteen pair U4·A24 within base triple U4·A24-U42 (referred to as 5′-end structure) at *t* ~ 23 ns (peak1); (2) rupture of P1 helix as well as the Mg^2+^-clamps (Mg^2+^ bridges two long-range phosphorous moieties C5-Mg^2+^-C23 and A6-Mg^2+^-C23) at *t* ~ 55 ns (peak2) (Fig. 6b, c). To get better statistics on mechanical properties, we emphasized our computation on initial unfolding regime of xrRNA1 and conducted a serial of SMD simulations at various loading rates, ranging from 8.3 to 1661 pN/ns. Sixteen independent simulations were performed for each loading rate, aggregating around 27 μs simulation. As a result, unfolding behaviors are well retained among all simulations across various loading rates (Supplementary Fig. S15). The average rupture forces for both major unfolding events appear to be logarithmic dependence on the loading rates, consistent with observation in dynamics force spectroscopy experiments (Fig. 6h).

**Fig. 7:**
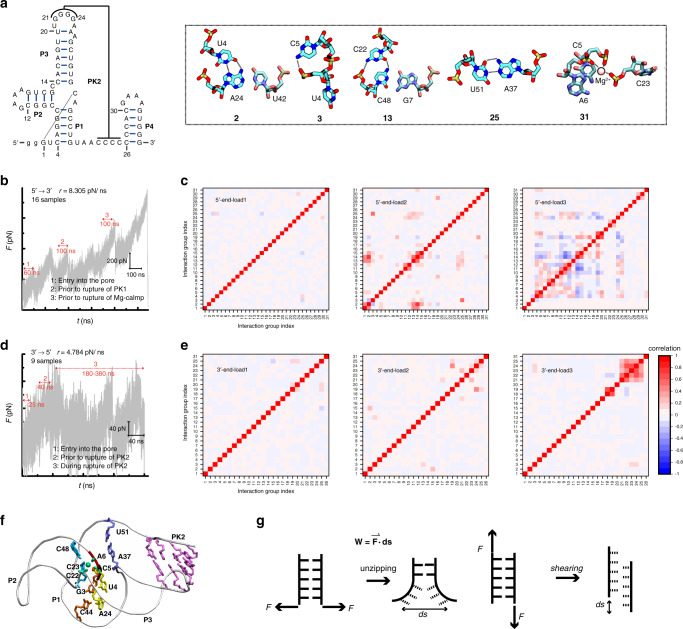
Structural origin of the mechanical anisotropy of ZIKV xrRNA1. **a** The interactions (native HBs and Mg^2+^ clamps) belonging to the same residue–residue pair are combined into a group, which is renumbered according to the secondary structure (left). A detailed view of non-canonical base pair interactions as well as the Mg^2+^ clamps is also included. **b**, **d** Definition of different loading stages for 5′-end pulling (**b**) and 3′-end pulling (**d**). **c**, **e** Correlation analysis of residue–residue interaction for 5′-end pulling (**c**), 3′-end pulling (**e**) at 3 different loading stages. **f** The intricate interaction network (or force-bearing structure) in the center of ring structure is showed in bonds, which is proposed to be responsible for the stronger mechanical resistance of xrRNA1 to 5′-end loading, whereas the 5′-stretch and PK2 enclosing the ring are highlighted in red and cyan, respectively. **g** Schematic illustration of shearing mode and unzipping mode. Source data for **c** and **e** are provided as a Source Data file.

It is quite surprising that the rupture force for one G3-C44 pair together with one U4·A24 Hoogsteen pair from 5′-end at loading rate of 166.1 pN/ns, is almost twice as the force required to unravel a full-length xrRNA1 along opposite direction (~800 pN vs. ~400 pN; Fig. 6h, e). To understand the source of strong mechanostability, we utilized the cross-correlation based native contact analysis to measure the force distributions on xrRNA1 during loading, which the interactions in xrRNA1 belonging to the same residue–residue pairs and the Mg^2+^ clamps are classified into 31 groups (Fig. 7a). Similar method, such as the dynamical network analysis, has been employed to study force/signal propagation in macromolecules^32–34^. We calculated the correlation among contact numbers to find out the force-bearing structures or motifs. As shown in Fig. 7b, c, when RNA enters into the nanopore immediately (stage 1: loading force is lower), cross-correlation is close to zero, thus the fluctuation of contact number among different groups is more stochastic. As translocation proceeds to a regime prior to rupture of PK1 (stage 2), the coupling among interaction groups became more apparent. The tertiary interaction networks together with PK2 which finally encloses the ring, could redistribute the loading force, and create confinement to restrict the movements of 5′-end and its interacting partner, making it to be unfolded in shearing manner (Fig. 7f). Such effect was regarded to play a crucial role in extreme mechanostability of pathogen adhesion^32^. From a simple mechanical perspective, a smaller displacement (*ds*) require a larger force to do a certain mechanical work W = F·ds (Fig. 7g). During unfolding, shearing rupture involved a smaller displacement compared to unzipping rupture, thus require a larger force than that of unzipping^33,35^. In addition, the stacking of unpaired A45 on G3-C44 pair (Supplementary Fig. S15e) contributes to the mechanostabiliy of 5′-end structure through shielding solvent to decrease kinetics of water insertion into hydrogen bonds region of base pair^36,37^. Such cooperative unfolding of 5′-end structure become more populated at lower loading rate *r* = 16.61 or 8.305 pN/ns (Supplementary Fig. S15f, g). As expected, the force distribution on ring structure of xrRNA become more pronounced with the increasing of loading force (stage 3). Accumulation of stress on xrRNA1 structure eventually leads to large scale destruction of P1 and Mg^2+^-clamps.

By contrast, the rupture force for xrRNA1 unfolding induced by 3′-end pulling at loading rate of 166.1 pN/ns is much lower and no larger than 400 pN (Fig. 6d, e and Supplementary Fig. S16a). This result further confirms the extreme mechanical anisotropy of xrRNA1 as probed by nanopore-sensing experiments (Fig. 5). As the 3′-end is threaded into the nanopore in an order of P4-PK2-P1-PK1/P3, the remainder of xrRNA1 outside of the nanopore undergoes translation and rotation, such that the nucleobases in pairs, which immediately enter into nanopore, are closely parallel to the pulling direction, rendering these base pairs prone to be broken with smaller rupture force in an unzipping manner (Supplementary Fig. S16c). To evaluate the mechanical stability of xrRNA1 unfolding along 3′→5′ direction with sufficient statistics, we made a trade-off to probe the rupture force of PK2 in xrRNA1-ΔP4 instead of the full-length xrRNA1. As deletion of P4 causes minor effects on the structure and mechanical properties of xrRNA1 (Supplementary Fig. S11), simulations on the xrRNA1-ΔP4 circumvents the larger computational cost required for the full-length xrRNA1. 53 trajectories were collected for PK2 rupturing in xrRNA1-ΔP4 at various loading rates ranging from 4.784 to 1661 pN/ns (Supplementary Table S5), totaling ~6 μs of simulation time. As shown in Supplementary Fig. S17, multiple rips can be found in the sawtooth-shaped force profiles, which are associated with stepwise decrease in hydrogen bonds of PK2, thus featuring unfolding in an unzipping manner. The average rupture forces for PK2 in xrRNA1-ΔP4 along 3′→5′ direction are much smaller than that in opposite direction, but also roughly follow a logarithmic scaling with the loading rates (Fig. 6h). The native contact analysis reveals that structural perturbation induced by external force from 3′-end is limited to the local segment (PK2) entering into the pore (Fig. 7d, e), consistent with an unzipping mode to resolve the RNA duplex by RdRP from the 3′→5′ direction. In addition, we made a rough estimation of stress distribution on xrRNA1 by FDA methods^38^ combined with effective contact energy functions from Structure-based model SMOG (see “Methods,” Supplementary Fig. S18 and Supplementary Information for more details)^39,40^. As shown in Supplementary Fig. S19, the changes of stress distribution on xrRNA1 during translocation with respect to equilibrium MD simulation (Supplementary Fig. S20) are mainly restricted in local region (PK2 we explored), in contrast to a broader changes of stress distribution on ring structure of xrRNA1 from opposite direction. Recently, an MD simulation work employed SMOG model in combination with strain analysis (based on native contact fraction) also revealed extraordinary mechanical anisotropy of ZIKV xrRNA1^41^. Interestingly, these approaches based on different models (AMBER14 and SMOG) and analysis methods provide consistent picture about the origin of anisotropic response to loading, pinpointing the importance of geometrical constrains imposed by ring structure for the function of xrRNA1 (further details are provided in the Supplementary Information). For comparison, we also performed conventional two-end stretching simulations for xrRNA1 at loading rate of 166.1 pN/ns (Fig. 6f, g and Supplementary Fig. S16d). As expected, unfolding of xrRNA1 by two-end pulling initiates from the weaker 3′-end and in an unzipping manner, similar to the observation in 3′-end translocation-coupled unfolding.

Taken together, by combining SMD simulation and FDA, we found that when external forces are loaded to the 5′-end of xrRNA1 encircled by a ring, the forces are distributed on the ring structure through its tertiary interaction network around 5′-end, rendering its extraordinary mechanical stability. Upon forces exerted on the 3′-end, which is absent of such tertiary interactions network, xrRNA1 prefers to be unfolded in unzipping fashion, lowering its mechanical resistance. The mechanical anisotropy of xrRNA1 revealed by MD simulations is thus consistent with the nanopore-sensing experiments. It should be noted that we cannot rule out the possibility that the unfolding pathway of xrRNA1 may be changed by the loading forces, as seen in previous study^42^. Due to the cooperative coupling among the interaction networks, such structure may experience cooperative unfolding in response to external forces from 5′-end, resulting in a larger translocation barrier upon loading forces lowered to the scale of nanopore-sensing experiments (more discussions can be found in Supplementary Information).

## Discussion

In this study, we use the single-molecule nanopore-sensing technique to investigate the directional mechanical stability of a viral RNA with ring-like structure, which the single-stranded leader sequences ensure unfolding-coupled translocation to be carried out in defined direction, therefore can better mimic the directionality of the cellular processes. We find that the mechanical stability of ZIKV xrRNA1 is dependent on the direction of the leader-guided translocation through the nanopore. In the presence of 5 mM Mg^2+^, the duration time of the translocation through the nanopore from the 5′→3′ direction is more than 150 times longer than that from the 3′→5′ direction, confirming extreme mechanical anisotropy in ZIKV xrRNA1. The extreme mechanical anisotropy is consistent with its functional activities in resisting degradation by cellular exonuclease Xrn1 to generate sfRNAs but readily unfolding as template for negative strand synthesis and genome replication induced by viral RdRP, therefore is of high biological significance. The nanopore-sensing experiments show that the mechanical anisotropy of ZIKV xrRNA1 highly depends on Mg^2+^, which is a result of high dependence of 5′→3′ directional mechanical stability but no obvious dependence of 3′→5′ directional mechanical stability on Mg^2+^. The possessing of controllable mechanical stability and regulable mechanical anisotropy is important for biomolecules’ biomedical applications.

Mg^2+^ may facilitate xrRNA1 folding and structure and modulate the mechanical stability through both direct binding and acting as counterions to allow intramolecular tertiary interactions by reducing repulsive forces^43^. Crystal structure of ZIKV xrRNA1 has identified one Mg^2+^ ion-binding site, mutations on the binding sites (C5G, A6U, and C22G) results in loss of Xrn1 resistance ability, larger *R*_*g*_ and *D*_max_, improper folding and increased flexibility even in the presence of 5 mM Mg^2+^ by SAXS (Supplementary Fig. S21 and Supplementary Table S3), supporting the importance of direct Mg^2+^ ion binding to xrRNA1 structure and folding. Mutational perturbation has also identified the importance of tertiary interactions such as PK1 and PK2 in xrRNA1 structure and mechanical stability; however, the formation and stabilization of such tertiary interactions must require Mg^2+^. In the absence of Mg^2+^, ZIKV xrRNA1 adopts unfolded structure and no such tertiary interactions are formed, supporting the critical roles of Mg^2+^ as counterions in shielding negative charges and reducing repulsive forces, therefore stabilizing such tertiary interactions. These results are also consistent with the DSC data, which show a more sensitive dependence of tertiary interactions on Mg^2+^ (Supplementary Fig. S3).

The ring-like architecture and the key tertiary interactions in xrRNAs are highly conserved across diverse mosquito-borne flaviviruses. The presence of duplicated xrRNA structures in the 3′-untranslated region is a common feature to most of the mosquito-borne flavivirus RNA genomes^44^. Recent research indicates that xrRNAs are also widespread in coding and noncoding subgenomic RNAs of two large families of plant-infecting RNA viruses^45^. The intricate mechanical anisotropy endowed by the ring-like architecture of xrRNAs may represent a more general strategy for RNA maturation and maintenance in many vectorial processes than previous known. Our nanopore-sensing experiments also show that disruption of the key tertiary interactions in ZIKV xrRNA1 affects the 5′→3′ directional mechanical stability significantly, but has no obvious effects on the 3′→5′ directional mechanical stability; therefore, modulation of tertiary structure during infection such as mutations may mainly regulate sfRNA production but have minimal effect on flavivirus replication. A recent report showed that structural changes in the xrRNA structures of the dengue virus genome facilitates virus’s rapid adaptation to mosquito and human; moreover, adaptive mutations and deletions during host switch were mainly accumulated in regions that would effectively abrogate tertiary interactions^46^. It is interesting to know how these mutations and deletions affect sfRNAs production in infected hosts, although having not been studied yet. sfRNAs production have been implicated to impact on flavivirus replication, cytopathicity, and pathogenicity^10,11^. We speculate that sfRNAs production modulated by the tertiary structural changes upon host switch is related to the viruses’ ability to adapt to different hosts.

Understanding the mechanisms that determine RNA mechanical anisotropy could be of great importance in development of RNA-based mechanically anisotropic biomaterials. Due to its diverse structural and functional attributes, RNA has recently attracted widespread attention as a unique biomaterial with distinct biophysical properties for designing sophisticated architectures in the nanometer scale.^47^ For example, the φ29 pRNA 3WJ has been used as building block to construct RNA triangles, squares, pentagons, and hexagons, as a platform for building a variety of multifunctional nanoparticles as potential therapeutic agents^47–49^. Mechanical anisotropy is an essential property for many soft biological tissues. The microstructures of many such tissues, such as the fibers of the human Achilles tendon, often run parallel to each other in a specific direction, leading to anisotropy in the mechanical properties of the tissues, therefore have been identified as typical anisotropic materials^50^. However, it remains a big challenge to build biomimetic materials with mechanical anisotropy based on macromolecules. Discovering molecular building blocks with anisotropic mechanical properties and revealing the structural mechanisms of mechanical anisotropy is the key step to overcome this challenge. Our work shows that the mechanical anisotropy of a flaviviral RNA is largely defined by the structural integrity of its ring-like topological structure, which is regulated by Mg^2+^ and the key tertiary interactions that act cooperatively to facilitate RNA folding. Interestingly, the mechanical anisotropy of ZIKV xrRNA1 can be reversibly regulated by Mg^2+^, although most of the mechanically anisotropic biomaterials cannot respond to external stimuli^51^. Therefore, ZIKV xrRNA1 could be ideal building blocks for environmental responsive anisotropic artificial tissues. It’s expected that more ring-like RNA structures with mechanical anisotropy could be identified and designed for the development of RNA-based mechanically anisotropic biomaterials.

## Methods

### RNA sample preparation

The wild-type ZIKV xrRNA1 and its mutant constructs were generated as follows. Plasmids coding an upstream T7 promoter, the ZIKV xrRNA1 sequence with its upstream or downstream polyA sequences, were gene synthesized and sequenced by Wuxi Qinglan Biotechnology, Inc., Wuxi, China. All the other mutants were generated using the Transgen’s Fast Mutagenesis System. The double-stranded DNA fragment templates for in vitro RNA production were generated by PCR using an upstream forward primer targeted the plasmids and a downstream reverse primer specific to respective cDNAs. The RNAs were transcribed in vitro using T7 RNA polymerase and purified by preparative, non-denaturing polyacrylamide gel electrophoresis, the target RNA bands were cut and passively eluted from gel slices into buffer containing 0.3 M NaAc, 1 mM EDTA pH 5.2 overnight at 4 °C. The RNAs were further passed through the size-exclusion chromatography column to final buffer condition for nanopore sensing, DSC, SAXS, and other biochemical experiments. The sequences for all the constructs and primers used in this study are listed in Tables S6 and S7.

### Differential scanning calorimetry

All DSC measurements were performed using a VP DSC Micro-calorimeter from the Malvern MicroCal (Northampton, MA). The DSC consists of a matched pair of 0.511 ml sample and reference cells. In a series of DSC scans, both cells were first loaded with the buffer solution, equilibrated at 20 °C for 15 min, and scanned from 20 to 100 °C at a scan rate of 100 °C/h. The buffer vs. buffer scan was repeated for five times to obtain baseline, then the sample cell was emptied, rinsed, and loaded with the RNAs solution prior to the 15 min equilibration period. RNA samples were kept at a concentration of 30 μM and in buffers containing 10 mM sodium phosphate, 150 mM KCl pH 7.5, and various concentrations of Mg^2+^, and care was taken to minimize the presence of air bubbles in loading of the sample cell. The experimental data are processed by subtracting the baseline first, then normalizing the concentration and using a non-two-state model to deconvolute the curve by using OriginPro2016 software; finally, the melting temperature *T*_m_, which relates to thermal stability, was obtained directly^52^.

### Small-angle X-ray scattering

All the parameters for data collection and software employed for data analysis are summarized in Table S8. SAXS measurements were carried out at room temperature at the beamline 12 ID-B of the Advanced Photon Source, Argonne National Laboratory or the beamline BL19U2 of the National Center for Protein Science Shanghai and Shanghai Synchrotron Radiation Facility. The scattered X-ray photons were recorded with a PILATUS 2M detector (Dectris) at 12 ID-B and a PILATUS 100k detector (Dectris) at BL19U2. The setups were adjusted to achieve scattering *q*-values of 0.005 < *q* < 0.89 Å^−1^ (12 ID-B) or 0.009 < *q* < 0.415 Å^−1^ (BL19U2), where *q* = (4*π*/*λ*)sin(*θ)*, and 2*θ* is the scattering angle. Thirty two-dimensional images were recorded for each buffer or sample solution using a flow cell, with the exposure time of 0.5–2 s to minimize radiation damage and obtain good signal-to-noise ratio. No radiation damage was observed as confirmed by the absence of systematic signal changes in sequentially collected X-ray scattering images. The two-dimensional images were reduced to one-dimensional scattering profiles using MatlabR2017a (12 ID-B) or BioXTAS Raw1.3.1 (BL19U2). Scattering profiles of the RNAs were calculated by subtracting the background buffer contribution from the sample-buffer profile using the program PRIMUS3.2^53^ following standard procedures. Concentration series measurements (four- and twofold dilution and stock solution) for the same sample were carried out to remove the scattering contribution due to inter-particle interactions and to extrapolate the data to infinite dilution. The forward scattering intensity *I*(0) and the radius of gyration (*R*_g_) were calculated from the data of infinite dilution at low q-values in the range of *qR*_g_ < 1.3, using the Guinier approximation: ln*I*(*q*) ≈ ln(*I*(0)) − *R*_g_^2^*q*^2^/3. These parameters were also estimated from the scattering profile with a broader *q* range of 0.006–0.30 Å^−1^ using the indirect Fourier transform method implemented in the program GNOM4.6^54^, along with the PDDF, *p*(*r*), and the maximum dimension of the protein, *D*_max_. The parameter *D*_max_ (the upper end of distance *r*), was chosen so that the resulting PDDF has a short, near zero-value tail to avoid underestimation of the molecular dimension and consequent distortion in low resolution structural reconstruction. The volume-of-correlation (*V*_c_) were calculated using the program Scatter and the molecular weights of solutes were calculated on a relative scale using the *R*_g_/*V*_c_ power law developed by Rambo et al.^55^, independently of RNA concentration and with minimal user bias. The theoretical scattering intensity of the atomic structure model was calculated and fitted to the experimental scattering intensity using CRYSOL2.8.3^56^.

### Xrn1 resistance assay

The Xrn1 resistance experiments were conducted by following the standard protocol developed in previous studies^13,14^. Recombinant Xrn1 from *Kluyveromyces lactis* and RppH from *Bdellovibrio bacteriovorus* were expressed in *Escherichia coli* and purified by Ni^2+^-NTA affinity and size-exclusion chromatography. In a 10 μl reaction, ~600 ng purified RNA was treated with 0.5 μl of >3 U/μl RppH and then split between two tubes. Then, 0.5 μl of >3 U/μlXrn1 was added to one half of the reaction, whereas the other served as a (−) Xrn1 control, followed by incubation at 37 °C for 30 min. Reactions were then quenched by the addition of an equal volume of a TBE-Urea loading dye containing 30 mM EDTA, 8 M Urea, and 0.1% (wt/vol) xylene cyanol and bromophenol blue. Then the RNA products were analyzed on 8% TBE-Urea denaturing gel and visualized by staining with Gel safe.

### Single-molecule nanopore sensing and data analysis

Single-channel current recordings were performed with an individual α-HL nanopore inserted into a vertical lipid bilayer. The vertical chamber setup was assembled by two compartments, a cuvette with a 150 μm aperture drilled on the side (*cis*) and a bilayer chamber (*trans*) (Warner Instruments, Hamden, CT, USA). After pre-painting both sides of the cuvette aperture with 0.5 mg/mL DPhPC/hexane, both chambers were filled with 1 mL of test buffer (20 mM Tris pH 7.5, 1 M KCl (*cis*)/3 M KCl (*trans*) with different Mg^2+^ concentrations). RNAs were added to the *cis* solution with a final concentration of 50 nM. The lipid bilayer was created by applying 30 mg/mL DPhPC/decane to the pretreated aperture. In vitro-assembled WT-α-HL nanopore protein was added in the *cis* compartment. After a single protein nanopore was formed in the lipid bilayer, positive potentials of 100 mV were applied across the lipid bilayer with Ag/AgCl electrodes. The *cis* compartment was defined as the virtual ground. The electrical current was recorded with a patch-clamp amplifier (HEKA EPC10; HEKA Elektronik, Lambrecht/Pfalz, Germany). Recordings were collected using a 3 kHz low-pass Bessel filter at sampling frequency of 20 kHz with a computer equipped with a LIH 1600 A/D converter (HEKA Elektronik). All measurements were carried out at room temperature. Data analysis was performed using MATLAB (R2017a, MathWorks) software and OriginPro2016 (OriginPro Corp., Northampton, MA, USA). The current blockades are described as *I*/*I*_0_, where *I*_0_ is the ionic current of open nanopore and *I* is the blockage current produced by the analyte. Events with current blockades larger than 70% and dwell time longer than 0.15 ms were analyzed as RNA translocation events. The mean dwell time for current spikes was obtained from the dwell time histograms. All the data are presented as mean ± SEM of three independent experiments.

### Molecular dynamics simulations

In the spirit of nanopore-sensing experiments, we perform SMD simulations in an artificial nanopore system, which was modeled by one graphene layer with a 13 Å diameter pore in its center and is comparable to the pore size of α-HL^22^. Such simple nanopore employed in simulation avoids the putative complicated interactions between the pore and RNA, favorable to explore the intrinsic mechanical characteristics of RNA during translocation. The ZIKV xrRNA1 (PDB code: 5TPY) was used in our simulations^14^. RNA was subjected to equilibrium simulations in an isolated system, following the similar protocol in the previous study^6^. After 100 ns equilibrium simulations without any restraint, the equilibriumed RNA configuration along with the water in first hydration shell (the cutoff distance is 3.5 Å) was extracted to be used as a starting structure for subsequent simulations. Then the RNA structure was translated and rotated so that the pulling terminus (5′- or 3′-terminus) was above the pore. The merged system (after adding solvent and ions) was further equilibriumed within NPT ensemble for 10 ns with positional restraint on the pulling terminus, allowing RNA to further relaxation and free rotation around the anchor. Then the external force was loaded on the chosen terminus of RNA using SMD simulation, to drive RNA thread through the nanopore. The position restraints were imposed on the nanopore, to mimic immobilization of the pore. For comparison, we also performed two-end loading simulations for xrRNA1.

All simulations were conducted using GROMACS 2016.4 or 2018.6 packages^57,58^.The parameters for the RNA and monovalent ions were taken from the AMBER14 Force Field and used in concert with the TIP3P explicit water model^59–62^. The Mg^2+^ ion model parameterized by Villa and colleagues^63^ was used in simulations. The parameters for nanopore were adapted from the aromatic carbon atom in AMBER Force Field (e.g., CA) and the atomic charge is set to zero as previous computation works^35,64^. To avoid the putative sticking of aromatic ring (i.e., RNA base) on nanopore surface, the interaction strength (*ε*, the depth of Lennard–Jones potential) between nanopore and RNA were reduced to one tenth of the original. Monovalent ions (K^+^ and Cl^−^) were added to neutralize the system, yielding an ionic concentration of 150 mM. Periodic boundary conditions were applied to all the systems in three directions. van der Waals and short-range electrostatic interactions were calculated at a cutoff distance of 10 Å, whereas long-range electrostatic interactions were computed by the particle mesh Ewald method^65^. The temperature (*T* = 310 K) and pressure (*P* = 1 atm) were maintained using a stochastic velocity rescaling thermostat and Parrinello–Rahman barostat, respectively. All SMD simulations were run in NVT ensemble at a temperature of 310 K. Structural visualization and partial analysis were performed using VMD1.93^66^. The eRMSD^67^ and native contacts analysis were calculated with PLUMED2.5.1^68^.

### Cross-correlation-based native contacts analysis

Generally speaking, the structural distortion in response to loading force can be in principle measured using appropriate collective variables (such as internal distance and coordination number, etc.). Upon force loaded onto molecules, the atoms or residues in the force propagation way tend to move in concert^33,34,38^. Herein, we calculated the correlation among native contact numbers from different groups. We combined the interactions belonging to the same residue–residue pair into a unit (group). In total, 31 residue–residue interaction groups were employed in our correlation analysis, including canonical base pairing (stems, pseudoknot PK1, and PK2), non-canonical tertiary interactions (hydrogen bonds), as well as coordination of Mg^2+^ with phosphorous oxygen (Mg^2+^-C5, Mg^2+^-A6, Mg^2+^-C23, all merged into a group). Whereas the non-canonical interactions within GAAA apical loops were not taken into account during our analysis, due to that these loops are located distal to the center of xrRNA1.

The native contact number for each contact pair is calculated as *n*_*i,j*_(*r*) = 1/[1 + (*r*/*r*_0_)^6^], where *r* is the distance between atom *i* and *j*, and *r*_0_ are set as 4 or 2.6 Å for hydrogen bond and coordination of Mg^2+^ with phosphorous oxygen atoms, respectively. Then, the native contact number is transformed as *n*′_*i,j*_ (*r*) = [*n*_*i,j*_(*r*) − *n*_*i,j*_(*d*_max_)]/[*n*_*i,j*_(0) − *n*_*i,j*_(*d*_max_)], allowing the contact number smoothly decay to zero when *r* ≥ *d*_max_ (detailed information can be found in the manual of PLUMED). The cutoff *d*_max_ for hydrogen bond and coordination with Mg^2+^ are set as 5 and 3.6 Å, respectively. The correlation between native contact group A and B is defined as *Cov*(*A,B*) = < [*N*_*A*_(*t*) − <*N*_*A*_(*t*)>]*[*N*_*B*_(*t*) − <*N*_*B*_(*t*)>]>/[*σ*_NA_*σ_*NB*_], where < · > denotes the average, *N*(*t*) is the total contact number within residue–residue group at time of *t*, and *σ* is the SD of *N*(*t*).

### Force distribution analysis

In general, the intramolecular force is regarded to be sensitive to change of structure, especially for the rigid core of biomacromolecules^38^. We also used FDA method^38^ to dissect mechanism underlying the mechanical anisotropy of xrRNA1. The potential energy functions from all-heavy-atom structure-based model (SMOG)^39,40,69^ rather than from explicit solvent force field, which is used in our SMD simulations (i.e., AMBER14), were leveraged to evaluate the strength of stress on these conformational ensembles from explicit solvent SMD simulations. Such treatment avoided calculation for solvent screening effect on electrostatic interaction among atoms, which is important to handle highly charged molecules like RNA. Within structure-based model, these interactions (hydrogen bond, hydrophobic contact, salt-bridge, etc.) observed in native structure and the solvation effect are approximately described by native contacts, driving the macromolecule to fold into native state with minimal frustrations. Structure-based model also has been employed to explore mechanical properties and folding of macromolecules.

A centroid structure of 100 ns explicit solvent MD simulation (equilibrium MD, EQ), in which A53 restores stacking on A52, and meanwhile GAAA tetraloops capped on P2 and P4, all maintain native state (Supplementary Fig. S19e-f and Supplementary Text), was chosen to generate topology file for xrRNA1 using SMOG server (http://smog-server.org/)^40^. The native contacts are determined by the default setting, i.e., Shadow Contact algorithm with distance cutoff of 6 Å^69^. In addition, we performed sets of trial simulations to check whether structure-based model could capture essence of translocation-coupled unfolding for ZIKV xrRNA1, prior to employing the model for FDA. Full details regarding SMD simulation based on SMOG model can be found in Supplementary Information.

We used GROMACS2018.7-FDA2.9 (a modified version of GROMACS2018.7, https://github.com/HITS-MBM/gromacs-fda/releases) to compute the residue–residue pairwise forces according to atomic interaction energy functions (non-bonded) from SMOG, for the conformation ensemble from our explicit solvent MD simulation, *F*_*I*,*J*_ $= \mathop {\sum }\nolimits_{i,j}^{} F_{i,j}$, where atoms *i* and *j* belongs to residue *I* and *J*, respectively. The average stress for residue *I*, is calculated as the sum of absolute value of averaged residue–residue pairwise force on residue *I*, < *F*_*I*_ > ... and then relative stress change under loading (SMD) with respect to unloading (EQ) is defined, *S*(*I*)_load_ = [<*F*_*I*_ > _load_ − <*F*_*I*_ > _EQ_]/<*F*_*I*_ > _EQ_. An overview of FDA combined with SMOG model is shown as a flowchart in Supplementary Fig. S18a.

### Reporting summary

Further information on research design is available in the Nature Research Reporting Summary linked to this article.

## Supplementary information

Supplementary Information

Peer Review File

Reporting Summary

## Data Availability

Data supporting the findings of this manuscript are available from the corresponding authors upon reasonable request. A reporting summary for this Article is available as a Supplementary Information file. Source data are provided with this paper.

## Source data

Source Data

## Code availability

Custom scripts used to analyze the single-molecule nanopore data and MD simulations data are provided with this paper.
