# Supplementary material for: Molecular mechanisms underlying the extreme mechanical anisotropy of the flaviviral exoribonuclease-resistant RNAs (xrRNAs)
Source: Nat Commun. 2020 Oct 30;11:5496. doi: 10.1038/s41467-020-19260-4 (PMC7603331; doi:10.1038/s41467-020-19260-4)
Supplement: Supplementary file 1 — Supplementary Information [file 41467_2020_19260_MOESM1_ESM.pdf]

Supporting Information

Molecular mechanisms underlying the extreme mechanical anisotropy of the flaviviral exoribonuclease-resistant RNAs (xrRNAs)

Xiaolin Niu[1, 3], Qiuhan Liu[2, 3], Zhonghe Xu[1, 3], Zhifeng Chen[1], Linghui Xu[1], Lilei Xu[1], Jinghong Li[2, *], Xianyang Fang[1, *]

[1]Beijing Advanced Innovation Center for Structural Biology, School of Life Sciences, Tsinghua University, Beijing 100084, China.

[2]Department of Chemistry, Key Laboratory of Bioorganic Phosphorus Chemistry & Chemical Biology, Tsinghua University, Beijing 100084, China.

[3]These authors contributed equally: Xiaolin Niu, Qiuhan Liu and Zhonghe Xu.

[*]email: fangxy@mail.tsinghua.edu.cn; jhli@mail.tsinghua.edu.cn

Table of Contents

Extended Section 3
Supplementary Figure 1 9
Supplementary Figure 2 10
Supplementary Figure 3 11
Supplementary Figure 4 12
Supplementary Figure 5 13
Supplementary Figure 6 14
Supplementary Figure 7 15
Supplementary Figure 8 16
Supplementary Figure 9 17
Supplementary Figure 10 18
Supplementary Figure 11 19
Supplementary Figure 12 20
Supplementary Figure 13 21
Supplementary Figure 14 22
Supplementary Figure 15 23
Supplementary Figure 16 24
Supplementary Figure 17 25
Supplementary Figure 18 26
Supplementary Figure 19 27
Supplementary Figure 20 28
Supplementary Figure 21 30
Supplementary Table 1 31
Supplementary Table 2 32
Supplementary Table 3 33
Supplementary Table 4 34
Supplementary Table 5 35
Supplementary Table 6 36
Supplementary Table 7 37
Supplementary Table 8 38
References 39

EXTENDED SECTION

This section is intended to provide an elaborate discussion on certain results in the main text that had to keep brief because of length restrictions.

Mg^{2+}-dependent structural transition and thermal stability of ZIKV xrRNA1. The formation of stable and compact RNA structure requires the neutralization of polyanionic phosphate backbone by counterions[1]. An important question is the extent to which tertiary interactions stabilize the ZIKV xrRNA1 ring structure in the presence of different types of counterions, such as K^+, Na^+, Mg^{2+}, etc. Mg^{2+} ions are known to be important for the structure and stability of RNA molecules[2]. As shown in **Figure 1a-b**, a Mg^{2+} ion is found to coordinate with the phosphates of C5, A6 and C22 in the crystal structure of ZIKV xrRNA1, implying that Mg^{2+} ions may play an important role in the structure and folding of xrRNA1.

To understand how Mg^{2+} affect the overall structure and folding of ZIKV xrRNA1, small angle x-ray scattering (SAXS) experiments were carried out to follow the Mg^{2+}-induced structural transition. The representative scattering profiles, with scattering intensity $I(q)$ plotted against momentum transfer q, the Guinier fitting of the scattering profiles, along with pair distance distribution function PDDFs transformed from scattering profiles for ZIKV xrRNA1 in 20 mM Tris-HCl supplemented with different concentration of Mg^{2+}, are shown in **FigureS2a-c**. The overall structural parameters, including R_g calculated from Guinier slops, R_g and D_{max} from the PDDF functions, as well as molecular weights derived from volume-of-correlation (V_c)[3], are summarized in **Table S1**. Plotting the radius of gyration (R_g) values calculated from the PDDF functions against Mg^{2+} concentrations shows a cooperative change in R_g value, suggesting that ZIKV xrRNA1 undergoes a Mg^{2+}-induced two-state transition between the unfolded and folded states, and the midpoint of the transition is about 0.3 mM Mg^{2+} (**Figure S2e**). The fraction of unfolded RNA Φ_U was calculated at each Mg^{2+} concentration from $R^2_g = \Phi_U R^2_{g,U} + (1-\Phi_U)R^2_{g,F}$, with $R_{g,U}$ and $R_{g,F}$ being the radii of the gyration for the unfolded and folded RNA in 20 mM Tris-HCl and 5 mM Mg^{2+}, respectively (**Figure S2f**). The transition from an open, unfolded conformation to a globular, folded form is also apparent from the changes of Kratky plots of xrRNA1 in 0 to 10 mM Mg^{2+} (**Figure S2d**). In 20 mM Tris-HCl and the absence of Mg^{2+}, ZIKV xrRNA1 may form secondary structures such as duplexes but no discernible high-order structure, therefore ZIKV xrRNA1 is in an open, unfolded state

characterized with an average R_g of ~30Å. Upon increasing the Mg^{2+} concentration, ZIKV xrRNA1 becomes more compact, and the R_g shrink to ~23 Å at Mg^{2+} concentrations above ~1mM and doesn't change significantly between 2 to 10 mM Mg^{2+}, therefore ZIKV xrRNA1 mainly adopts a folded conformation with the tertiary structures formed, which is further supported by the fact that the experimental scattering profile at 5 mM Mg^{2+} fits nicely to the theoretical scattering curve calculated from the crystal structure[4]. The effect of monovalent cation, such as K^+ on the overall structure of ZIKV xrRNA1 is also probed. In comparison with that in 20 mM Tris-HCl and no Mg^{2+}, the average R_g value of xrRNA1 at 20 mM Tris-HCl, 100 mM K^+ deceases to ~24.45Å, but doesn't change significantly upon even higher concentration of K^+ such as at 20 mM Tris-HCl, 1000 mM K^+ (R_g=23.47Å) (**Table S1**). These results suggest that Mg^{2+} is generally a more potent stabilizer of the tertiary interactions than K^+ ions and essential to the overall structure of ZIKV xrRNA1.

The effects of Mg^{2+} on the overall thermal stability of ZIKV xrRNA1 was analyzed by differential scanning calorimetry (DSC) which can provide RNA stability in terms of melting temperature[5]. The DSC thermograms of ZIKV xrRNA1 are compared over a range of Mg^{2+} concentrations (**Figure S3a**). **Figure S3b** shows a typical DSC thermogram for ZIKV xrRNA1 in 0 mM Mg^{2+}, which can be further fitted into two distinct transition peaks with T_m values of 63.3, 75.3 ℃, respectively. The low- and high- temperature transitions can be attributed to unfolding of tertiary and secondary structures, respectively[6]. Both the low- and high- temperature transitions shift to high temperature as Mg^{2+} concentration increases, but the low- is more sensitive than the high- temperature transition to changes in Mg^{2+} concentrations, resulting in merging of these two transitions into one peak above 5 mM Mg^{2+} (**Figure S3c**) which xrRNA1 is thought to unfold in a cooperative manner. These results suggest an essential role of Mg^{2+} in stabilizing the tertiary interactions rather than the secondary structure in ZIKV xrRNA1.

Equilibrium MD simulation for ZIKV xrRNA1. We also performed 1450-ns MD simulation for ZIKV xrRNA1 without external force loaded. As shown in **Figure S20a-b**, xrRNA1 exhibits considerable flexibility, with an average all-heavy-atom root mean square deviation (RMSD) of ~ 4 Å. xrRNA1 undergoes twist motions along its long axis and bending motions between PK2 and P4, as revealed by

Principal Component Analysis (PCA) (**Figure S20c**). Structural alignment between the averaged structure derived from PCA analysis and the crystal structure indicates that the two structures are similar to each other with a RMSD of ~ 3 Å, and the major differences lie in PK2 and P4, which displays a slight tilt in the averaged structure relative to the crystal structure (**Figure S20d**). Such dynamic motions or structural flexibility observed during MD simulation of xrRNA1 reflects its intrinsically elastic properties. Additionally, we also detected some local structural changes from crystal structure during MD simulation. For example, A53 keeps well-stacked with A52 during the whole course of MD simulation, whereas A53 flips out in the crystal structure (**Figure S20e**). The GAAA tetraloops capped on helices P2 and P4 also exhibit considerable flexibility, switching between native and non-native state (eRMSD > 0.7) (**Figure S20f**). eRMSD is a metric developed by Bottaro and Bussi to measure distances between RNA structures, by which conformational differences could be better discerned when RMSD is below 4 Å[7]. Nonetheless, the contact map derived from MD simulation is highly similar to the one from crystal structure (**Figure S20 g, h**).

SMOG model for translocation-coupled unfolding of ZIKV xrRNA1-ΔP4. Prior to employing All-atom-structure-basedmodel (SMOG[8, 9, 10]) for force distribution analysis, we performed sets of SMD simulations to check whether structure-based model could capture essence of translocation-coupled unfolding for ZIKV xrRNA1.The truncated construct xrRNA1-ΔP4 was employed in our simulation. The bond angle force constants for nanopore were adapted from nucleobase in SMOG model. And the interactions between RNA and nanopore are repulsive as the treatment of non-native interactions in SMOG. Similar to the procedure of our explicit solvent SMD simulation (main text), position restraints were also imposed on the nanopore.

Langevin dynamics with constant pulling velocity (0.5 and 0.05 nm/ns) on the loading end (5'-end, or 3'-end) were conductedusing GROMACS2018.6, with spring constant of 166.1 pN/nm. For both translocation directions, four replicas were run for each pulling velocity. Non-bonded interactions were calculated at a cutoff distance of 12 Å. The temperature for all simulations was set 60 K as previous studies[8, 9, 10] (~0.6 T_m,T_m≈105 K is derived from parallel tempering simulation, data is not shown), whereas the integration time step is1 fs. It should be noted that the structural transition

observed in structure-based modeling generally could occur several orders of magnitude faster than that observed in physical force field-based simulation (i.e. AMBER), owing to that the absence of non-native interactions in the structure-based model make the free energy landscape much smoother and reduces the internal friction [8, 10]. Herein, we just compared different mechanical response between 5'→3' and 3'→5' translocation direction, thus we did not calibrate the physical units such as temperature, time step and force et al.

The unfolding processes probed by simulations using SMOG model, similar to that observed in the explicit solvent simulations, exhibit extreme mechanical anisotropy. The simultaneous rupture of 5'-end structure together with two Mg^{2+}-clamps and several base pairs of P1 encounters in 5'-3' translocation direction, yielding a larger unfolding force of 220 pN at loading rate of 8.305 pN/ns, almost 10 times of the unfolding force for PK2 (~21 pN) along opposite direction. Compared to explicit solvent simulation, the enhanced cooperativity among force-bearing structure under the loading from 5'-end maybe is the result of fast intrinsic transition rate of SMOG. Recently, a MD simulation work using SMOG model by Suma et al also demonstrate that the intricate network around 5'-end of ZIKV xrRNA1 could redistribution force from 5'-end, rendering its translocation resistance; whereas such mechanism doesn't work for 3'-end, which could be readily ruptured along opposite direction[11]. The agreement between our results (including explicit solvent MD and SMOG MD) and recent work by Suma indicates that geometrical feature of ring-like architecturerather than particular energetic interactions defines the anisotropic response of ZIKV xrRNA1 to mechanical force.

Taken together, structure-based model is simple and omits complex interactions such as electrostatic interaction and solvation et al, however, it still can work to capture the structural determinant for mechanical properties of xrRNA1. We will leverage the Hamiltonian from SMOG model to roughly assess intramolecular stress of xrRNA1 under loading, bypassing the requirements to reliable estimation of solvent shielding effect on RNA.

Comparison of timescale in MD simulations and nanopore experiments. It should be noted that to characterize the unfolding events of ZIKV-xrRNA1 in tractable simulation time, we employed a loading rate with several orders of magnitude higher

than the experimental one, causing the unfolding rate in simulations much faster than that in nanopore sensing experiments. Ideally, we can extrapolate unfolding rate to a lower loading regime using an appropriate theoretical model, such as Bell-Evan, thus the computational and experimental results can become comparable.

Firstly, we applied Bell-Evan model $<F(\mathrm{r})> = (\mathrm{k_B}T/\Delta \mathrm{x})\ln(r^*\Delta \mathrm{x}/(k_0{}^*\mathrm{k_B}T)$ to make extrapolation from 5'-end translocation simulation, and obtained kinetic rates k_0 at zero force for barrier 1 and 2 (corresponding to peak 1 and 2) as 2.9×10^4 s^{-1} and 6.24×10^2 s^{-1}, respectively. The k_0 derived from our high loading rate using Bell-Evan formula is at least five orders of magnitude faster than that from experimental ones (smaller than 3.0×10^{-3} s^{-1}), which can be attributed to two reasons: 1) the assumption that the distance to the transition state, Δx, is independent on the applied force in the Bell-Evan model, doesn't hold for all loading rates or forces, as seen in many cases that applying data obtained from high loading regime (i.e MD simulation) to fit Bell-Evan model, generally leading to a significant underestimation of Δx and reaction time $1/k_0$[12, 13]; 2) the sequential transitions are generally not independent, the transition force is determined not only by the current barrier, but also the history of preceding barriers crossing, as reported by Dudko et al[14, 15].Therefore, it is not a trivial work to derive the free energy parameters as well as relevant kinetics about the barriers from force profiles. Recently, a sophisticated model has been developed by Zhang and Dudko to treat it, however, such model require a large data set[14, 15]. It is computationally expensive to handle the system with complex free energy landscape using all-atom MD simulation with explicit solvent model. It is still challenging to make accurate predictions about kinetics on order of second from all-atom MD simulation for a biological system.

In our work, we made compromises in our simulations to understand the mechanism of xrRNA1's anisotropic response to force loading. We found that the tertiary interaction network around 5'-end is highly coupled, it redistributes the forces into broad region upon tensions from the 5'-end, thus rendering its high 5'→3' direction mechanical stability; whereas the force exerted on the 3'-end is confined to break point or local segments, due to the absence of complex tertiary interactions at the 3'-end, thus lowering its mechanical resistance. Such coupling is expected to still work even at lower loading force under physiological or experimental conditions, conferring to the extraordinary mechanical anisotropy of ZIKV-xrRNA1 to resist the

degradation by Xrn1 from 5’→3’ direction and to be readily traversed by RdRP from 3’→5’ direction as a template for RNA synthesis.

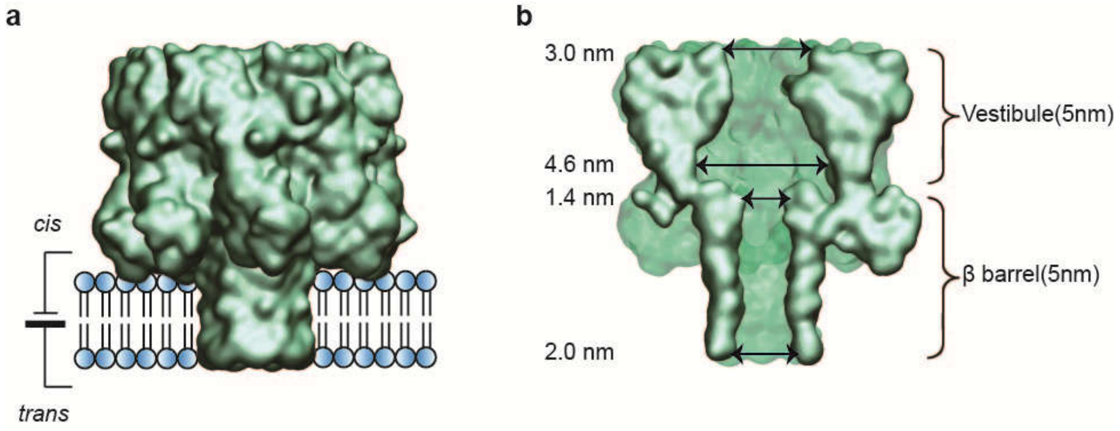

Figure S1. Dimensions of the α-hemolysin nanopore. (**a**) Space filling model showing the α-HL nanopore inserted in the phospholipid bilayer. (**b**) Cross section view and dimensions of the α-HL nanopore.

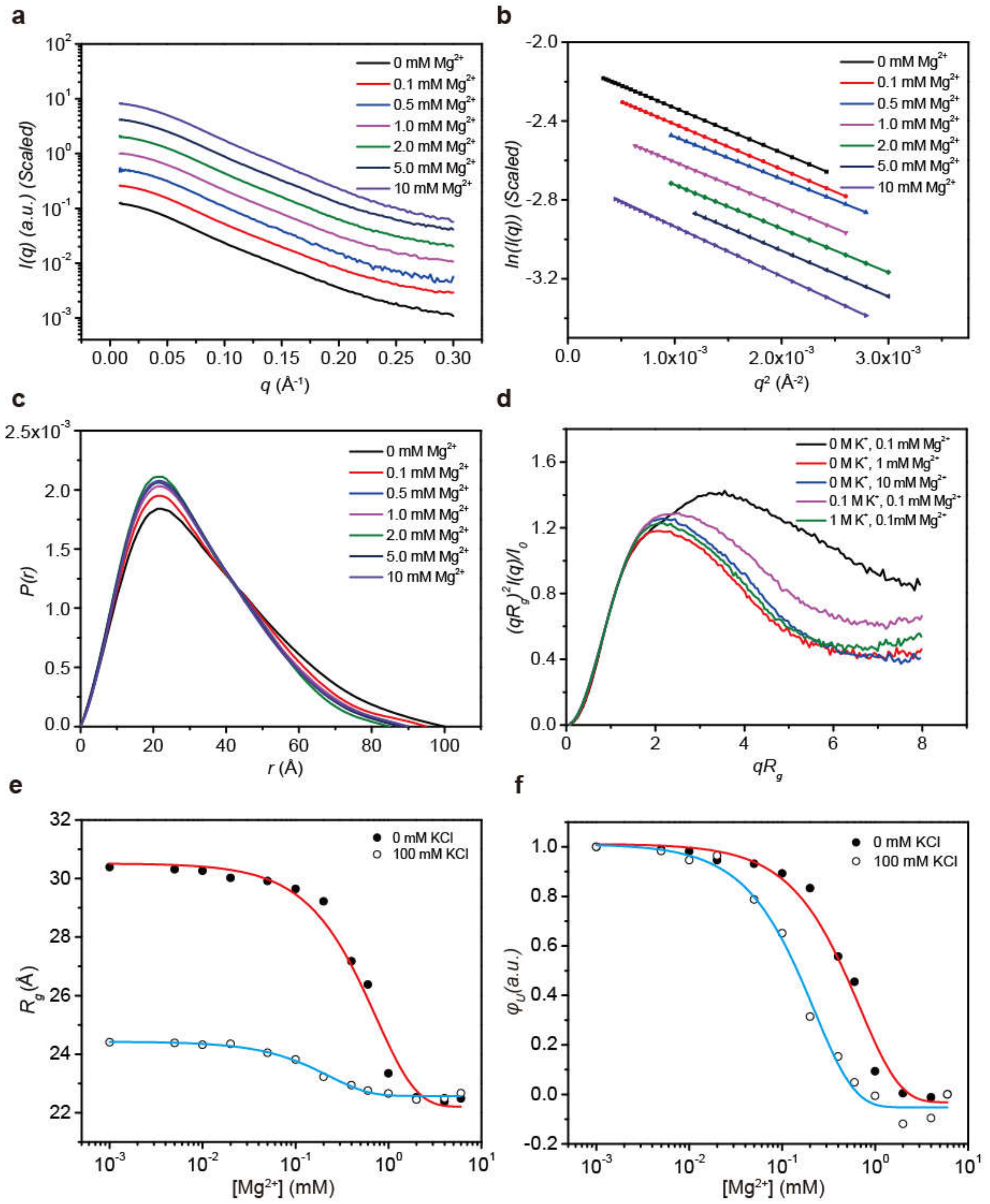

Figure S2. Mg^{2+}-dependent structural transition of ZIKV xrRNA1. (**a-d**) Experimental scattering profiles (**a**), guinier fitting (**b**), PDDFs (**c**) and dimensionless Kratky plots (**d**) for ZIKV-xrRNA1 in 20 mM Tris-HCl, pH 7.50 supplemented with different Mg^{2+} concentrations. (**e**) Plots of R_g as a function of $[Mg^{2+}]$ in buffers of 20 mM Tris-HCl (black solid circle), pH 7.50, or 20 mM Tris-HCl, 100 mM K^+, pH 7.50 (cyan open circle), where R_g was obtained from PDDF functions of SAXS data. (**f**) Fraction of unfolded RNA Φ_U versus $[Mg^{2+}]$. Symbols are the same as for (**e**). Source data for panel a-f are provided as a Source Data file.

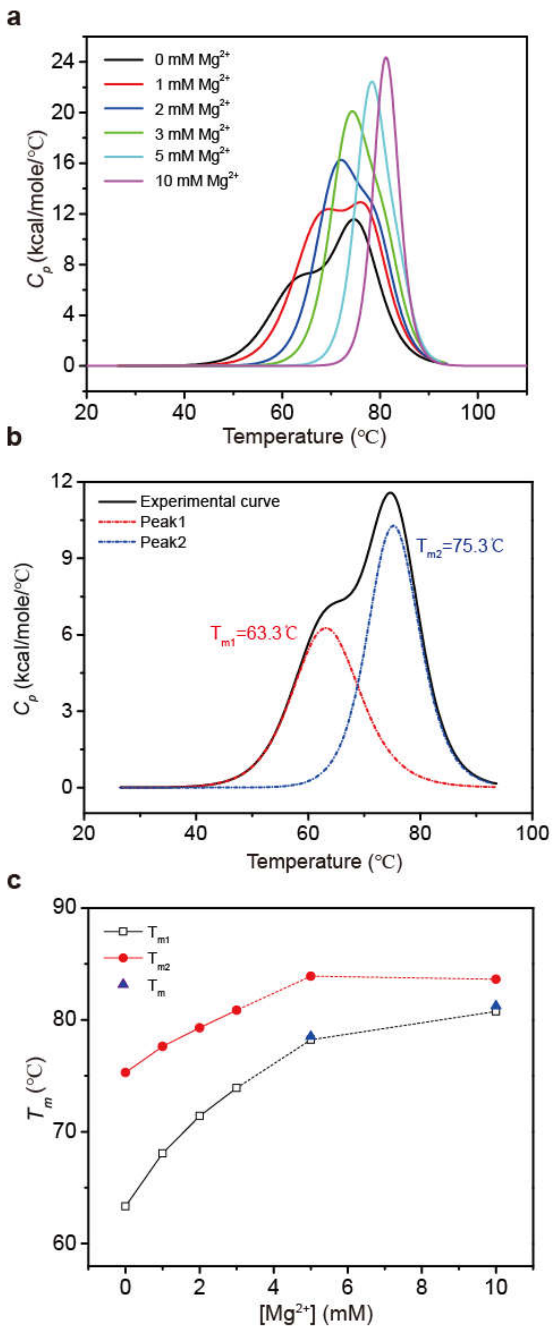

Figure S3. DSC analysis of the thermal stability of ZIKV-xrRNA1. (**a**) Thermal unfolding of ZIKV-xrRNA1 samples at a concentration of 30 μM and in buffers containing 10 mM sodium phosphate, 150 mM KCl, pH 7.5 and various concentrations of Mg^{2+}recorded by DSC. (**b**) A typical DSC thermogram for ZIKV-xrRNA1 in 0 mM Mg^{2+}. DSC thermograms at low Mg^{2+} concentrations (< 5mM) can be fitted into two distinct transition peaks with two T_m values T_{m1} and T_{m2}, corresponding to the unfolding of tertiary and secondary structure respectively, whereas with the increasing of Mg^{2+} concentrations, DSC thermograms for xrRNA1 can only be fitted into one peak with one transition temperature T_m, which xrRNA1 is considered to unfold cooperatively. (**c**) T_{m1}, T_{m2}andT_m values extracted from DSC were plotted against concentration of Mg^{2+}.

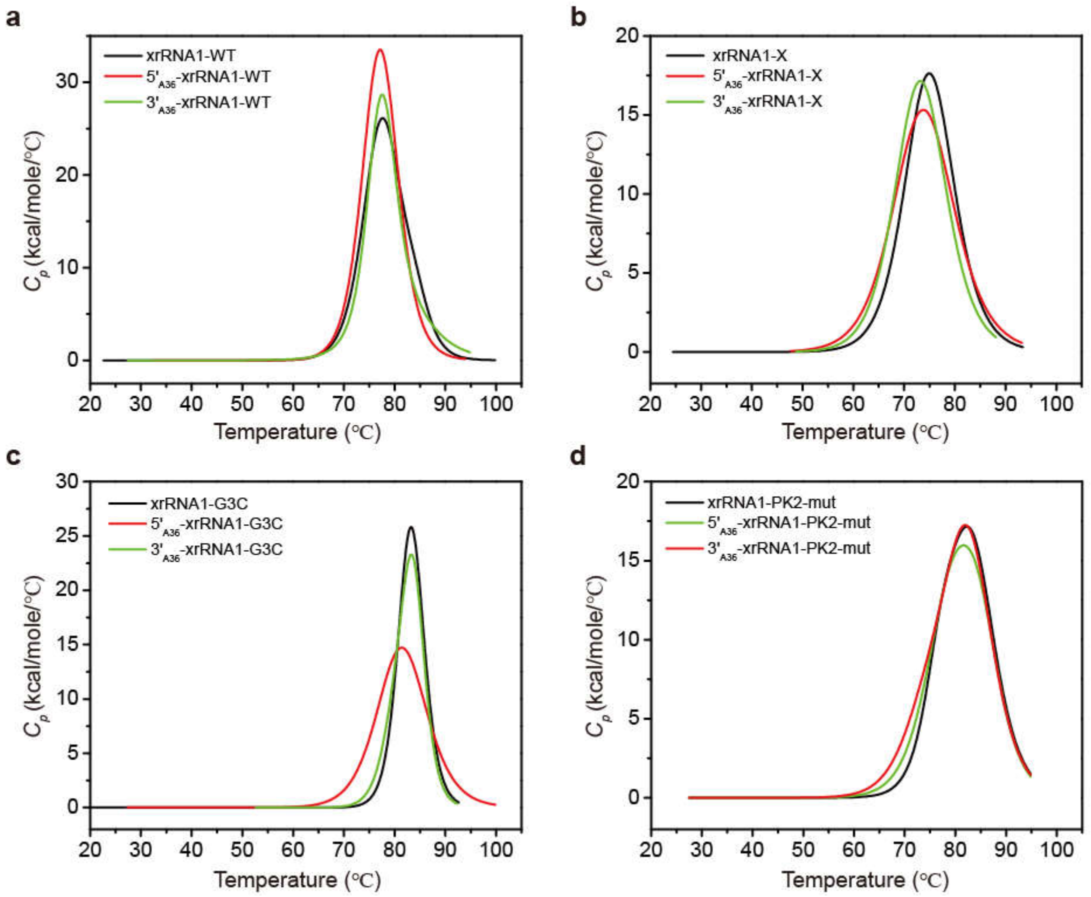

Figure S4. The effects of the 5' or 3' poly(rA) leader sequences on the thermal stability of ZIKV xrRNA1 and its mutants. (**a-d**) The DSC thermograms for ZIKV xrRNA1 (**a**), xrRNA1-X (**b**), xrRNA1-G3C (**c**) and xrRNA1-PK2mut (**d**) and their related constructs with 5' or 3' leader sequences show that the 5' or 3' leader sequences have minor effects on thermal stability of the corresponding RNAs. All the experiments were conducted in buffers containing 5 mM Mg^{2+}.

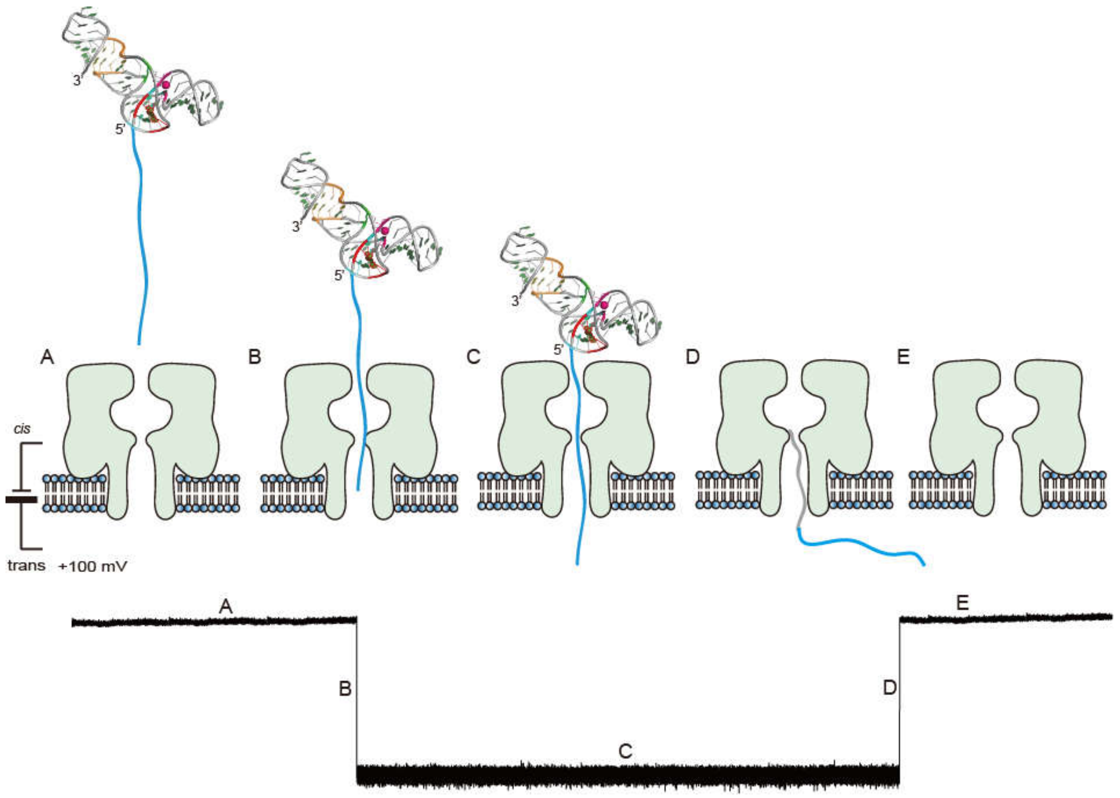

Figure S5. Schematic representationof xrRNA1 translocation and unfolding in the α-HL nanopore. A. open pore; B. leader sequence enters into the pore; C and D. RNA unfolds and then release; E. open pore. Due to the complexity of xrRNA1 structure, folding pathways and limitation of nanopore detection, it's difficult to have a thorough molecular interpretation of the blockade current traces but the duration time that RNA is trapped in the pore provides a measure of the RNAs' mechanical stability in a defined direction.

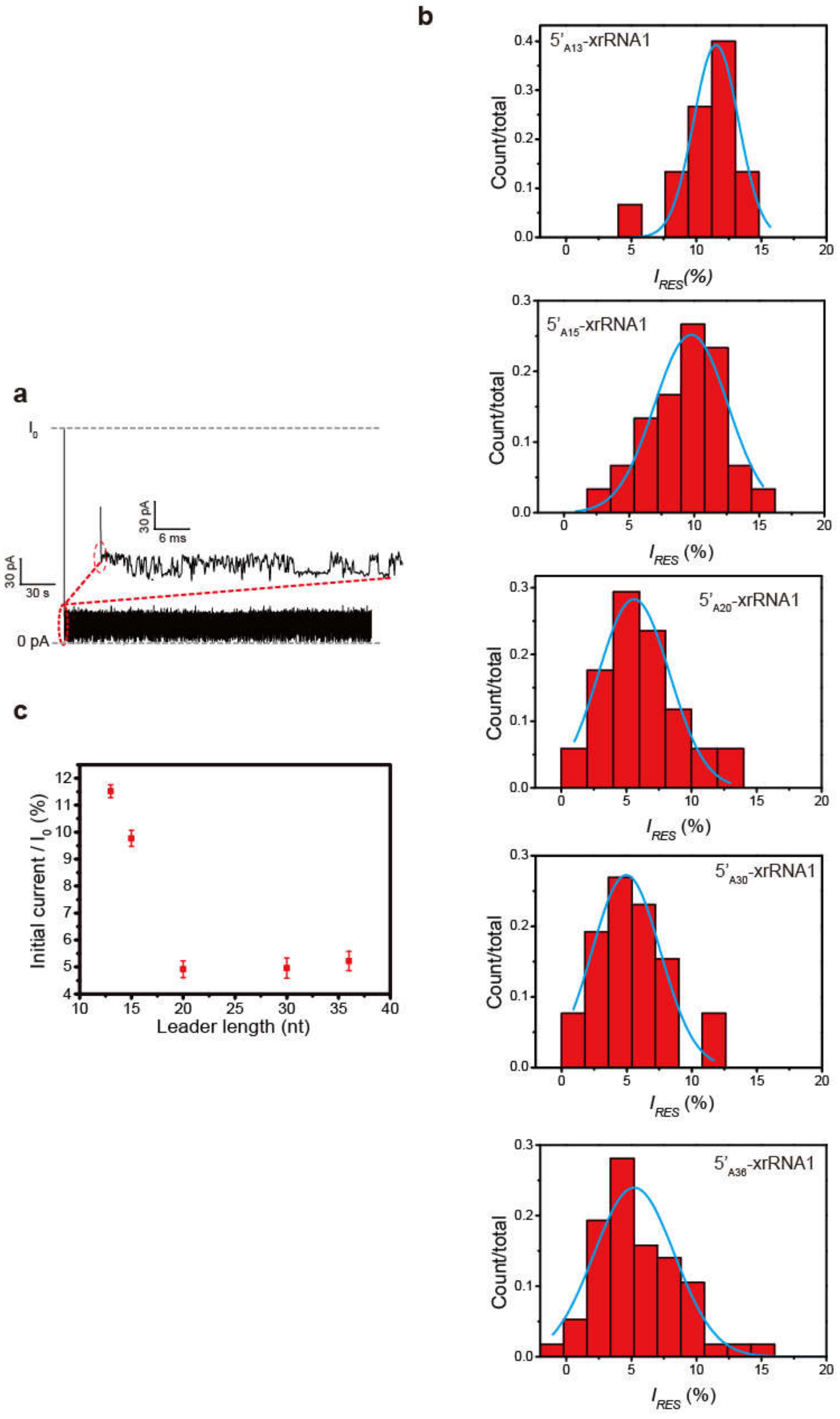

Figure S6. Initial current blockade signals generated by 5’-xrRNA1 with different poly(rA) leader sequences in length. (**a**) Representative current traces generated by 5’-xrRNA1s. Red circles denote the initial current blockade traces used for statistical analysis. (**b**) Initial current blockade histograms of 5’$_{A13}$-xrRNA1, 5’$_{A15}$-xrRNA1, 5’$_{A20}$-xrRNA1, 5’$_{A30}$-xrRNA1 and 5’$_{A36}$-xrRNA1. All experiments were conducted in buffers containing 20 mM Tris, pH 7.4, 1M (*cis*)/3M(*trans*) KCl supplemented with 5mM Mg^{2+} and at +100 mV. (**c**) Plots of the mean initial blockade current for 5’-xrRNA1 with different polyA leader sequences in length. The mean initial blockade currents were calculated from N > 50 events. Error bars represent the standard error of the mean. Source data for panel c is provided as a Source Data file.

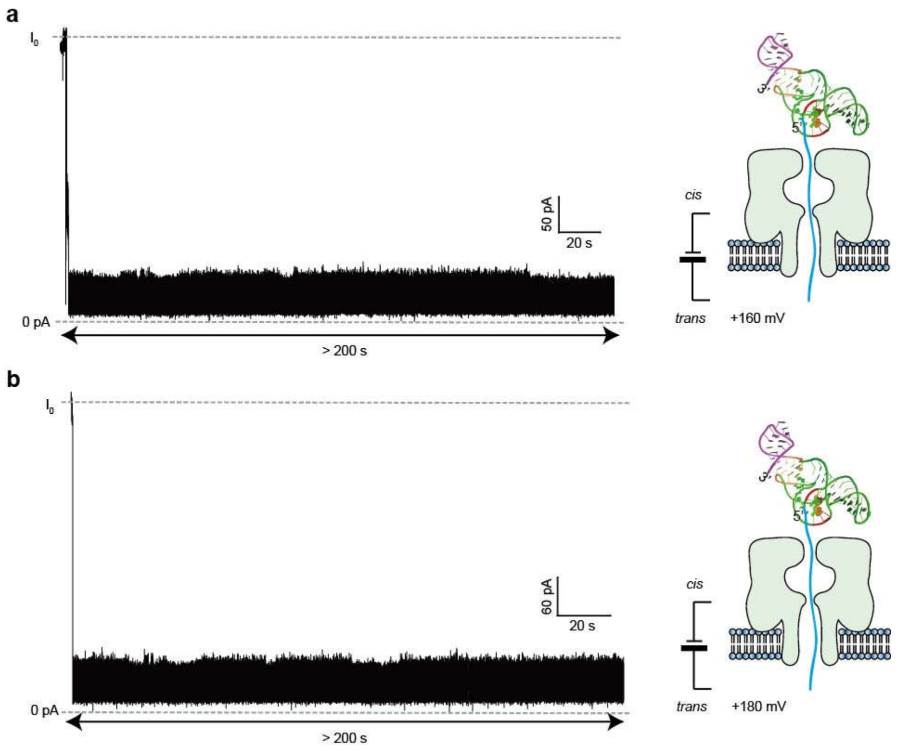

Figure S7. (**a**-**b**) Typical current blockade traces for 5'$_{A36}$-xrRNA1 in 5 mM Mg^{2+} in the α-HL nanopore under 160 mV (**a**) and 180 mV (**b**) transmembrane voltages.

a

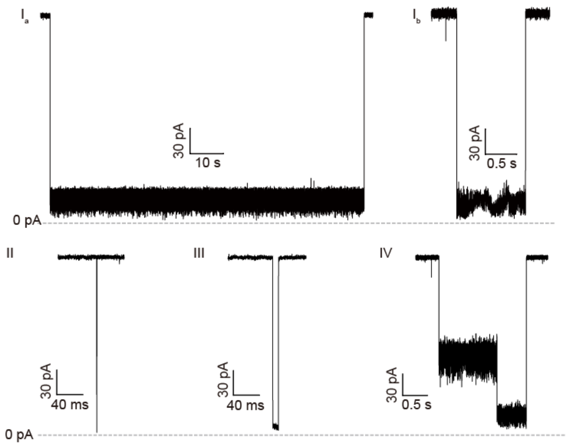

b

Type	3'$_{A36}$-xrRNA1	5'$_{A36}$-xrRNA1-X	3'$_{A36}$-xrRNA1-X	5'$_{A36}$-xrRNA1-G3C	3'$_{A36}$-xrRNA1-G3C	5'$_{A36}$-xrRNA1-PK2mut	3'$_{A36}$-xrRNA1-PK2mut	5'$_{A36}$-xrRNA1-X-ΔP4	3'$_{A36}$-xrRNA1-X-ΔP4
**I**	85%	70.91%	79%	1%	89%	75%	84%	71%	72%
**II**	5%	13.64%	5%	10%	2%	4%	6%	11%	5%
**III**	4%	11.82%	11%	51%	3%	17%	9%	18%	13%
**IV**	6%	3.64%	5%	38%	6%	4%	1%	0	10%

c

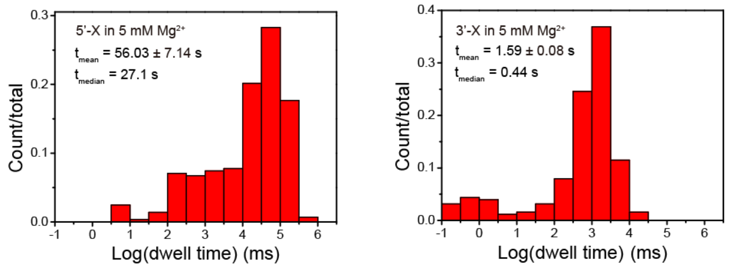

mean dwell time	5'X	3'X	median dwell time	5'X	3'X
type 1	60.78 ± 5.32 s	2.00 ± 0.15 s	type 1	35.81 s	1.40 s
all signal	56.03 ± 7.14 s	1.59 ± 0.08 s	all signal	27.10 s	0.44 s

Figure S8. Nanopore current blockade signatures for different xrRNA constructs in this study. (**a**) Typical current blockade signatures (types I, II, III and IV) generated by 5'$_{A36}$-xrRNA1-X and 3'$_{A36}$-xrRNA1-X in the presence of 5 mM Mg^{2+}. Type I represents the most typical events and is attributed to directional translocation guided by the leader sequence. Type II is characterized with mean duration time less than 1 ms, which could be the translocation signals of single-stranded RNAs due to the degradation or unfolding of xrRNA1. Type III typically show simple deep blockade current level (%I/I_0 = 10%) and short duration time (~0.16s), which should be the signals for partially folded xrRNA1. Unlike the other three types, type IV has two distinct sub-states. The ionic current initially drops to an intermediate current level of %I/I_0 = 40%, indicating that not the narrow constriction zone of the β-barrel but the cis vestibule of the pore is occupied at this stage, then is dominated by the deep blockade current level of %I/I_0 = 10% for long before re-establishing the open-pore current. Types II-IV do not feature the directional unfolding and translocation guided by the leader sequence. (**b**) Populations of different current blockade types generated by xrRNA1 and its mutants. (**c**) Dwell time distribution for 5'$_{A36}$-xrRNA1-X and 3'$_{A36}$-xrRNA1-X which the statistical analysis was based on all the signals collected instead of the type I only as in **Figure 3c**. The mean duration time for 5'$_{A36}$-xrRNA1-X is ~ 35 folds longer than that for 3'$_{A36}$-xrRNA1-X.

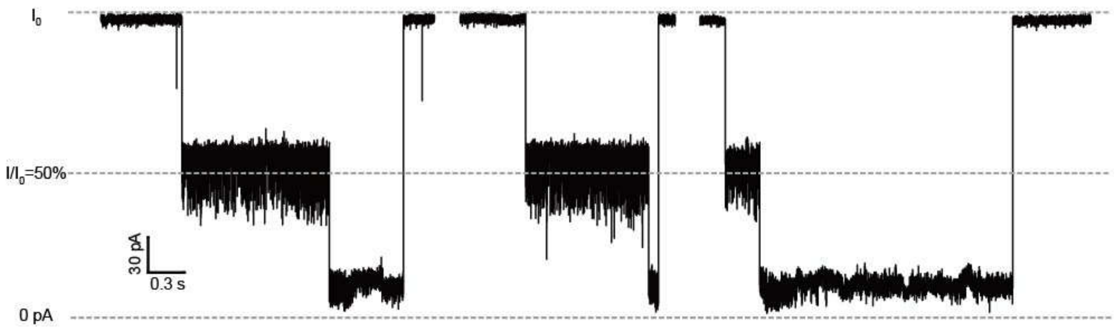

Figure S9. Nanopore current blockade signatures generated by ZIKV xrRNA1 without a leader sequence in buffer containing 5 mM Mg^{2+}. xrRNA1 generated a two-level blockade current pattern that the current firstly reduced to 50% of I_0, then reduced to a deep state with I/I_0= 10%. Previous study showed that DNA G-quadruplex is able to enter into the cis vestibule of the nanopore and cause blockade current level of I/I_0= 50%[16]. Given the structure of ZIKV xrRNA1 and the pattern of blockade current generated, it's likely that the L3-S4 pseudoknot (PK2) is conformationally dynamic or transiently unformed, the P4 helix at the 3' end of ZIKV xrRNA1 can be trapped in the vestibule of the nanopore first, then guide the unfolding and translocation of the remaining part of ZIKV xrRNA1 through the nanopore. Evidence for the dynamics of PK2 has been observed in the crystal structure of MVEV xrRNA2[17], which is highly homologous to ZIKV xrRNA1 but the PK2 is absent .

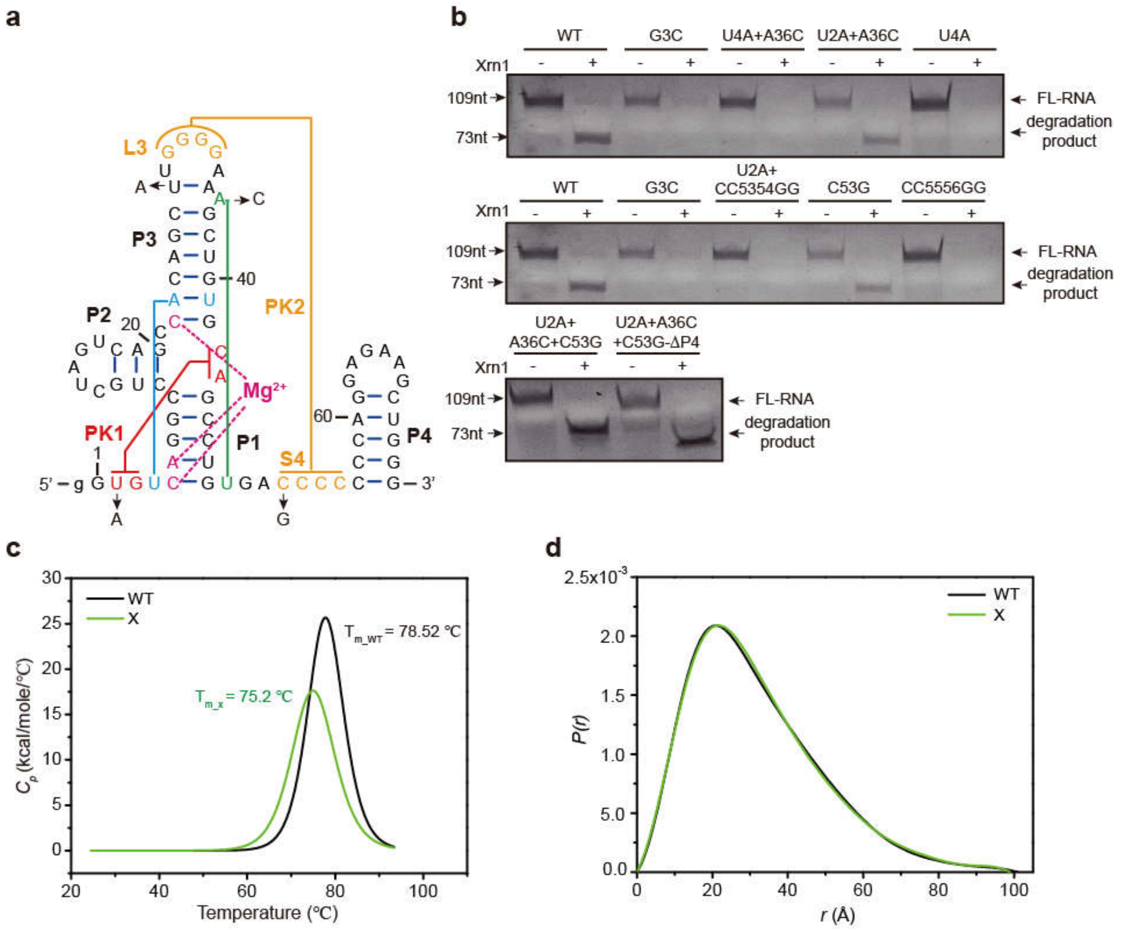

Figure S10. Screening a quadruple mutant xrRNA1-X to resemble xrRNA1. (**a**) Secondary structure of ZIKV xrRNA1, and the mutation site for a quadruple mutant xrRNA1-X are indicated with arrows. (**b**) Xrn1 resistance assay for ZIKV xrRNA1 and its various mutants. All the mutants were co-transcribed with a 36-nt poly(rA) leader sequence in the 5'end. (**c**) DSC thermograms for ZIKV xrRNA1 and xrRNA1-X in 5 mM Mg^{2+}. (**d**) The PDDF of xrRNA1-X can be nicely superimposed on that of ZIKV xrRNA1 in 5 mM Mg^{2+}. xrRNA1-X shows similar structure and thermal stability as ZIKV xrRNA1. Source data for panel **b** are provided as a Source Data File. Gels are representative of greeter than 3 independent experiments.

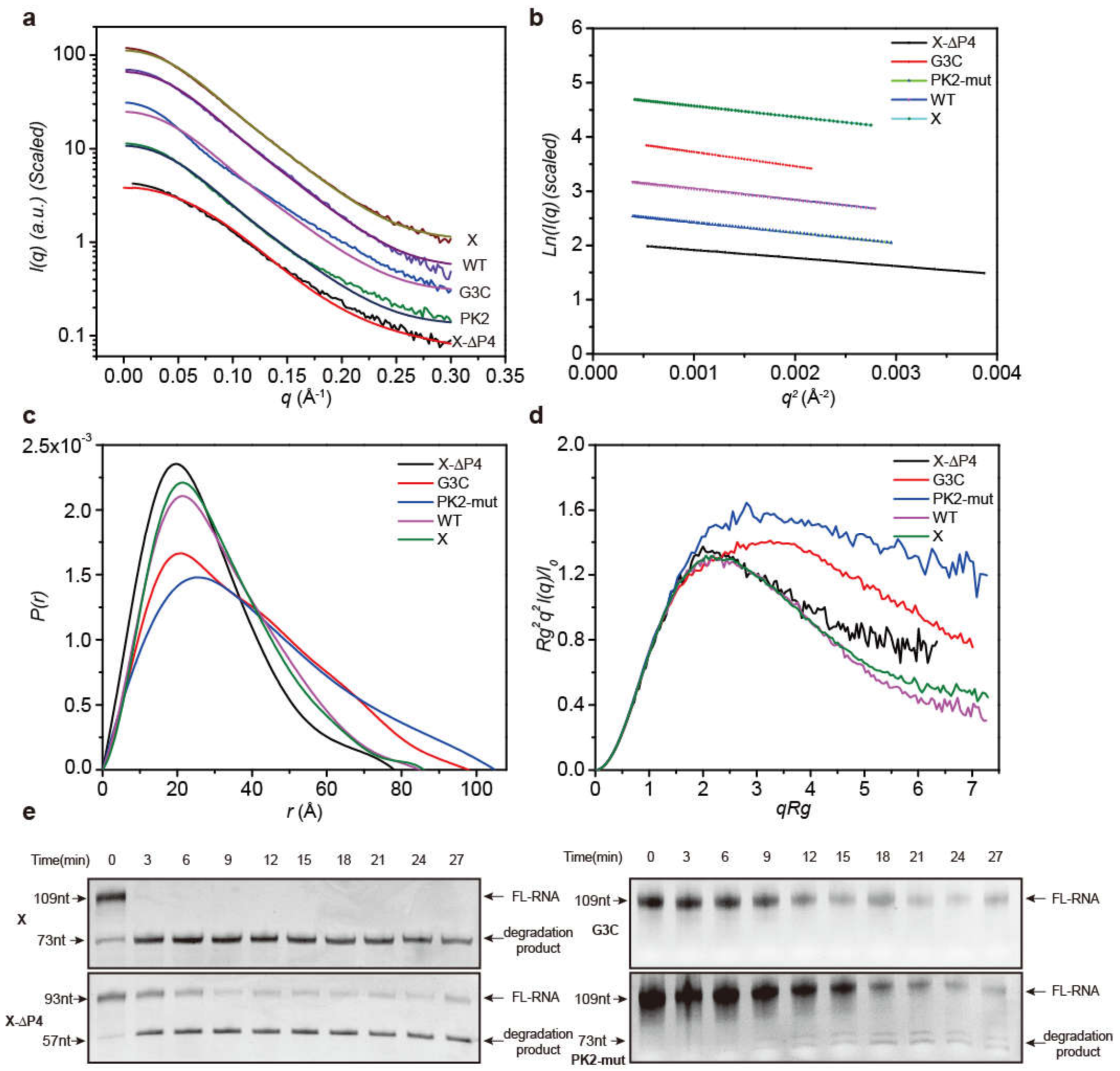

Figure S11. Loss of key tertiary interactions disrupts the structural integrity of ZIKV xrRNA1. Experimental scattering profiles (**a**), guinier fitting (**b**), PDDFs (**c**) and dimensionless kratky plots (**d**) for ZIKV xrRNA1 and its various mutants in 5 mM Mg^{2+}. In **a**, the theoretical scattering curve of ZIKV xrRNA1 crystal structure was fitted to the experimental scattering curves of xrRNA1, xrRNA1-X, xrRNA1-G3C, xrRNA1-PK2mut, respectively, the theoretical scattering curve of ZIKV xrRNA1 crystal structure without P4 was fitted to xrRNA1-X-ΔP4. (**e**) Xrn1 resistance assay for different ZIKV xrRNA1 mutants. Source data for panel **a-e** are provided as a Source Data File. Gels are representative of greeter than 3 independent experiments.

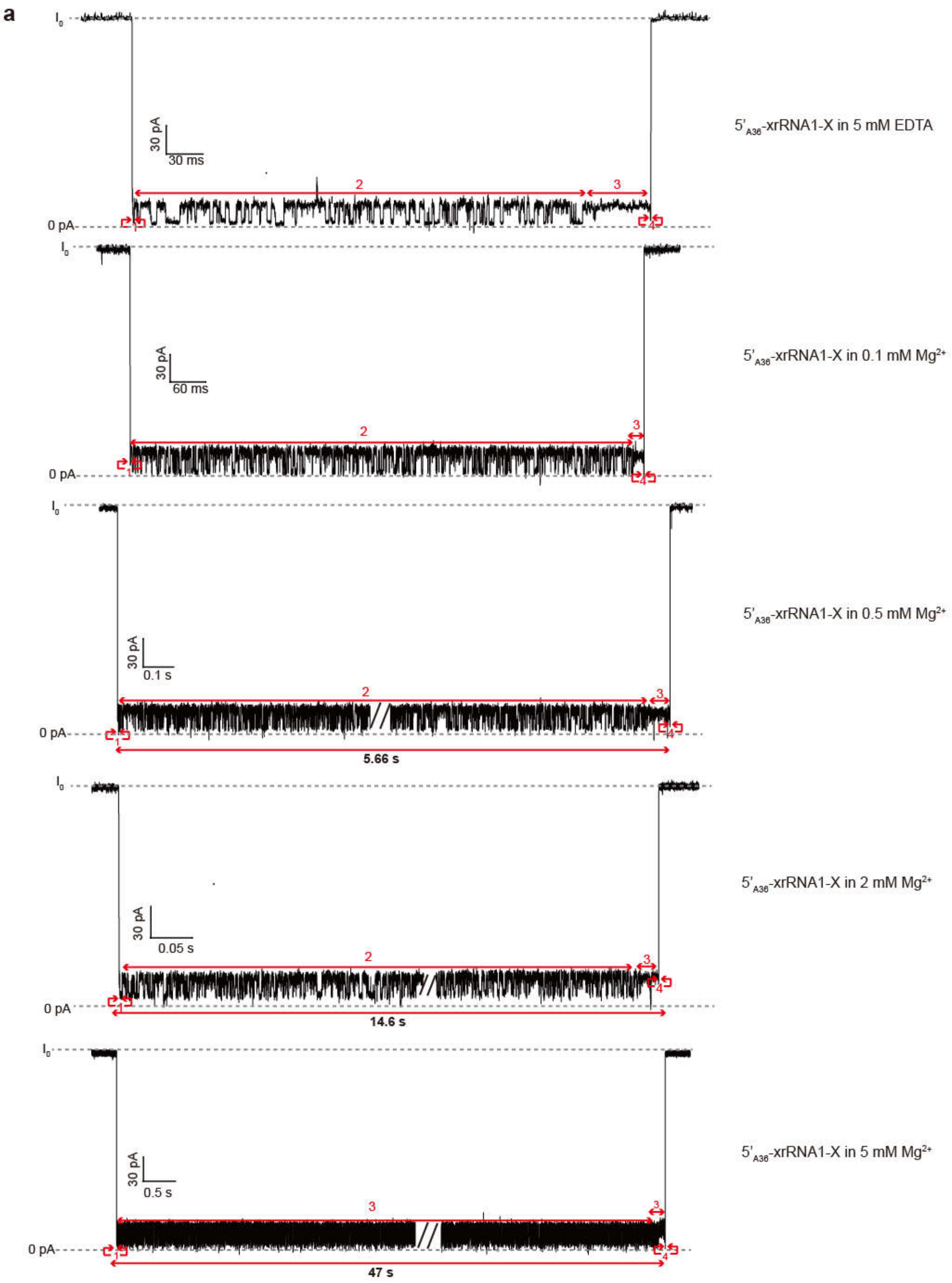

Figure S12. Representative current blockade traces generated by 5'$_{A36}$-xrRNA1-X in different Mg^{2+} concentrations. All the traces show similar patterns and we can roughly divide the unfolding process into four stages. Stage 1: leader sequence enters into the nanopore channel and pulls on the xrRNA1. Stage 2 and stage 3: pulling force leads to partial unfolding of xrRNA1. Stage 4: xrRNA1 unfolds completely and diffuses through the nanopore.

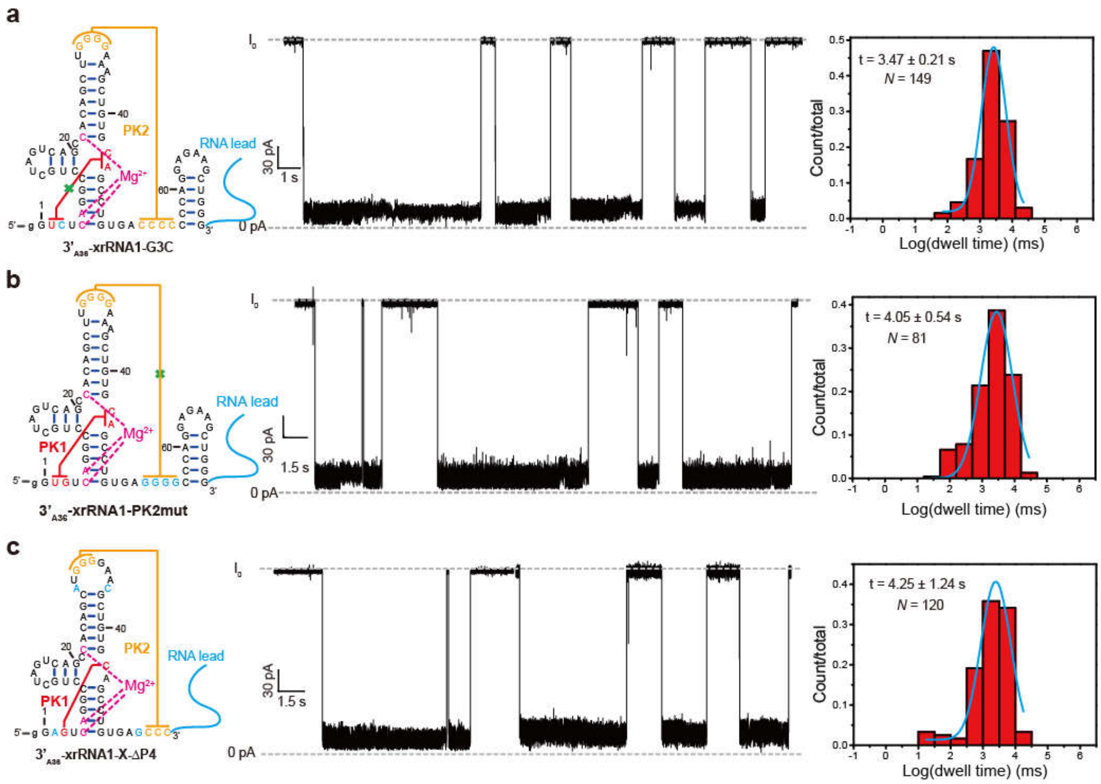

Figure S13. 3’→5’ directional unfolding of xrRNA1 mutants in the nanopore. (**a-c**) Secondary structure (**left**), representative current blockade traces (**middle**) and dwell time distribution histograms (**right**) for 3’$_{A36}$-xrRNA1-G3C (**a**), 3’$_{A36}$-xrRNA1-PK2mut (**b**) and 3’$_{A36}$-xrRNA1-X-ΔP4 (**c**).

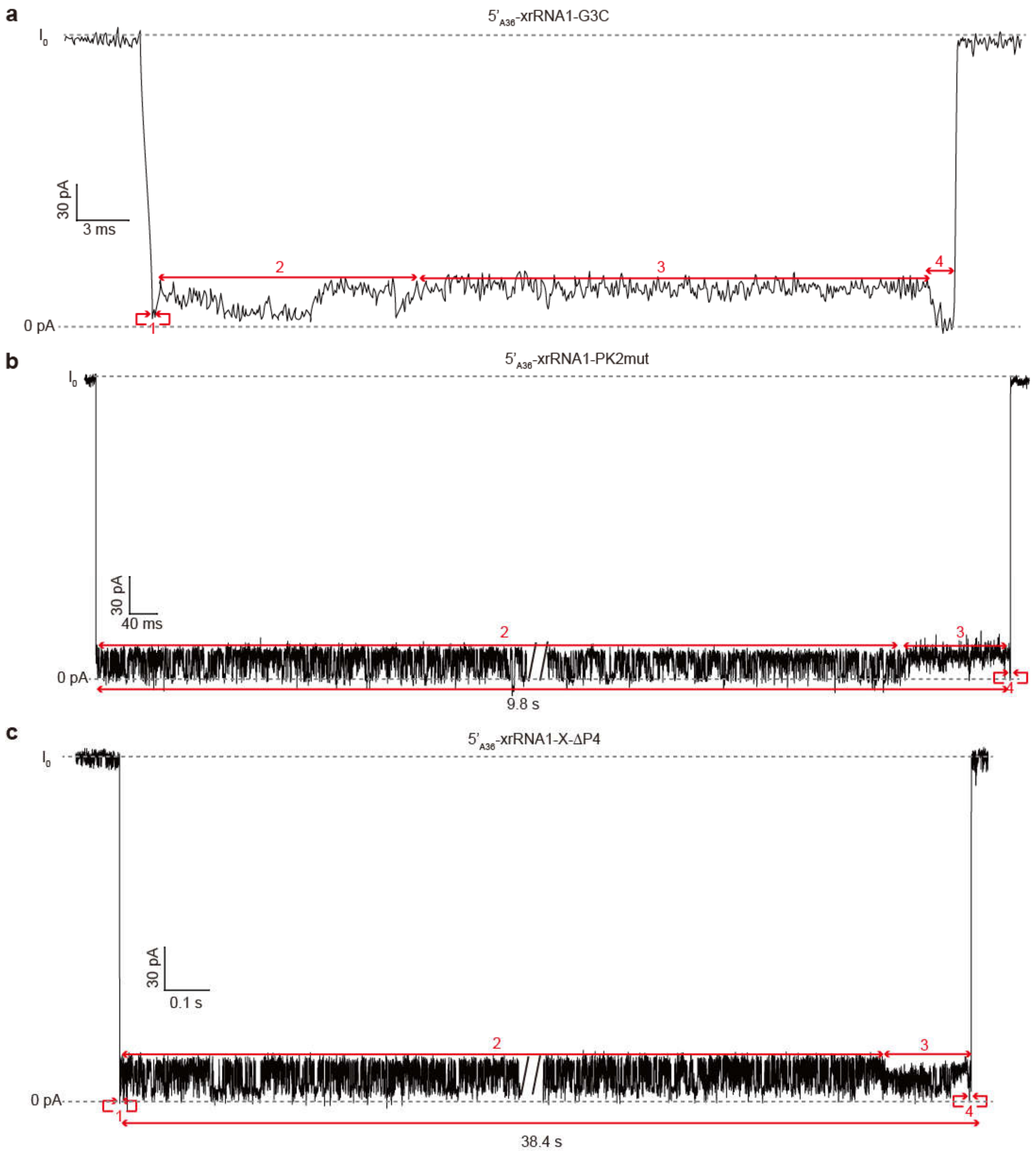

Figure S14. Representative current blockade traces generated by 5'$_{A36}$-xrRNA1-G3C (**a**), 5'$_{A36}$-xrRNA1-PK2mut (**b**) and 5'$_{A36}$-xrRNA1-ΔP4 (**c**).All the traces show similar patterns and the unfolding and translocation processes can be roughly divided into four stages.

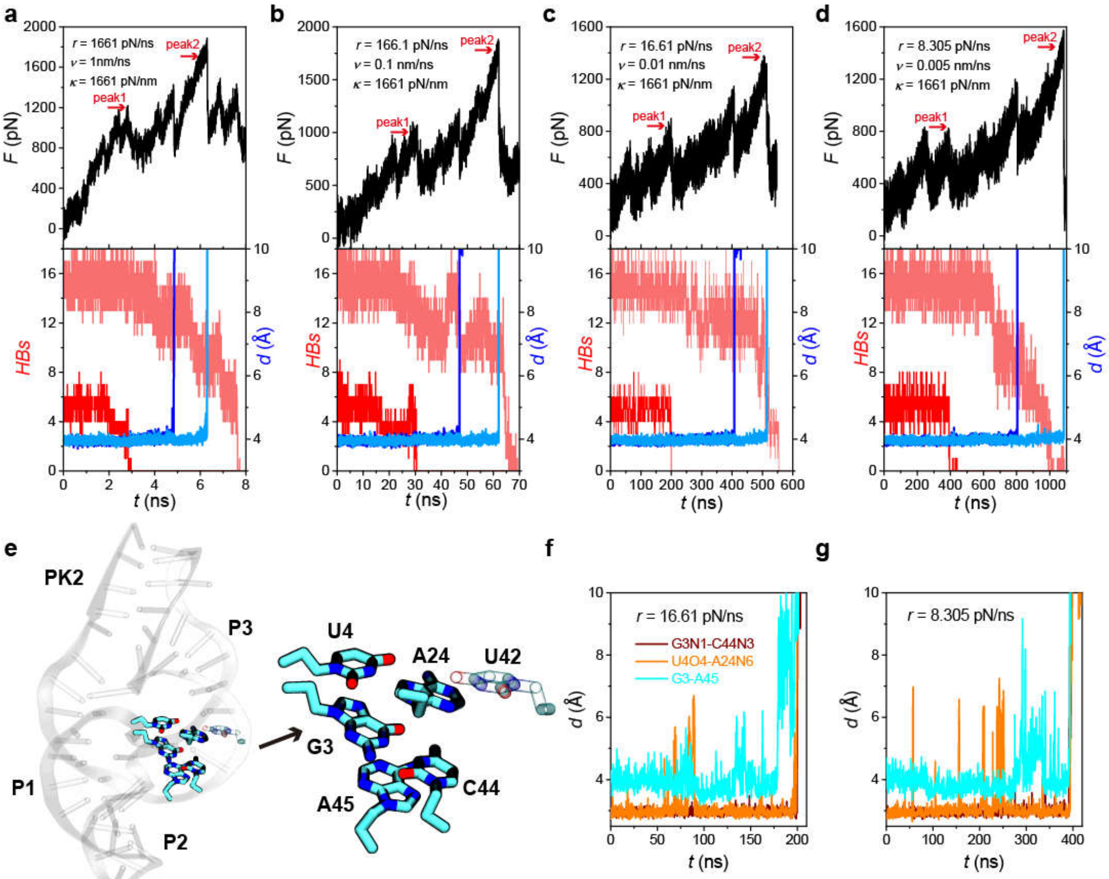

Figure S15.Mechanical unfolding ofZIKV xrRNA1 along the 5'→3' direction. (**a**-**d**) Representative unfolding trajectories at different loading rates (**a**: 1 nm/ns, **b**: 0.1 nm/ns, **c**: 0.01 nm/ns, **d**: 0.005 nm/ns), including force profiles (**top row**), hydrogen bondsinvolved in the 5'end structure (red: G3-C44 Watson-Crick pair in PK1 and U4·A24 Hoogsteen pair in U4·A24-U42 base triple) and stem P1 (pink), and sum of the distances between Mg^{2+} and two phosphate oxygen atoms (long range in sequence) which constitute twoMg^{2+}-clamps (blue: C5-Mg^{2+}-C23; skyblue: A6-Mg^{2+}-C23) during pulling(**bottom row**). (**e**) Stereoview of the 5'-end structure consisting of U4·A24 Hoogsteen pair and G3-C44 base pair, on which A45 stacks. (**f-g**)Detailed views of rupture of the 5'-end structure during pulling at lower loading rate of 16.61 (**f**) and 8.35 pN/ns (**g**), which are obtained from the same trajectories in (**c**) and (**d**), respectively. The base pairing is characterized as the distance of a representative atomic pair (e.g. G3N1-C44N3 for G3-C44, and U4O4-A24N6 for U4·A24), whereas the cross stacking between nucleobase of G3 and A45 is characterized by the distance among center of six-membered ring.

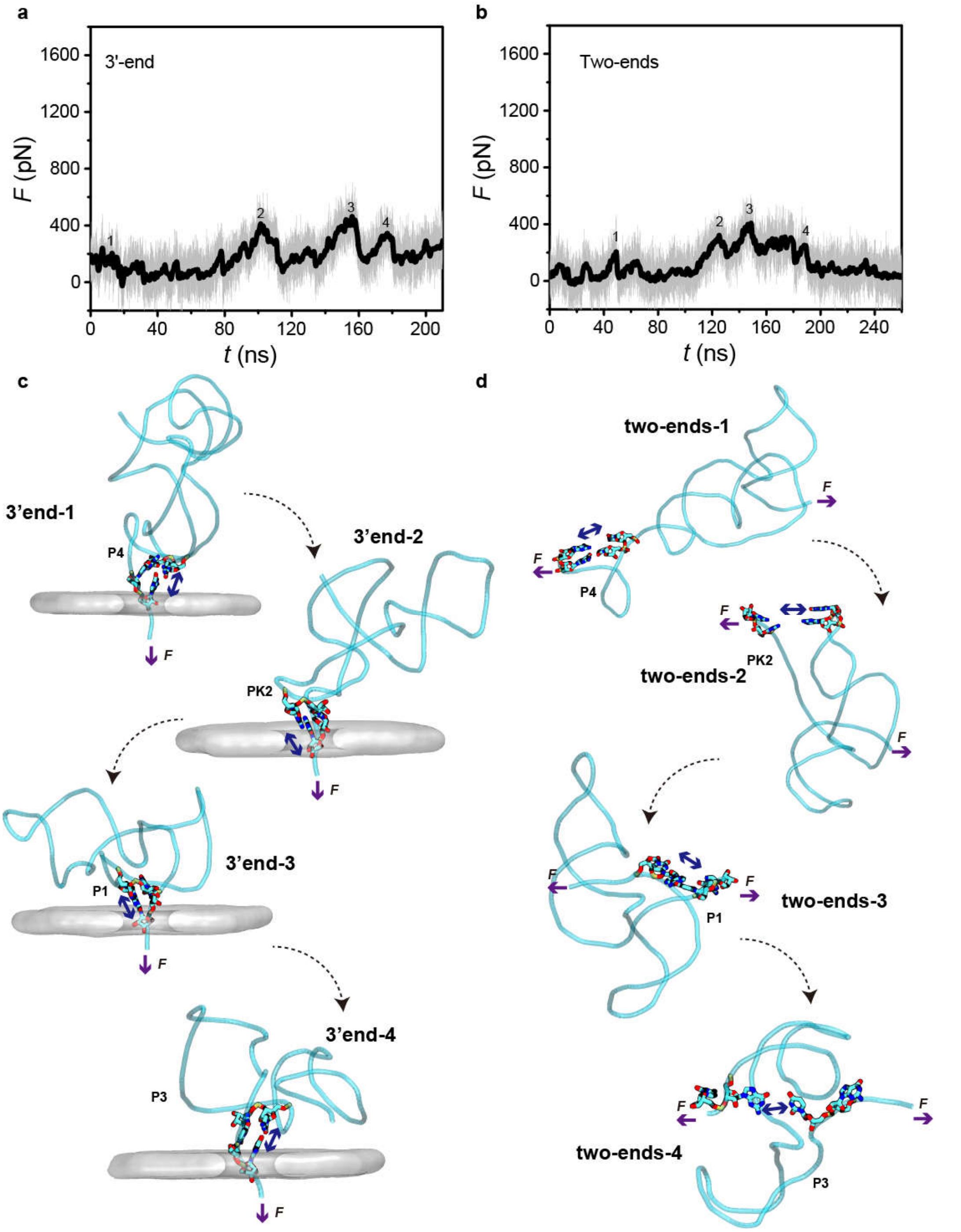

Figure S16.Mechanical unfolding ofZIKV xrRNA1 under 3'→5' (**a**) and two-end pulling directions (**b**) at loading rate of 166.1 pN/ns.The trajectoriesare the same as that in **Figure 6c** and **6d**, respectively in maintext. Each stem in ZIKV xrRNA1 is sequentially unfolded by loading forces.The snapshots corresponding to the numbered events on the respective force profiles (**a** and **b**)are shown in **c** and **d**, respectively. During pulling processes, the RNA translates and rotates, so that the base pairs to be broken (showed in bond) is nearly parallel to the pulling direction (**c** and **d**), lowering the forces required to unravel RNA through an unzipping mode.

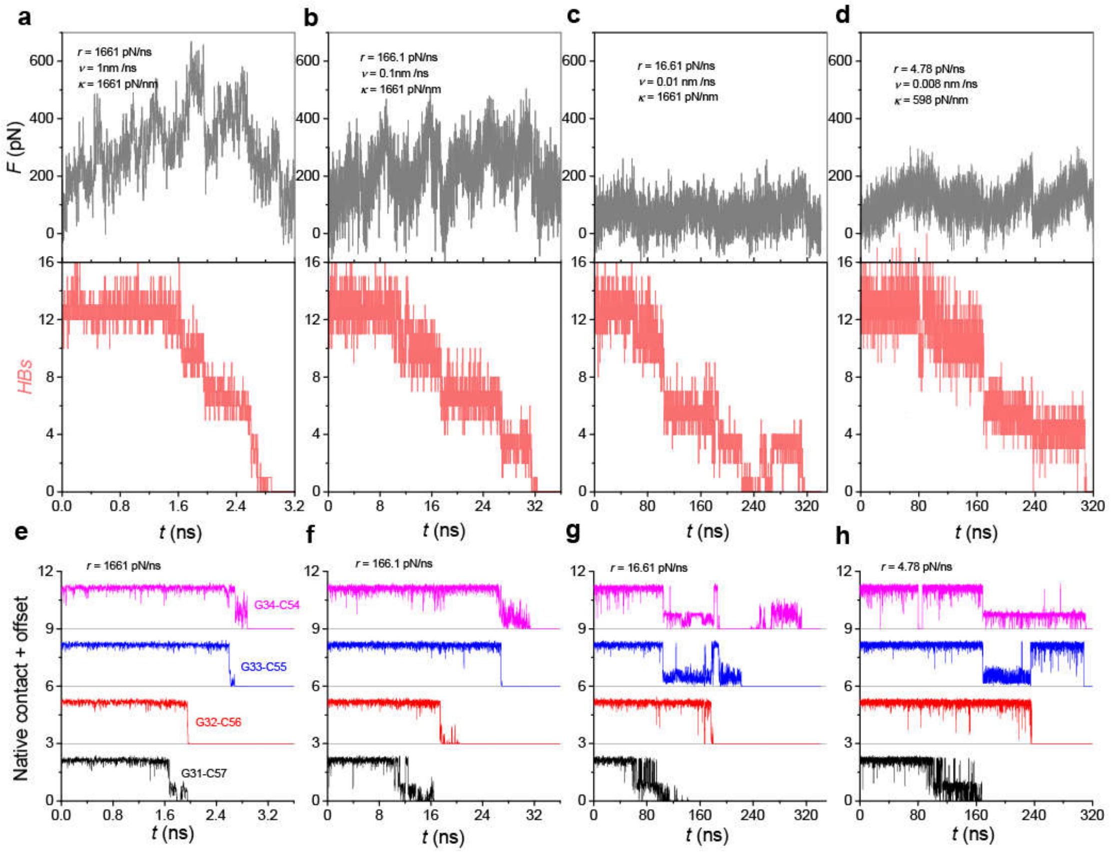

Figure S17.Representative mechanical unfolding trajectories of xrRNA1-ΔP4 along 3’ →5’ direction at different loading rates. (**a-d**) Force profiles (**top row**), hydrogen bonds of PK2 (**bottom row**) and(**e-h**) the native contact number (between the donor and acceptor in native hydrogen bonds) for each base pairing within PK2 (G34-C54, G33-C55, G32-C56, G31-C57) during pulling at different loading rates.

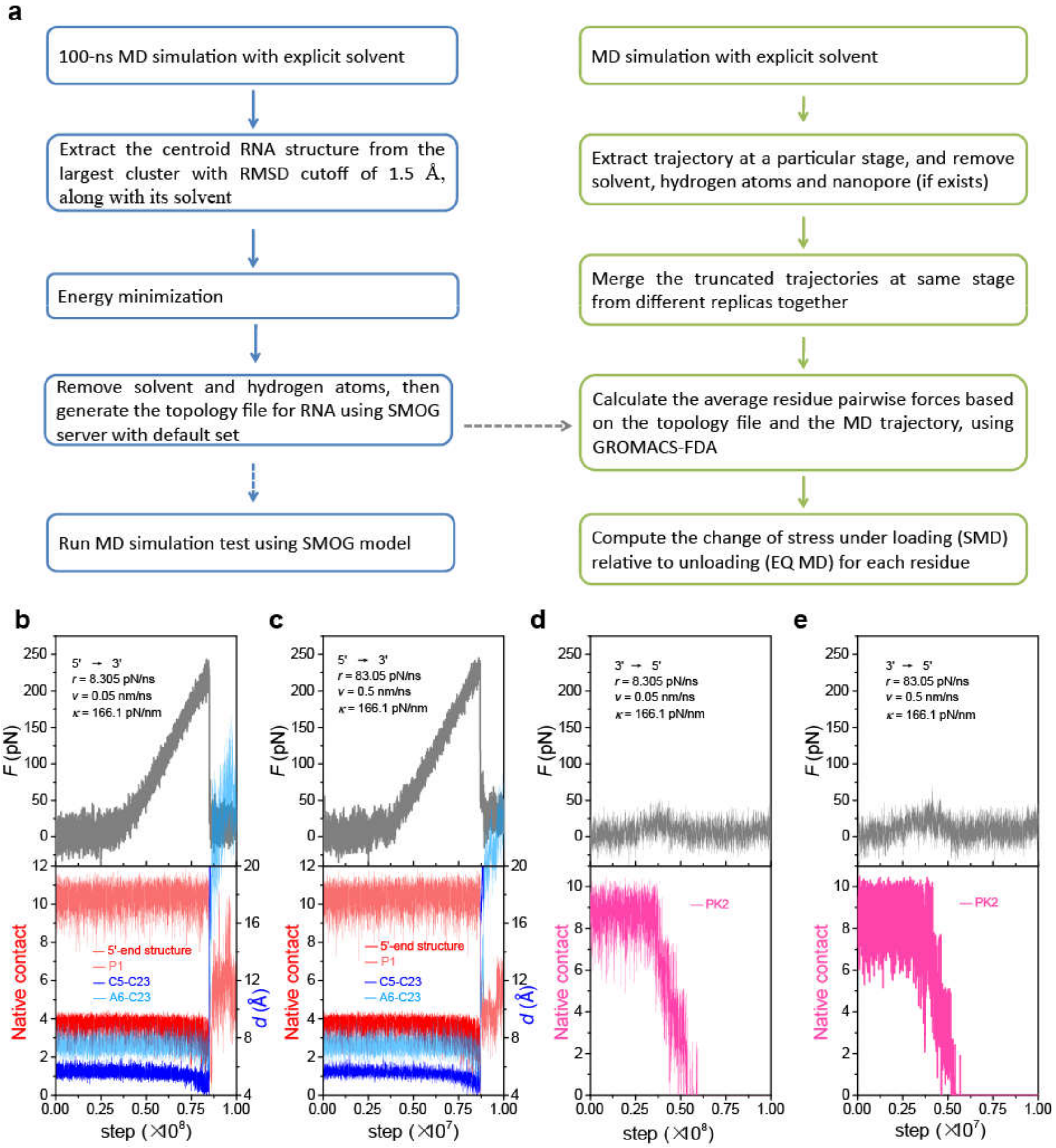

Figure 18. Computation using All-atom-structure-based model, SMOG. (**a**) Flowchart for force distribution analysis using GROMACS2018.7-FDA2.9 with SMOG model. Ensure that atoms listed in topology file (generated by SMOG server) are in the same order as the truncated trajectory. (**b-e**) Representative trajectories of SMD simulations with SMOG model for ZIKV xrRNA1-ΔP4 along 5'-end (**b-c**) and 3'-end (**d-e**) at different loading rates, including force profiles (**top row**), native contact (red: 5'-end structure; pink: stem P1; magenta: PK2) and distances between phosphorus atoms pairs (blue: C5-C23; skyblue: A6-C23) during pulling(**bottom row**). These phosphate moieties are rejoined by an Mg^{2+} ion in the crystal structure, formingtwoMg^{2+}-clamps (C5-Mg^{2+}-C23 and A6-Mg^{2+}-C23).

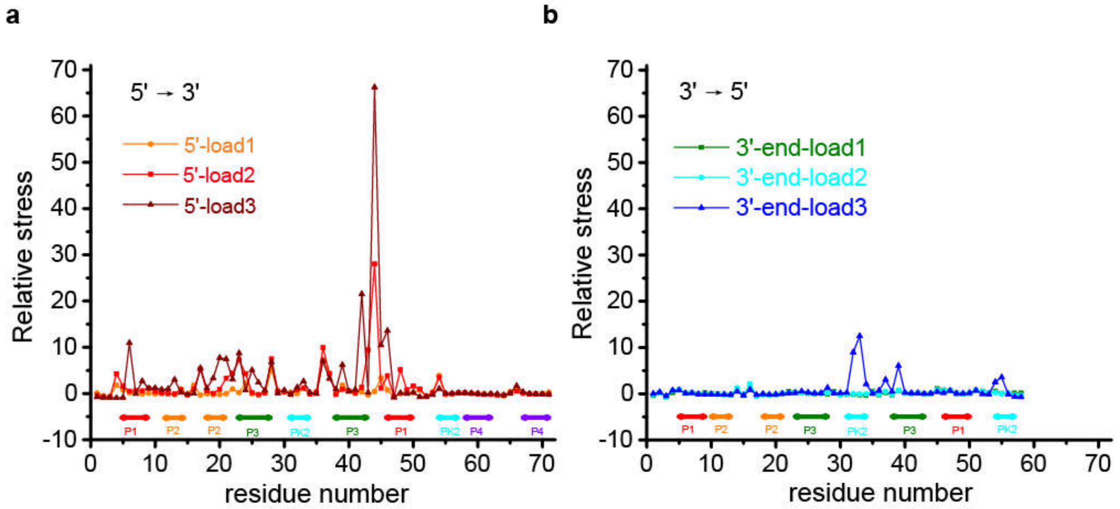

Figure S19. Force distribution analysis of ZIKV xrRNA1 at different stages of translocation from the 5'-end (**a**) or 3'-end (**b**). The different stages are defined in **Figure7a** and **b**, main text.

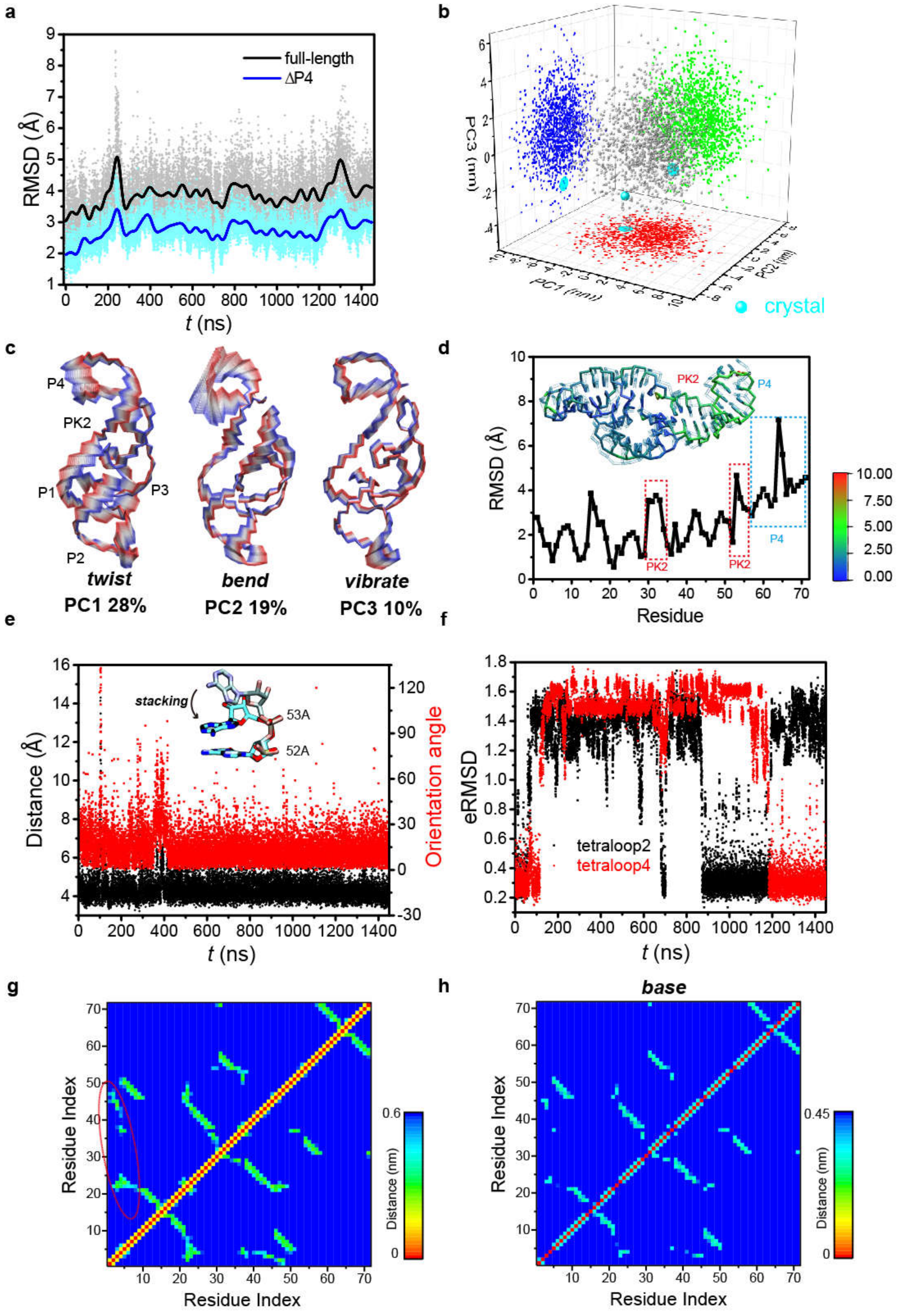

Figure S20.EquilibriumMD simulation up to 1450 ns for ZIKV xrRNA1. (**a**) All-heavy-atom-RMSD of full-length ZIKV xrRNA1 and its core structurexrRNA1-ΔP4 (without P4, ΔP4) with respect to the crystal structure. (**b**) The distribution of conformation ensemble (1 frame per ns) in the first three principal component eigenvectors from PCA analysis. (**c**) The first three major motions of xrRNA1

revealed by PCA analysis. (**d**) RMSD for each residue of xrRNA1 between the averaged structure derived from PCA analysis and the crystal structure. The inset shows superimposition of the averaged structure on the crystal structure, whichthe atoms (3 backbone atoms of P, C4’,C1’and 3 base atoms of N9,C4, N3 for purine or N1, N3, C5 for pyridine) used in PCA analysis are colored according to their deviation from crystal structure. (**e**) The distance (center of six-membered ring, black) and relative orientation (a vector normal to the base plane, red) between the A52 and A53 nucleobases in MD simulation, which characterizes the stacking of the two bases. The inset shows superimposition of the unstacked conformation in crystal lattice (light color) and the stacked conformation from MD simulations. (**f**) The eRMSD of the tetraloops 2 and 4 relative to the crystal structure during MD simulation. (**g-h**) Residue-residue contact map for xrRNA1 derived from second half of 1450-ns MD simulation (**upper triangle**) and crystal structure (**lower triangle**). The average minimum distances for each residue pairs were calculated with heavy atoms of nucleotide (**g**) or base (**h**) over a time window (725 ~ 1450 ns). The tertiary interactions involved in the 5’-end structure are highlighted by a red circle.

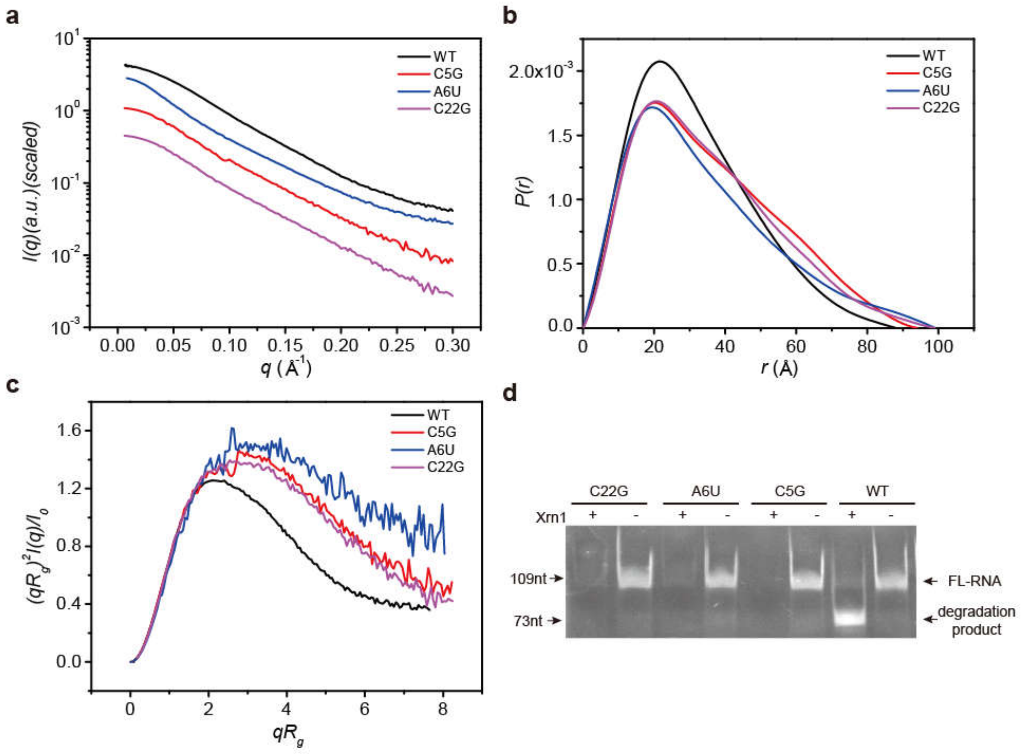

Figure S21. Mutants that disrupt Mg^{2+} binding site (C5G, A6U and C22G) cause improper folding of ZIKV xrRNA1 and loss of Xrn1 resistance ability. Experimental scattering profiles (**a**), PDDFs (**b**) and dimensionless Kratky plots (**c**) for ZIKV xrRNA1, xrRNA1-C5G, xrRNA1-A6U, and xrRNA1-C22G, respectively. (**d**) Xrn1 resistance assay for ZIKV xrRNA1 and its C5G, A6U, and C22G mutants. Source data for panel **a-d** are provided as a Source Data File. Gels are representative of greeter than 3 independent experiments.

Table S1. Basic structural parameters for ZIKV-xrRNA1 WT at various Mg^{2+} and K^{+} concentrations by SAXS.

Sample	[Mg^{2+}](mM)	[a]R_g(Å)	[b]R_g(Å)	D_{max} (Å)	[c]MW(kD)	[d]MW(kD)
[e]ZIKV-xrRNA1	0	30.42±1.19	32.65±0.33	110±2	22.44	27.62
	0.001	30.39±1.72	32.61±0.29	110±2		29.61
	0.005	30.31±1.20	32.53±0.28	108±2		29.56
	0.01	30.26±1.04	32.42±0.32	108±2		29.38
	0.02	30.02±1.41	32.34±0.29	107±2		29.38
	0.05	29.92±0.95	32.24±0.31	107±2		29.61
	0.1	29.64±0.85	31.90±0.35	106±2		28.78
	0.2	29.22±0.86	31.53±0.34	105±2		28.65
	0.4	27.18±0.77	29.02±0.27	98±2		27.51
	0.6	26.38±0.58	28.08±0.27	97±2		27.13
	1.0	23.34±0.63	24.40±0.25	86±2		25.84
	2.0	22.53±0.51	23.32±0.20	80±2		24.93
	4.0	22.38±0.58	23.17±0.19	79±2		24.79
	6.0	22.49±0.93	23.28±0.21	81±2		25.07
	10	24.40±0.46	25.57±0.37	100±2		26.48
[f]ZIKV-xrRNA1	0	24.45±0.88	25.71±0.37	95±2		22.72
	0.001	24.41±0.84	25.64±0.35	94±2		22.82
	0.005	24.38±0.77	25.58±0.37	94±2		23.20
	0.01	24.32±0.64	25.60±0.35	94±2		22.71
	0.02	24.35±0.76	25.45±0.40	94±2		22.64
	0.05	24.05±0.51	25.38±0.28	92±2		22.73
	0.1	23.82±0.65	24.95±0.29	91±2		22.63
	0.2	23.23±0.62	24.27±0.30	88±2		22.59
	0.4	22.94±0.52	23.95±0.26	85±2		22.45
	0.6	22.76±0.48	23.74±0.22	82±2		22.46
	1	22.66±0.61	23.46±0.20	80±2		22.39
	2	22.45±0.44	23.24±0.22	78±2		22.29
	4	22.49±0.61	23.29±0.23	79±2		22.40
	6	22.67±0.60	23.49±0.25	82±2		22.48
	10	22.77±0.74	23.59±0.25	83±2		22.98
[g]ZIKV-xrRNA1	0.1	23.47±0.30	24.16±0.27	90±2		23.91
	0.5	23.25±0.48	24.00±0.27	89±2		23.90
	1.0	23.32±0.36	24.02±0.25	89±2		24.88
	2.0	23.40±0.30	24.02±0.23	89±2		24.76
	5.0	23.49±0.35	24.18±0.26	91±2		25.02
	10	24.37±0.27	25.30±0.33	97±2		25.04

[a]derived from Guinier fitting;
[b]derived from GNOM analysis;
[c]MW: molecular weight predicted from sequences;
[d]MW: molecular weight calculated based on the power law of volume of correlation;
[e]Buffer: 20 mM Tris-HCl, pH 7.50;
[f]Buffer: 20 mM Tris-HCl, 100 mM KCl, pH 7.50;
[g]Buffer: 20 mM Tris-HCl, 1000 mM KCl, pH 7.50.

Table S2. Transition temperatures for ZIKV-xrRNA1 and its mutants at various buffer conditions by DSC.

Sample	$[Mg^{2+}]$(mM)	$^{a}T_{m1}$(°C)	$^{b}T_{m2}$(°C)	$^{c}T_{m}$(°C)
ZIKV-xrRNA1	0	63.33	75.30	-
	1	68.07	77.63	-
	2	71.41	79.29	-
	3	73.92	80.86	-
	5	-	-	78.52
	10	-	-	81.25
$5'_{A36}$-xrRNA1	5	-	-	77.21
$3'_{A36}$-xrRNA1		-	-	77.46
xrRNA1-X		-	-	75.08
$5'_{A36}$-xrRNA1-X		-	-	73.97
$3'_{A36}$-xrRNA1-X		-	-	73.34
xrRNA1-G3C		-	83.22	-
$5'_{A36}$-xrRNA1-G3C		-	81.52	-
$3'_{A36}$-xrRNA1-G3C		-	82.98	-
xrRNA1-PK2-mut		77.28	83.25	-
$5'_{A36}$-xrRNA1-PK2mut		79.15	84.66	-
$3'_{A36}$-xrRNA1-PK2mut		74.98	82.60	-

[a]Transition temperature derived from the 1st peak, which corresponds to the unfolding of tertiary structures;

[b]Transition temperature derived from the 2nd peak, which corresponds to the unfolding of secondary structures;

[c]Transition temperature corresponds to the cooperative unfolding of xrRNA1.

Table S3. Basic structural parameters for ZIKV-xrRNA1 and its mutants at 5 mM Mg^{2+} concentrations by SAXS.

[a]**Sample**	[b]$\boldsymbol{R_g}$**(Å)**	[c]$\boldsymbol{R_g}$**(Å)**	$\boldsymbol{D_{max}}$ **(Å)**	[d]**MW(kD)**	[e]**MW (kD)**
xrRNA1-WT	22.49±0.61	23.29±0.23	85±2	22.44	22.40
xrRNA1-X	24.61±0.14	25.50±0.11	86±2		25.48
xrRNA1-G3C	28.05±0.21	29.53±0.18	98±2		26.57
xrRNA1-PK2mut	31.92±1.88	32.10±0.20	105±2		24.22
xrRNA1-X-ΔP4	21.11±1.15	23.22±2.00	78±2	17.43	17.53
xrRNA1-C5G	27.94±0.67	28.39±0.41	94±2	22.44	26.99
xrRNA1-A6U	26.97±0.88	27.45±1.76	99±2		28.42
xrRNA1-C22G	27.61±0.49	27.88±0.43	99±2		23.26

[a]Buffer: 20 mM Tris-HCl, 100 mM KCl, 5 mM $MgCl_2$, pH 7.50;
[b]derived from Guinier fitting;
[c]derived from GNOM analysis;
[d]MW: molecular weight predicted from sequences;
[e]MW: molecular weight calculated based on the power law of volume of correlation.

Table S4. The Dwell time of directional translocation through the nanopore for ZIKV xrRNA1 and its mutant constructs.

Construct	Buffer condition	Translocation direction	Dwell time (s)
xrRNA1-WT	5 mM Mg^{2+}	5' → 3'	> 300
		3' → 5'	2.06 ± 0.58
xrRNA1-X	5 mM Mg^{2+}	5' → 3'	60.78 ± 5.32
		3' → 5'	2.01 ± 0.15
	5 mM EDTA	5' → 3'	0.70 ± 0.02
		3' → 5'	1.21 ± 0.07
xrRNA1-G3C	5 mM Mg^{2+}	5' → 3'	0.09 ± 0.04
		3' → 5'	3.47 ± 0.21
xrRNA1-PK2mut	5 mM Mg^{2+}	5' → 3'	5.49 ± 1.74
		3' → 5'	4.05 ± 0.54
xrRNA1-X-ΔP4	5 mM Mg^{2+}	5' → 3'	46.77 ± 8.81
		3' → 5'	4.25 ± 1.24

Table S5.a list of trajectory numberfor 3'-end pulling simulations on xrRNA1-Δ P4

Index	Loading rate (pN/nm)	Trajectory number
1	4.784	9
2	16.61	8
3	166.1	16
4	1661	20

Table S6. The primary sequences of ZIKV xrRNA1 and its mutant constructs used in this study.

Construct	RNA sequences
5'$_{A36}$-xrRNA1-WT	5'-(A)$_{36}$GGUGUCAGGCCUGCUAGUCAGCCACAGCUUGGGGAA<br>AGCUGUGCAGCCUGUGACCCCCCCAGGAGAAGCUGGG-3'
3'$_{A36}$-xrRNA1-WT	5'-GGUGUCAGGCCUGCUAGUCAGCCACAGCUUGGGGAAAGC<br>UGUGCAGCCUGUGACCCCCCCAGGAGAAGCUGGG-(A)$_{36}$-3'
5'$_{A36}$-xrRNA1-X	5'-(A)$_{36}$GGAGUCAGGCCUGCUAGUCAGCCACAGCAUGGGGAA<br>CGCUGUCAGCCUGUGAGCCCCCCAGGAGAAGCUGGG-3'
3'$_{A36}$-xrRNA1-X	5'-GGAGUCAGGCCUGCUAGUCAGCCACAGCAUGGGGAACGC<br>UGUGCAGCCUGUGAGCCCCCCAGGAGAAGCUGGG-(A)$_{36}$-3'
5'$_{A36}$-xrRNA1-G3C	5'-(A)$_{36}$-GGUCUCAGGCCUGCUAGUCAGCCACAGCUUGGGGAA<br>AGCUGUGCAGCCUGUGACCCCCCCAGGAGAAGCUGGG-3'
3'$_{A36}$-xrRNA1-G3C	5'-GGUCUCAGGCCUGCUAGUCAGCCACAGCUUGGGGAAAGCU<br>GUGCAGCCUGUGACCCCCCCAGGAGAAGCUGGG-(A)$_{36}$-3'
5'$_{A36}$-xrRNA1-PK2-mut	5'-(A)$_{36}$GGUGUCAGGCCUGCUAGUCAGCCACAGCUUGGGGA<br>AAGCUGUGCAGCCUGUGAGGGGCCCAGGAGAAGCUGGG-3'
3'$_{A36}$-xrRNA1-PK2-mut	5'-GGUGUCAGGCCUGCUAGUCAGCCACAGCUUGGGGAAAGCU<br>GUGCAGCCUGUGAGGGGCCCAGGAGAAGCUGGG-(A)$_{36}$-3'
5'$_{A36}$-xrRNA1-X-ΔP4	5'-(A)$_{36}$-GGAGUCAGGCCUGCUAGUCAGCCACAGCAUGGGGAA<br>CGCUGUGCAGCCUGUGAGCCC-3'
3'$_{A36}$-xrRNA1-X-ΔP4	5'-GGAGUCAGGCCUGCUAGUCAGCCACAGCAUGGGGAACG<br>CUGUGCAGCCUGUGAGCCC-(A)$_{36}$-3'

Table S7. The primer sequence used in this study.

Primer	Sequences
Forward primer	5'GTAACCCACTCGTGCACCCAACTGATCTTC3'
5’WT-R	5’CCCAGCTTCTCCTGGGGGGGTCACAGGCT3’
5’X-R	5’CCCAGCTTCTCCTGGGGGGCTCACAGGCT3’
5’X-ΔP4-R	5’GGGCTCACAGGCTGCACAGCGTTCCCC3’
5’G3C-R	5’CCCAGCTTCTCCTGGGGGGCTCACAGGCT3’
5’PK2mut-R	5’CCCAGCTTCTCCTGGGCCCCTCACAGGCT3’
3’WT-R	5’TTTTTTTTTTTTTTTTTTTTTTTTTTTTTTTTTTTTTTTTTTTCCCAGCTTCTC3’
3’X-R	5’TTTTTTTTTTTTTTTTTTTTTTTTTTTTTTTTTTTTTTTTTTTCCCAGCTTCTC3’
3’X-ΔP4-R	5’TTTTTTTTTTTTTTTTTTTTTTTTTTTTTTTTTTTTTTTTTTTGGGCTCACAGGC3’
3’-G3C-R	5’TTTTTTTTTTTTTTTTTTTTTTTTTTTTTTTTTTTTTTTTTTTCCCAGCTTCTC3’
3’-PK2mut-R	5’TTTTTTTTTTTTTTTTTTTTTTTTTTTTTTTTTTTTTTTTTTTCCCAGCTTCTC3

Table S8. SAXS data collection parameters and software employed for data analysis.

Data Collection Parameters		
Facilities and parameters	Settings and values	
Beam line	12ID-B (APS, ANL)	BL19U2 (SSRF)
Wavelength (Å)	0.8857	1.033
Detector	Pilatus 1M	Pilatus 100K
q range ($Å^{-1}$)	0.005-0.89	0.009-0.415
Exposure time (s)	30-60	60
Concentration range (mg/ml)	0.75-3	0.75-3
Temperature (K)	298	298
Software Employed		
Primary Data Processing	Matlab/BioXTAS RAW/PRIMUS	
P(r) Function	GNOM	
SAXS Profile Computation	CRYSOL	
Molecular Visualization	PyMol	

REFERENCES

1. Woodson SA. Metal ions and RNA folding: a highly charged topic with a dynamic future. *Curr Opin Chem Biol* **9**, 104-109 (2005).

2. Misra VK, Draper DE. On the role of magnesium ions in RNA stability. *Biopolymers* **48**, 113-135 (1998).

3. Rambo RP, Tainer JA. Accurate assessment of mass, models and resolution by small-angle scattering. *Nature* **496**, 477-481 (2013).

4. Zhang Y*, et al.* Long non-coding subgenomic flavivirus RNAs have extended 3D structures and are flexible in solution. *EMBO Rep*, e47016 (2019).

5. Bozza M, Sheardy RD, Dilone E, Scypinski S, Galazka M. Characterization of the secondary structure and stability of an RNA aptamer that binds vascular endothelial growth factor. *Biochemistry* **45**, 7639-7643 (2006).

6. Lu M, Draper DE. Bases defining an ammonium and magnesium ion-dependent tertiary structure within the large subunit ribosomal RNA. *J Mol Biol* **244**, 572-585 (1994).

7. Bottaro S, Di Palma F, Bussi G. The role of nucleobase interactions in RNA structure and dynamics. *Nucleic Acids Res* **42**, 13306-13314 (2014).

8. Whitford PC, Noel JK, Gosavi S, Schug A, Sanbonmatsu KY, Onuchic JN. An all-atom structure-based potential for proteins: bridging minimal models with all-atom empirical forcefields. *Proteins* **75**, 430-441 (2009).

9. Noel JK, Whitford PC, Onuchic JN. The shadow map: a general contact definition for capturing the dynamics of biomolecular folding and function. *J Phys Chem B* **116**, 8692-8702 (2012).

10. Noel JK*, et al.* SMOG 2: A Versatile Software Package for Generating Structure-Based Models. *PLoS Comput Biol* **12**, e1004794 (2016).

11. Suma A, Coronel L, Bussi G, Micheletti C. Directional translocation resistance of Zika xrRNA. *Nat Commun* **11**, 3749 (2020).

12. Dudko OK, Hummer G, Szabo A. Theory, analysis, and interpretation of single-molecule force spectroscopy experiments. *Proc Natl Acad Sci U S A* **105**, 15755-15760 (2008).

13. Chen AA, Garcia AE. Mechanism of enhanced mechanical stability of a minimal RNA kissing complex elucidated by nonequilibrium molecular dynamics simulations. *Proc Natl Acad Sci U S A* **109**, E1530-1539 (2012).

14. Dudko OK. Decoding the mechanical fingerprints of biomolecules. *Q Rev Biophys* **49**, e3 (2016).

15. Zhang Y, Dudko OK. A transformation for the mechanical fingerprints of complex biomolecular interactions. *Proc Natl Acad Sci U S A* **110**, 16432-16437 (2013).

16. Shim JW, Gu LQ. Encapsulating a single G-quadruplex aptamer in a protein nanocavity. *J Phys Chem B* **112**, 8354-8360 (2008).

17. Chapman EG*, et al.* The structural basis of pathogenic subgenomic flavivirus RNA (sfRNA) production. *Science* **344**, 307-310 (2014).
