# Supplementary material for: Molecular mechanisms underlying the extreme mechanical anisotropy of the flaviviral exoribonuclease-resistant RNAs (xrRNAs)
Source: Nat Commun. 2020 Oct 30;11:5496. doi: 10.1038/s41467-020-19260-4 (PMC7603331; doi:10.1038/s41467-020-19260-4)
Supplement: Supplementary file 3 — Reporting Summary [file 41467_2020_19260_MOESM3_ESM.pdf]

nature research

Corresponding author(s): Jinghong Li, Xianyang Fang

Last updated by author(s): Oct 1, 2020

Reporting Summary

Nature Research wishes to improve the reproducibility of the work that we publish. This form provides structure for consistency and transparency in reporting. For further information on Nature Research policies, see our Editorial Policies and the Editorial Policy Checklist.

Statistics

For all statistical analyses, confirm that the following items are present in the figure legend, table legend, main text, or Methods section.

n/a	Confirmed	
☐	☒	The exact sample size (*n*) for each experimental group/condition, given as a discrete number and unit of measurement
☐	☒	A statement on whether measurements were taken from distinct samples or whether the same sample was measured repeatedly
☒	☐	The statistical test(s) used AND whether they are one- or two-sided<br>*Only common tests should be described solely by name; describe more complex techniques in the Methods section.*
☒	☐	A description of all covariates tested
☒	☐	A description of any assumptions or corrections, such as tests of normality and adjustment for multiple comparisons
☐	☒	A full description of the statistical parameters including central tendency (e.g. means) or other basic estimates (e.g. regression coefficient) AND variation (e.g. standard deviation) or associated estimates of uncertainty (e.g. confidence intervals)
☒	☐	For null hypothesis testing, the test statistic (e.g. *F*, *t*, *r*) with confidence intervals, effect sizes, degrees of freedom and *P* value noted<br>*Give P values as exact values whenever suitable.*
☒	☐	For Bayesian analysis, information on the choice of priors and Markov chain Monte Carlo settings
☒	☐	For hierarchical and complex designs, identification of the appropriate level for tests and full reporting of outcomes
☒	☐	Estimates of effect sizes (e.g. Cohen's *d*, Pearson's *r*), indicating how they were calculated

Our web collection on statistics for biologists contains articles on many of the points above.

Software and code

Policy information about availability of computer code

Data collection	GRAOMACS2016.4 and GROMACS2018.6 For MD simulations; Patch-clamp amplifier for Nanopore experiment;
Data analysis	GRAOMACS2016.4, GROMACS2018.6, VMD1.9.3 GRAOMCS2018.7-FDA2.9, PLUMED2.5.1 and OriginPro2016 for MD data; MatlabR2017a and OriginPro2016 for nanopore data analysis; OriginPro2016 for DSC data analysis; MatlabR2017a, BioXTAS RAW1.3.1, PRIMUS3.2, GNOM4.6, CRYSOL2.8.3, PyMol1.8 for SAXS data analysis.

For manuscripts utilizing custom algorithms or software that are central to the research but not yet described in published literature, software must be made available to editors and reviewers. We strongly encourage code deposition in a community repository (e.g. GitHub). See the Nature Research guidelines for submitting code & software for further information.

Data

Policy information about availability of data

All manuscripts must include a data availability statement. This statement should provide the following information, where applicable:

- Accession codes, unique identifiers, or web links for publicly available datasets
- A list of figures that have associated raw data
- A description of any restrictions on data availability

The data supporting the findings of this study are available from the corresponding authors upon reasonable request. The source data underlying Figs 5a, 5b and 6b, 6e, 6g, 6h, 7c, 7e, and Supplementary Figs S2d, S2e, S6c, S10b, S11a-e and S21a-d are provided as a Source Data file.

Field-specific reporting

Please select the one below that is the best fit for your research. If you are not sure, read the appropriate sections before making your selection.

☒ Life sciences ☐ Behavioural & social sciences ☐ Ecological, evolutionary & environmental sciences

For a reference copy of the document with all sections, see nature.com/documents/nr-reporting-summary-flat.pdf

Life sciences study design

All studies must disclose on these points even when the disclosure is negative.

Sample size	Sample size for single molecule nanopore experiment is the number of translocation events as described in methods. Experiments were repeated for 3 times and N > 200 events were recorded for each experiment, which is sufficient for statistical analysis . In our computational work, we performed SMD simulations at various loading rates (i.e. ranging from 4.784 to 1661 pN/ns for 3'-end) and repeated at least 8 times for each loading rate (the slowest loading rate). The unfolding behaviors are well retained among various loading rate, so our sample size is sufficient for establishing the relationship between loading direction and mechanical stability.
Data exclusions	No data were excluded from the analysis.
Replication	The results were successfully reproduced in at least 3 independent experiments.
Randomization	Samples were not allocated into experimental groups, thus randomization is not relevant for our study
Blinding	There was no group allocation during data collection and analysis. so blinding is not necessary.

Reporting for specific materials, systems and methods

We require information from authors about some types of materials, experimental systems and methods used in many studies. Here, indicate whether each material, system or method listed is relevant to your study. If you are not sure if a list item applies to your research, read the appropriate section before selecting a response.

Materials & experimental systems

n/a	Involved in the study	
☒	☐	Antibodies
☒	☐	Eukaryotic cell lines
☒	☐	Palaeontology and archaeology
☒	☐	Animals and other organisms
☒	☐	Human research participants
☒	☐	Clinical data
☒	☐	Dual use research of concern

Methods

n/a	Involved in the study	
☒	☐	ChIP-seq
☒	☐	Flow cytometry
☒	☐	MRI-based neuroimaging
