# Supplementary material for: Molecular mechanisms underlying the extreme mechanical anisotropy of the flaviviral exoribonuclease-resistant RNAs (xrRNAs)
Source: Nat Commun. 2020 Oct 30;11:5496. doi: 10.1038/s41467-020-19260-4 (PMC7603331; doi:10.1038/s41467-020-19260-4)
Supplement: Supplementary file 2 — Peer Review File [file 41467_2020_19260_MOESM2_ESM.pdf]

REVIEWER COMMENTS

Reviewer #1 (Remarks to the Author):

A growing list of viral RNAs have been shown to harbor exonuclease-resistant RNA structures (xrRNAs) that block the progression of cellular 5′ to 3′ exonucleases. xrRNAs for which structures have been solved have a "ring-like" structure in which the 5′ end of the RNA is encircled protectively by a continuous stretch of RNA. Within the ring, the base-pairs are "tucked away" behind backbone, forming a brace-like element that is unique to these structures. This ring-like topology has been proposed to be the key to their ability to block exonucleases degrading form the 5′ side in a fairly nonspecific way, but not block molecular machines that approach from the 3′ end. This unidirectional resistance model suggests an extreme molecular anisotropy exists in these structures. Furthermore, it has been proposed, based on biochemical and structural studies, that the unique ring-like structure, produced by a set of complete tertiary interaction in the RNA 3D fold, is the source of this likely anisotropy.

Despite the evidence in support of this mechanistic model, it has never been directly tested using biophysical methods to detect the needed mechanical anisotropy. The reason, in part, is the challenge of modeling the pulling of the RNA from one end as the structure comes into contact with a "pore" that would mimic the contact with the surface of an exonuclease and pulling into its active site. Here, the authors employ single-molecule nanopore technology to detect and measure exactly these features, using xrRNAs from the flaviviruses as their subjects.

Overall, this is really beautiful work. The experiments are appropriate, well-conceived, and well executed. The data and analysis are rigorous and overall the conclusions are well supported. This work pretty definitely "nails down" the mechanism of exonuclease resistance by these xrRNAs.

My only major area of concern is the work on the HCV IRES and riboswitch elements, which I think detracts from the very clear and clean work of the rest of the paper. Specifically:

1. The authors point out the differences structural of the HCV IRES and riboswitch domains, but still call them "ring-like." I disagree. In these structures, the 5′ end is in a helical element, and the proximity to other helical elements and a pseudoknot, combined with the normal turn of the helices, places the 5′ end in between other elements. But this is very different from a true ring, in which a single continuous backbone strand almost encircles the 5′ end, and the base-pairs are inaccessible. Both HCV and riboswitch element, deviate from this, as there are large gaps in the ring, it is not a continuous strand, etc. It is a subtle, but I think an important, distinction in the topologies and features of the structure that precludes calling them a ring-like brace.

2. The authors show exonuclease resistance by the riboswitch and IRES element, but the enzyme stop sites are not mapped. Therefore, the relationship to the enzyme halt site to parts of the structure cannot be assessed and conclusions about the relationship of specific structural features to enzyme halting cannot be made. Also, I do not believe there is any evidence that these elements operate as xrRNAs in biology, so the question of the relevance must be asked.

3. The MD simulations are interesting and make some new hypothesis, but I do not find them compelling without actual force measurements on the HCV and riboswitch RNAs to verify them.

Minor concerns:
1. Page 6, last sentence of paragraph 2. "Obviously, similar as xrRNA1, xrRNA1-X exhibits significant mechanical anisotropy in 5 mM Mg^{2+}, thus, xrRNA1-X could be used to represent xrRNA1 in the subsequent quantitative analysis."

I agree that xrRNA1-X's anisotropy makes it useful in this way, but nonetheless there may be

undetected differences compared to the WT. The authors may want to state at this point a few of the limitations of used xrRNA1-X as a surrogate.

Reviewer #2 (Remarks to the Author):

In this manuscript, the authors employ a combination of nanopore sensing techniques and simulations, in order to study the differential dynamics of 3'-initiated and 5'-initiated translocation of xrRNA1. The authors clearly articulate the biological relevance of measurements made in the context of this non-biological process. The experimental measurements provide evidence that the 5'-initiated translocation is several orders of magnitude slower than 3'-initiated translocation. While I am familiar with, but not an expert on, the experimental technique being employed, the measurements seem appropriate and they would appear to support the claim that processing xrRNA via the 5' end should be disfavored in the cell. However, the simulations appear preliminary and anecdotal. Below are some significant points that should be addressed with the manuscript, as well as specific concerns about the significance of the simulations.

Major points:

1) The authors should provide a concrete and clear description of what is meant by "ring-like structure". The term is applied many times throughout the manuscript, but the definition is very vague. Based on the text, it appears that any RNA that has a pseudoknot would be "ring-like". The reason this is a major concern is that the manuscript centers around discussion of this motif and its potentially-broad relevance to biological dynamics. For example, the title of Fig 5 is "Mechanical anisotropy of xrRNA1 is mainly dictated by its ring-like structure." Without a clear definition of a ring-like motif, it is very difficult to determine whether it can be used to understand anything about the biophysical properties of RNA processing. A schematic that details the features that constitute a ring could be helpful.

2) The majority of the manuscript (all of the experiments in Figs 1-5) is focused on a single RNA, and then the simulations attempt to provide broad claims about classes of RNA. While I can appreciate the desire to attempt to generalize through the use of simulations, the discussion of other RNAs distracts from the main focus. It would be advisable that the simulations focus on the system of experimental focus. By simulating several RNAs, it appears the efforts may have been spread too thin. For example, Fig 5 shows average contact maps calculated from 100ns simulations. These are then compared to the contacts formed in the crystal structures. There are two issues with these figures. First, it appears that the simulations are simply confirming that the structure is maintained, rather than providing some form of complementary insight. The second issue is that 100ns is too short to make any meaningful statements about energetics/stability. It is not uncommon for AMBER forcefields to require microseconds to equilibrate, even for small RNAs. Upon full equilibration, it is very common that the crystal structure is not the dominant conformation. For example, see Chen and Garcia, PNAS, 2013, p16820-16825.

3) Fig 6 describes pulling simulations of three different RNAs, each pulled through a nanopore via the 3' or 5' end, or by pulling on both ends separate from a nanopore. While the SI claims shows that 16 replicas were simulated, the main text focuses on a single event. Describing a single event is not sufficient to make any meaningful claims about the energetics of a biomolecule. It would be more appropriate to report statistical aspects of the dynamics, as was done for the experimental components of the manuscript. In addition, the pulling timescale of the simulations is roughly 6 orders

of magnitude faster than the experimental time (~100ns vs seconds), which makes it unclear if the dynamics/energetics in experiments and simulations should even be compared. At such high pulling speeds, it isn't clear whether the modeled energetics are relevant, in comparison to the energetics associated with the applied steering forces. If one would like to compare these disparate timescales, there should be a comprehensive and rigorous argument presented that clearly delineates what can (and can not) be gleaned from the comparison. Without such a discussion, there is no reason to interpret the simulated dynamics.

Reviewer #3 (Remarks to the Author):

The authors of this report utilized the nanopore single molecule detection technique, mutagenesis and molecular dynamic simulation to investigate the mechanism of molecular mechanical anisotropy for a group of exoribonuclease-resistant RNA (xrRNA) in RNA viruses, including ZIKV (nanopore plus simulation), HCV IRES (simulation only) and the aptamer domain of the THF riboswitch (simulation only).

The overall work is very interesting and significant. Mechanical anisotropy is an important property of various functional biomolecules, from proteins to nucleic acids. In general, it is a property of biomolecules that shows different mechanical stability when getting mechanical stimulation in different directions. For nucleic acids, one of mechanical anisotropy properties is vectorial unfolding, i.e. unfolding from one end to the other end of the polymer. Vectorial unfolding has biological functions. Many functions such as ribosomal protein synthesis need to move along the RNA chain and unfold local structures so as to continue the processing. Exoribonuclease-resistant RNA (xrRNA) in this report is another example. Viruses may use this structure to stop degradation by exoribonuclease from the 5′ end, thus can form various functional sub-genomic RNAs. Meanwhile, RNA-dependent RNA polymerase can easily synthesize this RNA from the 3′-5′ direction without overcome a high energy barrier. Therefore, it is reasonable that xrRNA can demonstrate high stability from 5′ end while being less stable for disruption from the 3′ end. This kind of stability asymmetry is a kind of mechanical anisotropy, which has a strong basis on RNA tertiary structure. In addition, this reviewer agrees that the envisioned RNA-based mechanical anisotropy biomaterials would be a significant field of translational research in the future.

Nanopore is a tool very suitable to this study, because nanopore can induce RNA vectorial unfolding from one direction, and thus may provide rich information about mechanical anisotropy. The overall finding is that the unfolding from the 5′ end needs higher force and cost higher energy than from 3′ end of xrRNAs. The authors used a group of mutants that knock out key motifs in the tertiary structure to study their influence on the stability from each end. In this property, Mg^{2+} plays a very important role in determining the stabilization of local ring-like architecture, which is supposed to be the core of the structure in generating mechanical anisotropy.

It is innovative of using nanopore to elucidate mechanical anisotropy of exoribonuclease-resistant RNAs (xrRNAs). Just mention that the mechanical anisotropy property (or asymmetric unfolding between 3′ and 5′ directions as studied in this report) has been discovered previously by others. Please see the last question below.

Main questions

In this report, the authors measured the duration of the blocking events to characterize the RNA stability under the nanopore pulling in one direction, either from 5′ or 3′. This is correct. But the blocking duration can hardly reveal the unfolding mechanism unless the blocking current pattern is analyzed. For example, in either unfolding direction, it remains unclear which is the initial unfolding

intermediate, which is the unfolding middle state that causes permanent nanopore clogging (e.g. in Figure 2c), how these states are different between the two unfolding directions. It is understandable that this current analysis is complicated. This is because the nanopore has a cis vestibule, small motifs in RNA can be trapped inside, whereas larger motifs cannot, meaning that different motifs can unfolding at different nanopore regions. Motifs trapped inside the vestibule has a different unfolding kinetics due to the confinement in a nanometer size space. This can be partially solved by doing the following experiment: in addition to the poly A lead, which is used to lead the RNA into the pore, the authors should also measure RNA with other lead sequences. Different lead sequences produce different initial currents in the blockade, such that you can determine (1) whether the block/clogging is generated by the lead heading into the pore or the other part trapped in the pore, (2) the initial unfolding state, thus being closer to the answer of the question which motif is the key to the high stability.

Were all nanopore experiments conducted in 1 M KCl both in cis and trans solutions (in main text), or 1 M KCl in cis versus 3M KCl trans asymmetric solutions (in Method)? Please confirm. The conditions are not consistent each other in the paper. This reviewer assumes they were all under 1 M/3 M KCl condition as all observed nanopore conductance throughout the report were 2-3 folds larger than the known conventional conductance for hemolysin. The authors should provide rationales why detecting in asymmetrical KCl solutions and why using high KCl concentration as high as 3 M.

Continuing with the above question, the authors should test and compare the result in low ionic strength, for example 150-200 mM KCl. The low salt concentration may not be necessary for any nanopore work, but it is important to this work. This condition is close to the physiological environment. This test will clarify if mechanical anisotropy is enhanced or weakened under the (near) physiological condition, compared with high KCl solution. This is because exoribonuclease and many other enzymes do not function properly in high salt concentration; and at low salt concentration, RNA structures likely become less stable, thus it is expected that the nanopore might be able to unfold some RNA structures (e.g. the 5′ end unfolding) that cannot be unfolded at high ionic strength.

Among references cited, Zhang X. et al JACS 2015 and Zhang X. et al Nat Comm 2017 should be cited at several places in the study of mechanical anisotropy (or asymmetric unfolding). Both were among the first to establish RNA vectorial unfolding hypothesis (unfolding from 5′ and 3′ end has revealed different stability and kinetics), tracking RNA folding pathway, Mg2+ effect on the folding, and as in the above question, determined the salt concentration effect on the RNA stability.

Response to reviewer 1

Reviewer #1 (Remarks to the Author):

A growing list of viral RNAs have been shown to harbor exonuclease-resistant RNA structures (xrRNAs) that block the progression of cellular 5' to 3' exonucleases. xrRNAs for which structures have been solved have a "ring-like" structure in which the 5' end of the RNA is encircled protectively by a continuous stretch of RNA. Within the ring, the base-pairs are "tucked away" behind backbone, forming a brace-like element that is unique to these structures. This ring-like topology has been proposed to be the key to their ability to block exonucleases degrading form the 5' side in a fairly nonspecific way, but not block molecular machines that approach from the 3' end. This unidirectional resistance model suggests an extreme molecular anisotropy exists in these structures. Furthermore, it has been proposed, based on biochemical and structural studies, that the unique ring-like structure, produced by a set of complete tertiary interaction in the RNA 3D fold, is the source of this likely anisotropy.

Despite the evidence in support of this mechanistic model, it has never been directly tested using biophysical methods to detect the needed mechanical anisotropy. The reason, in part, is the challenge of modeling the pulling of the RNA from one end as the structure comes into contact with a "pore" that would mimic the contact with the surface of an exonuclease and pulling into its active site. Here, the authors employ single-molecule nanopore technology to detect and measure exactly these features, using xrRNAs from the flaviviruses as their subjects.

Overall, this is really beautiful work. The experiments are appropriate, well-conceived, and well executed. The data and analysis are rigorous and overall the conclusions are well supported. This work pretty definitely "nails down" the mechanism of exonuclease resistance by these xrRNAs.

Response #1: We appreciate the positive comments from Referee 1 with regard to the scientific significance about the work.

My only major area of concern is the work on the HCV IRES and riboswitch elements, which I think detracts from the very clear and clean work of the rest of the paper. Specifically:

1. The authors point out the differences structural of the HCV IRES and riboswitch domains, but still call them "ring-like." I disagree. In these structures, the 5' end is in a helical element, and the proximity to other helical elements and a pseudoknot, combined with the normal turn of the helices, places the 5' end in between other elements. But this is very different from a true ring, in which a single continuous backbone strand almost encircles the 5' end, and the base-pairs are inaccessible. Both HCV and riboswitch element, deviate from this, as there are large gaps in the ring, it is not a continuous strand, etc. It is a subtle, but I think an important, distinction in the topologies and features of the structure that precludes calling them a ring-like brace.

Response #2: We agree with reviewer 1's comments and it's inappropriate to call the HCV IRES and THF riboswitch elements as ring-like structures because of the topological differences. Enlightened by the comments from both reviewer 1 and reviewer 2 (**see below**), we decide to stay focused on ZIKV xrRNA1 work and remove the work on the HCV IRES and riboswitch elements in the revised manuscript.

2. The authors show exonuclease resistance by the riboswitch and IRES element, but the enzyme stop sites are not mapped. Therefore, the relationship to the enzyme halt site to parts of the structure cannot be assessed and conclusions about the relationship of specific structural features to enzyme halting cannot be made. Also, I do not believe there is any evidence that these elements operate as xrRNAs in biology, so the question of the relevance must be asked.

3. The MD simulations are interesting and make some new hypothesis, but I do not find them compelling without actual force measurements on the HCV and riboswitch RNAs to verify them.

Response #3: Reviewer 1's comments prompt us to think thoroughly about the relevance of the IRES and riboswitch elements. A previous report (*XRN1 Stalling in the 5' UTR of Hepatitis C Virus and Bovine Viral Diarrhea Virus Is Associated with Dysregulated Host mRNA Stability*. ***PLoS pathogen*** **2015, 11: e1004708**) has identified the possible Xrn1 halting sites in the HCV-IRES structure (**Fig. 1a-b**), we verified the halting site on the construct used in our study by *in vitro* assay (**Fig. 1c**). We also carried out experiments to identify the Xrn1 halting sites of THF riboswitch which is mapped to be close to the 5' end duplex (around G1, G2) (**Fig. 1d-f**). Although our *in vitro* assays confirm Xrn1 stalling by both HCV IRES and THF riboswitch elements (**Fig. 1g**), we can't provide any clues about the biological relevance at this moment which future work is needed. **To keep the whole work clean and clear, we thus remove the work on HCV IRES and THF riboswitch elements from the manuscript and focus on the xrRNA1 work.**

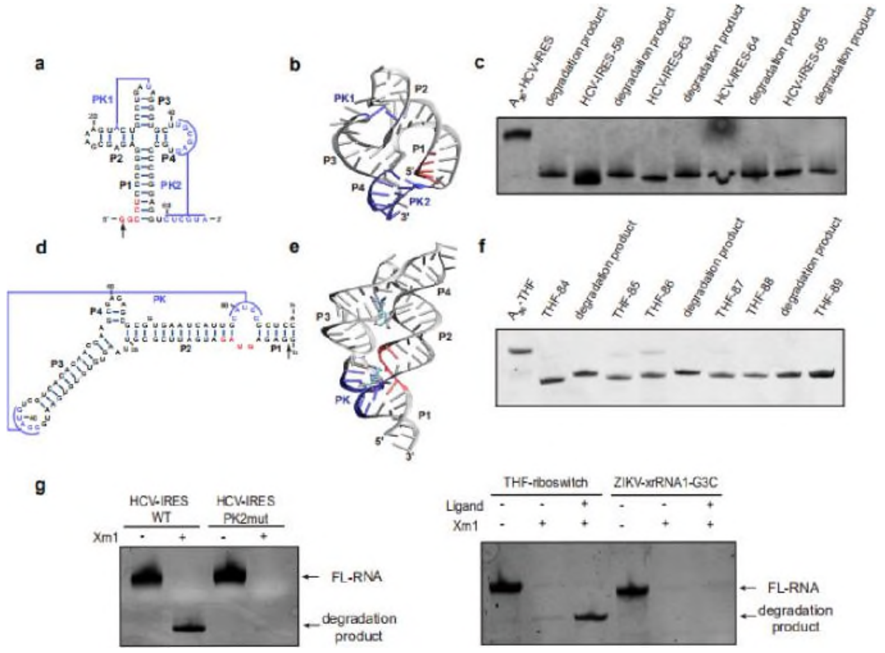

Fig. 1. Xrn1 resistance activity of the HCV-IRES and THF riboswitch RNA elements. (**a-c**) Secondary structure (**a**) and tertiary structure (**b**) of the HCV-IRES element. The Xrn1 halting site on HCV-IRES is mapped by comparing the shift rates of the degradation product of A_{36}-HCV-IRES with HCV-IRES transcripts of different lengths (59, 63, 64, 65 nucleotides) using 15% TBE-Urea denaturing PAGE (**c**) and indicated with an arrow in (**a**). The halting site is consistent with previous report (***PLoS pathogen* 2015, 11: e1004708**). (**d-f**) Secondary structure (**d**) and tertiary structure (**e**) of the THF riboswitch element. The Xrn1 halting site on THF riboswitch is mapped by comparing the shift rates of the degradation product of A_{36}-THF riboswitch with THF riboswitch transcripts of different lengths (84, 85, 86, 87, 88, 89 nucleotides) using 15% TBE-Urea denaturing PAGE (**f**) and indicated with an arrow in (**d**). (**g**) Both the HCV-IRES and THF riboswitch element in the presence of ligand show prominent Xrn1 resistance activity.

Minor concerns:

1. Page 6, last sentence of paragraph 2. "Obviously, similar as xrRNA1, xrRNA1-X exhibits significant mechanical anisotropy in 5 mM Mg^{2+}, thus, xrRNA1-X could be used to represent xrRNA1 in the subsequent quantitative analysis."

I agree that xrRNA1-X's anisotropy makes it useful in this way, but nonetheless there may be undetected differences compared to the WT. The authors may want to state at this point a few of the limitations of used xrRNA1-X as a surrogate.

Response #4: We agree the quadruple mutations in xrRNA1-X may cause undetected differences compared to the WT, such as the folding kinetics, although the stability and overall structures are similar. We add one sentence to state this limitations in the revised text as "It should be noted that the disruption of some tertiary interactions by the quadruple mutations in xrRNA1-X may cause some undetected differences in folding kinetics or intermediates as compared with xrRNA1, although their stability and overall structure are very similar."

Response to reviewer 2

Reviewer #2 (Remarks to the Author):

In this manuscript, the authors employ a combination of nanopore sensing techniques and simulations, in order to study the differential dynamics of 3'-initiated and 5'-initiated translocation of xrRNA1. The authors clearly articulate the biological relevance of measurements made in the context of this non-biological process. The experimental measurements provide evidence that the 5'-initiated translocation is several orders of magnitude slower than 3'-initiated translocation. While I am familiar with, but not an expert on, the experimental technique being employed, the measurements seem appropriate and they would appear to support the claim that processing xrRNA via the 5' end should be disfavored in the cell. However, the simulations appear preliminary and anecdotal. Below are some significant points that should be addressed with the manuscript, as well as specific concerns about the significance of the simulations.

Response #5: We appreciate reviewer 2's positive comments on our experimental work on xrRNA1 and have addressed the concerns on our simulation work point-by-point as below.

Major points:

1) The authors should provide a concrete and clear description of what is meant by "ring-like structure". The term is applied many times throughout the manuscript, but the definition is very vague. Based on the text, it appears that any RNA that has a pseudoknot would be "ring-like". The reason this is a major concern is that the manuscript centers around discussion of this motif and its potentially-broad relevance to biological dynamics. For example, the title of Fig 5 is "Mechanical anisotropy of xrRNA1 is mainly dictated by its ring-like structure." Without a clear definition of a ring-like motif, it is very difficult to determine whether it can be used to understand anything about the biophysical properties of RNA processing. A schematic that details the features that constitute a ring could be helpful.

Response #6: Not any RNA that has a pseudoknot could be called as "ring-like". Actually, the 'ring-like structure' is firstly defined by Jeffrey Kieft group by studying the topology of the crystal structures of ZIKV xrRNA1 and MVEV xrRNA2 (see references 13 and 14), which the 5' end of the RNA is encircled protectively by a continuous stretch of RNA and the base-pairs are "tucked away" behind backbone within the ring, forming a brace-like element that is unique to these structures (also see reviewer 1's comments). **In light of reviewer 1's comment, we decide not to call the HCV IRES and the THF riboswitch elements as ring-like structures because of their large topology differences and remove the work in revised manuscript**, now the definition of ring-like structure become concrete and clear and Figure 1c is suitable to show the feature of the ring structure. We also modify the definition of the ring-like structure in the revised text (see line 2, paragraph 2 and page 2).

2a) The majority of the manuscript (all of the experiments in Figs 1-5) is focused on a single RNA, and then the simulations attempt to provide broad claims about classes of RNA. While I can appreciate the desire to attempt to generalize through the use of simulations, the discussion of other RNAs distracts from the main focus. ***It would be advisable that the simulations focus on the system of experimental focus.*** *By simulating several RNAs, it appears the efforts may have been spread too thin.*

Response #7: We appreciate and take the advices from Reviewer 2. In the revised manuscript, we remove the work on HCV-IRES and THF riboswitch elements and focus on MD simulations of ZIKV xrRNA1.

2b) For example, Fig 5 shows average contact maps calculated from 100ns simulations. These are then compared to the contacts formed in the crystal structures. There are two issues with these figures. First, it appears that the simulations are simply confirming that the structure is maintained, rather than providing some form of complementary insight. The second issue is that 100ns is too short to make any meaningful statements about energetics/stability. It is not uncommon for AMBER forcefields to require microseconds to equilibrate, even for small RNAs. Upon full equilibration, it is very common that the crystal structure is not the dominant conformation. For example, see Chen and Garcia, PNAS, 2013, p16820-16825.

Response #8: We agree with Reviewer 2's constructive criticisms that our MD simulations should provide complementary insight rather than simply confirm experimental findings. We performed additional simulations, did new analysis and discussions, and revised the text and Supporting Information accordingly.

To explore ZIKV xrRNA1's conformational space, we extended equilibrium MD simulations up to 1450 ns (**Figure S19**). Principal Component Analysis (PCA) revealed that the dynamic motions of ZIKV xrRNA1 are predominated by twist motions along long axis and bending motions between pseudoknot 2 (PK2) and P4, resulting in local structural differences between crystal structure and MD ensembles. For example, the GAAA tetraloop capped on P2 (tetraloop 2) experiences fast conformational switching between non-native (crystal structure) and native state (refolding). Nonetheless, the average structure derived from PCA analysis is very similar to the crystal structure with RMSD of ~3 Å. In line with the SAXS experiments, our MD simulations confirmed that the essential structural features (i.e. the ring-like architecture) observed in ZIKV xrRNA1 crystal structure are

predominated in solution. The relevant discussions are added in Page 4, SI.

Besides the original SMD simulations, we performed new simulations and added new analysis (such as **force distribution analysis**) and discussion to illustrate the physical origin of mechanical anisotropy in main text (**Figure 7**) and SI (**Figure S15-18**). We found that the tertiary interaction network in the 5'-end is highly coupled upon forces loaded from 5'-end, which will redistribute the forces into broader region, rendering its high resistance to the loading force; by contrast, when forces is exerted on the 3'-end which complex tertiary interaction network is absent, the force is confined on break point or local segments, lowering the mechanical resistance through an unzipping fashion (**Figure 7**).

3) Fig 6 describes pulling simulations of three different RNAs, each pulled through a nanopore via the 3' or 5' end, or by pulling on both ends separate from a nanopore. While the SI claims shows that 16 replicas were simulated, the main text focuses on a single event. Describing a single event is not sufficient to make any meaningful claims about the energetics of a biomolecule. It would be more appropriate to report statistical aspects of the dynamics, as was done for the experimental components of the manuscript. In addition, the pulling timescale of the simulations is roughly 6 orders of magnitude faster than the experimental time (~100ns vs seconds), which makes it unclear if the dynamics/energetics in experiments and simulations should even be compared. At such high pulling speeds, it isn't clear whether the modeled energetics are relevant, in comparison to the energetics associated with the applied steering forces. If one would like to compare these disparate timescales, ere should be a comprehensive and rigorous argument presented that clearly delineates what can (and can not) be gleaned from the comparison. Without such a discussion, there is no reason to interpret the simulated dynamics.

Response #9: We appreciate Reviewer 2's constructive comments, which have two major concerns.

The first relates to the statistical aspects of the dynamics. In our original submission, we performed 16 independent SMD simulations on xrRNA1 unfolding at various loading rates from 5'-end pulling (0.01 nm/ns, 0.1 nm/ns, 1 nm/ns), 3'-end pulling (0.1 nm/ns) and two-ends pullinig (0.1 nm/ns). To have a better statistics on the rupture forces by 5'-end or 3'-end pulling, we further performed 16 independent SMD simulations on xrRNA1 from 5'-end pulling (0.005 nm/ns) and on xrRNA1-ΔP4 from 3'-end pulling (0.008 nm/ns, 0.01 nm/ns, 0.1 nm/ns, 1 nm/ns).

We observed two major unfolding events in the initial translocation regime (i.e translocation displacement is 0 ~ 60 Å) of xrRNA1 by 5'-end pulling, which correspond to two distinctive peaks in the force profile. The unfolding pattern and correlation between unfolding events and rupture forces are similar among the SMD simulations for xrRNA1 under 5'-end pulling at different pulling rates. The rupture forces for major unfolding events, as well as the mean force and its variance at each pulling rates, are summarized in **Figure 6h**. These averaged rupture forces follow logarithmic scaling with loading rate (described by $r=\kappa v$, κ and v are the spring constant and pulling velocity, respectively). To better illustrate this correlation, we plotted exemplary force profile and rupture event for xrRNA1 at each pulling rates (**Figure S15**).

To further assess the mechanical stability of xrRNA1 along the 3'→5' direction, we performed a series of new SMD simulations to obtain statistics on the rupture force of PK2 of xrRNA1-ΔP4 by 3'-end pulling at different loading rates (range from 0.008 nm/ns to 1 nm/ns) (**Figure 6h** and **S17**). The rupture force of PK2 in xrRNA1-ΔP4 along 3'→5' direction was used to reduce computational cost. Such a simplification was based on two observations: 1) nanopore experiments suggested that removal of P4 has only minimal impact on the mechanostability of xrRNA1; 2) due to the nature of sequential unzipping manner during xrRNA1-ΔP4 translocation along 3'→5' direction, the rupture forces for PK2 is comparable to other major peaks in the force profile. These rupture forces under different loading rates is also included in **figure 6h,** and their mean forces roughly follow logarithmic scaling with the loading rate.

From the SMD analysis, it's apparent the resistance force by xrRNA1 to 3'-end pulling is much smaller than that to 5'-end pulling. The mechanical anisotropy of ZIKV xrRNA1 revealed by SMD simulations, to a certain degree, is consistent with the observations from nanopore experiments.

The second relates to the differences in the timescale between the simulation and experimental results. To characterize the unfolding events of xrRNA1 in tractable simulation time, we employed a loading rate with several order of magnitude higher than that in nanopore experiments, which will cause unfolding rate in the simulations much faster than that in experiments. In principle, we can extrapolate unfolding rate to a lower loading regime using an appropriate theoretical model, such as Bell-Evan, thus the computational and experimental results in timescale become comparable. However, the kinetic rates k_0 at zero force for barrier 1 and 2 (corresponding to peak 1 and 2 in **Figure 6a**) derived from our high loading rate using Bell-Evan formula are $2.9\times10_4$ s^{-1} and 6.24×10^2 s^{-1}, respectively, which are at least five orders of magnitude faster than that from experimental ones (smaller than 3.0×10^{-3} s^{-1}). We added relevant discussions on the reasons and the limitations of extrapolation in main text and SI.

<u>Response to reviewer 3</u>

Reviewer #3 (Remarks to the Author):

The authors of this report utilized the nanopore single molecule detection technique, mutagenesis and molecular dynamic simulation to investigate the mechanism of molecular mechanical anisotropy for a group of exoribonuclease-resistant RNA (xrRNA) in RNA viruses, including ZIKV (nanopore plus simulation), HCV IRES (simulation only) and the aptamer domain of the THF riboswitch (simulation only). The overall work is very interesting and significant. Mechanical anisotropy is an important property of various functional biomolecules, from proteins to nucleic acids. In general, it is a property of biomolecules that shows different mechanical stability

when getting mechanical stimulation in different directions. For nucleic acids, one of mechanical anisotropy properties is vectorial unfolding, i.e. unfolding from one end to the other end of the polymer. Vectorial unfolding has biological functions. Many functions such as ribosomal protein synthesis need to move along the RNA chain and unfold local structures so as to continue the processing. Exoribonuclease-resistant RNA (xrRNA) in this report is another example. Viruses may use this structure to stop degradation by exoribonuclease from the 5' end, thus can form various functional sub-genomic RNAs. Meanwhile, RNA-dependent RNA polymerase can easily synthesize this RNA from the 3'-5' direction without overcome a high energy barrier. Therefore, it is reasonable that xrRNA can demonstrate high stability from 5' end while being less stable for disruption from the 3' end. This kind of stability asymmetry is a kind of mechanical anisotropy, which has a strong basis on RNA tertiary structure. In addition, this reviewer agrees that the envisioned RNA-based mechanical anisotropy biomaterials would be a significant field of translational research in the future.

Nanopore is a tool very suitable to this study, because nanopore can induce RNA vectorial unfolding from one direction, and thus may provide rich information about mechanical anisotropy. The overall finding is that the unfolding from the 5' end needs higher force and cost higher energy than from 3' end of xrRNAs. The authors used a group of mutants that knock out key motifs in the tertiary structure to study their influence on the stability from each end. In this property, Mg2+ plays a very important role in determining the stabilization of local ring-like architecture, which is supposed to be the core of the structure in generating mechanical anisotropy.

It is innovative of using nanopore to elucidate mechanical anisotropy of exoribonuclease-resistant RNAs (xrRNAs). Just mention that the mechanical anisotropy property (or asymmetric unfolding between 3' and 5' directions as studied in this report) has been discovered previously by others. Please see the last question below.

Response #10: We appreciate the positive comments from Referee 3 with regard to

the scientific significance, the innovative technique, the potential translational applications about the work. We have addressed the concerns point-by-point as below.

Main questions

In this report, the authors measured the duration of the blocking events to characterize the RNA stability under the nanopore pulling in one direction, either from 5' or 3'. This is correct. But the blocking duration can hardly reveal the unfolding mechanism unless the blocking current pattern is analyzed. For example, in either unfolding direction, it remains unclear which is the initial unfolding intermediate, which is the unfolding middle state that causes permanent nanopore clogging (e.g. in Figure 2c), how these states are different between the two unfolding directions. It is understandable that this current analysis is complicated. This is because the nanopore has a cis vestibule, small motifs in RNA can be trapped inside, whereas larger motifs cannot, meaning that different motifs can unfolding at different nanopore regions. Motifs trapped inside the vestibule has a different unfolding kinetics due to the confinement in a nanometer size space. This can be partially solved by doing the following experiment: in addition to the poly A lead, which is used to lead the RNA into the pore, the authors should also measure RNA with other lead sequences. Different lead sequences produce different initial currents in the blockade, such that you can determine (1) whether the block/clogging is generated by the lead heading into the pore or the other part trapped in the pore, (2) the initial unfolding state, thus being closer to the answer of the question which motif is the key to the high stability.

Response #11: As reviewer 3 points out, detailed analysis of the current blockade signature is complicated and it's correct to measure the duration time of the current blockade event to characterize the directional mechanical stability of RNA, in our original submission, we therefore mainly analyze the directional mechanical stability of ZIKV xrRNA1 and its mutants by measuring the duration time before directional

translocation.

In response to reviewer 3's suggestions, we firstly designed C_{36} and $(CAA)_{12}$ 5' leader sequences, but the plasmid containing C_{36} is difficult to synthesize chemically because of high G+C content, and the RNA transcript with $(CAA)_{12}$ leader become aggregated evidenced with smaller elution volume compared to xrRNA1 with poly(rA_{36}) in the size exclusion chromatography profile (data not shown), thus xrRNA1 with $(CAA)_{12}$ leader has different structure and is not suitable for nanopore sensing experiment. We therefore chose to measure xrRNA1s with different 5' poly(rA) leader sequences in length (from A_{13} to A_{30}). As shown in **Figure S6**, the initial drop of ionic current decreases as the length of leader sequence increases from A_{13} to A_{20}, further increasing the length of the leader sequence cause little changes in the initial drop of ionic current. Based on these observations, we can assign that the initial drop of the current blockade of ZIKV xrRNA1 and its mutants is generated by the poly(rA_{36}) leader sequence entering into the pore. We added these data in the revised text and **Figure S6**.

Inspired by reviewer 3's comments, we carefully analyzed the current blockade traces for directional translocation of xrRNA1, xrRNA1-X and their mutants in the presence of 5 mM Mg^{2+} or EDTA guided by 5' (**Fig. 2**) or 3' (**Fig. 2**) leaders. It's interesting to find that the current blockade traces guided by 5' leader can be roughly divided into four stages (i-ii(iia/b)-iii-iv) based on the amplitude of the current drop (**Fig. 2**). By comparing the similarities and differences of the patterns among the xrRNA1 variants (**Fig. 2b-h**), hypothesis to assign the four stages can be drawn (**Fig. 2a**), for example, stage i is the initial drop of ionic current generated by the leader sequence entering the pore, stage iia corresponds to the cooperative unfolding of the key motifs including P1, P2, PK1 and Mg^{2+}-chelating sites, stage iib corresponds to the unfolding of the key motifs including P3 and PK2, stage iii corresponds to the unfolding of P4, stage iv is the conclusion of the translocation and return to the open pore. Characteristic patterns in the current blockade traces guided by 3' leader can also be observed and the key motifs responsible for the respective stages are proposed (**Fig. 3**). We are excited

about these patterns and the hypothesis, however, to explicitly assign these patterns and stages, more experimental work is needed.

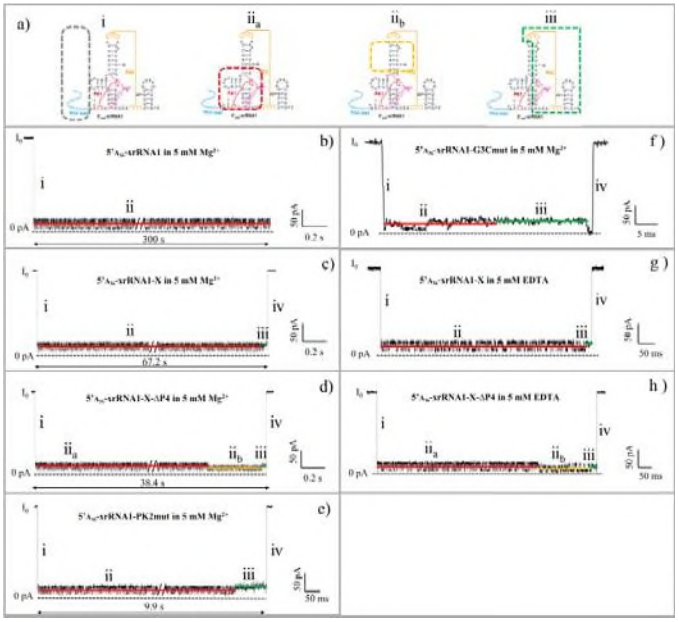

Fig. 2. The current blockade traces of xrRNA1, xrRNA1-X and their mutants in the presence of 5 mM Mg^{2+} or 5 mM EDTA guided by 5’ leader. The current block traces can be roughly divided into four stages (i, iia, red, iib, yellow, iii, green, iv **in b-h**). Hypothesis to assign the key motifs (colored accordingly in **a**) responsible for the respective phases are drawn. **It should be noted that these are only hypothesis and note validated with experiments yet.**

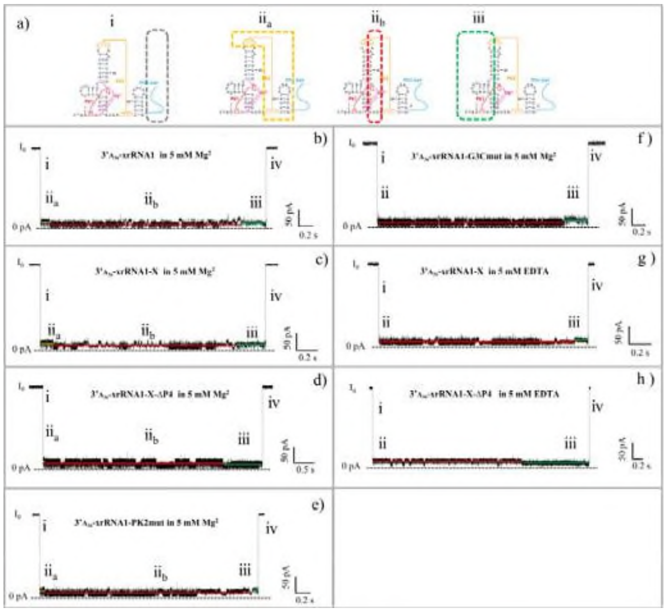

Fig. 3. The current blockade traces of xrRNA1, xrRNA1-X and their mutants in the

presence of 5 mM Mg^{2+} or 5 mM EDTA guided by 3' leader. The current block traces can be roughly divided into four phases (i, iia, yellow, iib, red, iii, green, iv **in b-h**). Hypothesis to assign the key motifs (colored accordingly in **a**) responsible for the respective phases are drawn. **It should be noted that these are only hypothesis and note validated with experiments yet.**

After studying the literature including the references listed by reviewer 3, we feel that it's very promising to assign these stages definitely by site-specific labeling of xrRNA1 with certain probes and nanopore sensing, under the premise that site specific labeling will not change the structure, stability and folding pathway. It would be great if the directional unfolding mechanism could be revealed for such a complex RNA like xrRNA1, such an example would further expand the potential of nanopore sensing in RNA structure and mechanistic studies, rather than sequencing. We believe it would be better to report the details of translocation and unfolding mechanisms with new data in future work. We focus on the directional mechanical stability and mechanical anisotropy of xrRNA1 in this work.

Were all nanopore experiments conducted in 1 M KCl both in cis and trans solutions (in main text), or 1 M KCl in cis versus 3M KCl trans asymmetric solutions (in Method)? Please confirm. The conditions are not consistent each other in the paper. This reviewer assumes they were all under 1 M/3 M KCl condition as all observed nanopore conductance throughout the report were 2-3 folds larger than the known conventional conductance for hemolysin. The authors should provide rationales why detecting in asymmetrical KCl solutions and why using high KCl concentration as high as 3 M.

Response #12: We feel sorry about the confusion in the main text and methods. We confirm that all the nanopore experiments were conducted in 1M (*cis*) / 3M (*trans*) asymmetrical KCl solutions because of enhanced frequency of the translocation events in such conditions. Such an asymmetric solution design has been applied in other studies (*Zhang X. et al JACS 2015*). We add this explanation why using such an asymmetrical KCl solution in detection (see paragraph 2, page 5) and clear up the

confusion at several places (**Paragraph 1 in page 4; Figure caption, Figure 2 in page 6; Paragraph 1 in page 6**) in the revised main text accordingly.

Continuing with the above question, the authors should test and compare the result in low ionic strength, for example 150-200 mM KCl. The low salt concentration may not be necessary for any nanopore work, but it is important to this work. This condition is close to the physiological environment. This test will clarify if mechanical anisotropy is enhanced or weakened under the (near) physiological condition, compared with high KCl solution. This is because exoribonuclease and many other enzymes do not function properly in high salt concentration; and at low salt concentration, RNA structures likely become less stable, thus it is expected that the nanopore might be able to unfold some RNA structures (e.g. the 5' end unfolding) that cannot be unfolded at high ionic strength.

Response #13: We agree with reviewer 3's suggestion and have performed nanopore sensing experiments for 5'A36-xrRNA1 and 3'A36-xrRNA1 in 200 mM KCl supplemented with 5 mM Mg^{2+} at higher voltage (+ 180 mV). The current blockade signature, the duration time for both RNA constructs were similar to that in 1 M KCl (*cis)*/3 M KCl (*trans*)with 5 mM Mg^{2+}, indicating that in the presence of 5 mM Mg^{2+}, the KCl salt concentration (0.2-3 M) has a minor effect on the mechanical anisotropy of xrRNA1. But high salt concentration will enhance the frequency of the translocation events and maintain good signal-to-noise ratio during sensing. This result is in line with the SAXS data that 5 mM Mg^{2+} is efficient to promote stable folding of xrRNA1. We add these data to **Figure 2** and a paragraph in the revised text.

*Among references cited, Zhang X. et al JACS 2015 and Zhang X. et al Nat Comm 2017 should be cited at several places **in the study of mechanical anisotropy** (or asymmetric unfolding). Both were among the first to establish RNA vectorial unfolding hypothesis (unfolding from 5' and 3' end has revealed different stability and kinetics), tracking RNA folding pathway, Mg^{2+} effect on the folding, and as in the*

above question, determined the salt concentration effect on the RNA stability.

Response #14: We have updated the citations at the several appropriate places in the revised text as Reviewer 3 suggests.

Thanks again for the comments and valuable suggestions to improve our manuscript.

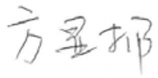

Yours sincerely,

Xianyang Fang, Ph.D.

Assistant Professor
School of Life Sciences
Tsinghua University
Beijing 100084, China
Office phone : 86-10-62771071
Cell phone: 86-18612937900
Email: fangxy@tsinghua.edu.cn

REVIEWER COMMENTS

Reviewer #1 (Remarks to the Author):

Thank you for the opportunity to re-review this exciting work, which I was enthusiastic about in my first review but had reservations about the HCV IRES and riboswitch parts of the study.

The authors have addressed all of my concerns and I fully support acceptance of this excellent contribution.

Reviewer #2 (Remarks to the Author):

Overall, the revisions have significantly improved the manuscript, where some of the earlier questions have been satisfactorily addressed. Refocusing the simulations on a single system makes the analysis more transparent and comprehensive. There are a few minor points and one significant point that should be address, as described below.

Major point:

Page 4 states that molecular machines exert a force of ~8pN. However, the simulations were performed at loading rates of 8.3 to 1661 pN/ns. While checking a range of useful, since it shows there is some form of robustness, the rates are still extremely fast. That is, even under the slowest loading rate, the applied force will be 20 times the estimated experimental force after only 20 ns. Since the reorganization time of biomolecules is typically on scales of microseconds (e.g. folding pre-factors for small proteins are ~1/microsecond, according to many studies by Eaton, Thirumalai and others). Accordingly, at these rates, the pulling force will certainly dominate all energetics in the simulations, and interpretation of the dynamics should be made with extreme caution. In particular, the paragraph beginning with the following phrase should be modified:

"It is quite surprising that one G3-C44 watson-crick pair together with one U4·A24 Hoogsteen pair could withstand considerable loading force from 5′-end (550 ~1000 pN, varying with the loading rates), comparable to the force required to unravel extreme mechanostable protein"

The reason this is a misleading claim is that, since the simulations reach a force of 1000pN after only ~120 ns (in the slowest case), lack of unfolding does not imply stability. Remaining formed on a timescale of 100ns does not mean the interaction is resistant to force, or that it is even near a free-energy minimum. While the steered MD simulations should not be used to make direct claims about stability of an individual system, the comparison of 5' vs. 3' resistance can still be meaningful. Accordingly, it would be appropriate to focus on the comparison of the two systems, rather than making specific energetic claims about a single process.

Minor points:

1) page 5 "These initial observations suggest that the kinetics of 5′A36-xrRNA1 unfolding and translocation through the α-HL nanopore is too slow to be monitored in a reasonable time window." This statement should be more precise. In particular, "reasonable" is a very vague term. In the preceding sentences, it appears the experiment is only performed for 5 minutes. It isn't obvious why 5 minutes would be unreasonable. Perhaps the authors simply mean that the timescales are not practical to study, due to a specific experimental limitation.

2) page 5 "Analysis of >200 of the events reveals four types of current blockade signatures, which are shown in Figure S8. Type I represents the most typical events and constitutes more than 85% of all

the events. Moreover, the blockade signal is different from that for xrRNA1 without a leader sequence, which generates a two-stage blockade current pattern (Figure S9). Therefore, type I signal is attributed to 3’→5’ directional translocation guided by the leader sequence and the following dwell time statistical analysis are all based on type I." Since this is central to the interpretation of all claims in this manuscript, it seems unusual that the examples of what can be seen are all relegated to the SI. It would be worthwhile to provide a brief overview of the types of events that can be detected, in the main text.

3) page 5 "To preclude the effect of high salt concentration on the directional mechanical stability of ZIKV xrRNA1..." It is not clear what effect this statement is referring to. Perhaps there is a grammatical issue.

4) page 5 "While about 20% of 5’A36-xrRNA1 translocate through the nanopore channel with duration time longer than 100 s, the remaining 80% still can’t translocate through the pore even in a 300 s time window." Since these are experiments, and not a proof, it is not appropriate to say "can't". Instead, the statement should document what is, and is not, seen. Simply replacing "can't" with "is not observed to" would be appropriate.

5) The MD section is entitled "Mechanical anisotropy of ZIKV xrRNA1 by MD simulations.". I think there is something missing (e.g. "probed by MD simulations"?)

6) In the MD section, there is the statement "To further support the hypothesis and gain insights into the mechanisms underlying ZIKV xrRNA1 mechanical anisotropy, we used steered MD...". It is not scientifically sound to claim that something is done in order to support the hypothesis. A hypothesis can be tested. However, this phrasing implies that the hypothesis was known to be true, and that simulations were simply performed in order to support it. If the simulations did not support the hypothesis, would the hypothesis or simulations be considered false? Replacing "further support the" with "further explore the" would be appropriate.

7) To finish with a comment, and not a criticism, the finding that the stress distribution in the structure-based SMOG model is centered around PK2 provides strong evidence that the architecture of the fold is responsible for the differential resistance to force, rather than energetic interactions. As a note, there are numerous typos in the methods and main text of this section, but they can be easily fixed. (e.g. "structure-base model" should be "structure-based model". "salvation" should be "solvation".) The authors should also provide a full technical description in the SI that details the simulations with SMOG models(e.g. timestep, simulation duration, temperature, etc), such that can be reproduced by others.

Additionally, Since the simulations with a SMOG model are quite similar to another recently reported set of simulations describing directional translocation ("Directional translocation resistance of Zika xrRNA" by Suma at el), there should be a brief description of the similarities and differences between the current simulations and the previously published results.

Reviewer #3 (Remarks to the Author):

After reading the revised report, this reviewer feels that the authors have conducted a series of new experiments in response to the reviewers' questions. These experimental results support the conclusion. Therefore the paper can be published as it.

Response to reviewer 2

Reviewer #2 (Remarks to the Author):

Overall, the revisions have significantly improved the manuscript, where some of the earlier questions have been satisfactorily addressed. Refocusing the simulations on a single system makes the analysis more transparent and comprehensive. There are a few minor points and one significant point that should be addressed, as described below.

Major point:

Page 4 states that molecular machines exert a force of ~8 pN. However, the simulations were performed at loading rates of 8.3 to 1661 pN/ns. While checking a range of useful, since it shows there is some form of robustness, the rates are still extremely fast. That is, even under the slowest loading rate, the applied force will be

20 times the estimated experimental force after only 20 ns. Since the reorganization time of biomolecules is typically on scales of microseconds (e.g. folding pre-factors for small proteins are ~1/microsecond, according to many studies by Eaton, Thirumalai and others). Accordingly, at these rates, the pulling force will certainly dominate all energetics in the simulations, and interpretation of the dynamics should be made with extreme caution. In particular, the paragraph beginning with the following phrase should be modified:

"It is quite surprising that one G3-C44 watson-crick pair together with one U4·A24 Hoogsteen pair could withstand considerable loading force from 5'-end (550 ~1000 pN, varying with the loading rates), comparable to the force required to unravel extreme mechanostable protein"

The reason this is a misleading claim is that, since the simulations reach a force of 1000 pN after only ~120 ns (in the slowest case), lack of unfolding does not imply stability. Remaining formed on a timescale of 100 ns does not mean the interaction is resistant to force, or that it is even near a free-energy minimum. While the steered MD simulations should not be used to make direct claims about stability of an individual system, the comparison of 5' vs. 3' resistance can still be meaningful. Accordingly, it would be appropriate to focus on the comparison of the two systems, rather than making specific energetic claims about a single process.

Response #1: We agree with the referee's comments on RNA stability in SMD and revised the paragraph accordingly: *"It is quite surprising that the rupture force for one G3-C44 pair together with one U4·A24 Hoogsteen pair from 5'-end at loading rate of 166.1 pN/ns is almost twice as the force required to unravel a full-length xrRNA1 along opposite direction (800 pN vs 400 pN, Figure 6h and 6e)."*

Minor points:

1) page 5 "These initial observations suggest that the kinetics of 5'A36-xrRNA1

unfolding and translocation through the α-HL nanopore is too slow to be monitored in a reasonable time window." This statement should be more precise. In particular, "reasonable" is a very vague term. In the preceding sentences, it appears the experiment is only performed for 5 minutes. It isn't obvious why 5 minutes would be unreasonable. Perhaps the authors simply mean that the timescales are not practical to study, due to a specific experimental limitation.

Response #2: We feel sorry about the confusion. The nanopore sensing experiment is limited by vulnerability of the α-HL nanopore which prolonged collecting time in a single experiment will not only destruct the nanopore system but cause unpractical experimental time required for collecting enough single-molecule measurements for good statistics. We have amended the main text accordingly.

2) page 5 "Analysis of >200 of the events reveals four types of current blockade signatures, which are shown in Figure S8. Type I represents the most typical events and constitutes more than 85% of all the events. Moreover, the blockade signal is different from that for xrRNA1 without a leader sequence, which generates a two-stage blockade current pattern (Figure S9). Therefore, type I signal is attributed to 3'→5' directional translocation guided by the leader sequence and the following dwell time statistical analysis are all based on type I." Since this is central to the interpretation of all claims in this manuscript, it seems unusual that the examples of what can be seen are all relegated to the SI. It would be worthwhile to provide a brief overview of the types of events that can be detected, in the main text.

Response #3: We appreciate the referee's suggestions and revised the main text accordingly, *"Analysis of >200 of the events reveals four types of current blockade signatures, which are shown in **Figure S8**. Type I represents the most typical events and constitutes more than 85% of all the events. Type II is characterized with mean duration time less than 1 ms, which could be the translocation signals of single-stranded RNAs. Type III typically show simple deep blockade current level ($\%I/I_0$ = 10%) and short duration time (~0.16s), which should be the signals for*

*partially folded xrRNA1. The remaining 6% forms Type IV, which generates a two-stage blockade current pattern, similar to that produced by xrRNA1 without a leader sequence (**Figure S9**). Types II-IV do not feature the directional unfolding and translocation guided by the leader sequence. Type I signal is attributed to 3'→5' directional translocation guided by the leader sequence and the following dwell time statistical analysis are all based on type I.*"

3) page 5 "To preclude the effect of high salt concentration on the directional mechanical stability of ZIKV xrRNA1..." It is not clear what effect this statement is referring to. Perhaps there is a grammatical issue.

Response #4: To make the statement clear, we revised the main text as "*To investigate whether ZIKV xrRNA1 exhibits similar mechanical anisotropy at low salt concentrations, we also performed nanopore sensing experiments for 5'$_{A36}$-xrRNA1 and 3'$_{A36}$-xrRNA1 in near physiological conditions (0.2 M KCl, 5 mM Mg^{2+})[19].*"

4) page 5 "While about 20% of 5'A36-xrRNA1 translocate through the nanopore channel with duration time longer than 100 s, the remaining 80% still can't translocate through the pore even in a 300 s time window." Since these are experiments, and not a proof, it is not appropriate to say "can't". Instead, the statement should document what is, and is not, seen. Simply replacing "can't" with "is not observed to" would be appropriate.

Response #5: As suggested, we have revised the text as **"***While about 20% of 5'$_{A36}$-xrRNA1 translocate through the nanopore channel with duration time longer than 100 s, the remaining 80% are still not observed to translocate through the pore even in a 300 s time window.*"

5) The MD section is entitled "Mechanical anisotropy of ZIKV xrRNA1 by MD simulations.". I think there is something missing (e.g. "probed by MD simulations"?)

Response #6: As suggested, we have modified the subtitle of the MD section as

"Mechanical anisotropy of ZIKV xrRNA1 probed by MD simulations".

6) In the MD section, there is the statement "To further support the hypothesis and gain insights into the mechanisms underlying ZIKV xrRNA1 mechanical anisotropy, we used steered MD...". It is not scientifically sound to claim that something is done in order to support the hypothesis. A hypothesis can be tested. However, this phrasing implies that the hypothesis was known to be true, and that simulations were simply performed in order to support it. If the simulations did not support the hypothesis, would the hypothesis or simulations be considered false? Replacing "further support the" with "further explore the" would be appropriate.

Response #7: As suggested, we have changed the phrase as *"To further explore the hypothesis and gain insights into the mechanisms underlying ZIKV xrRNA1 mechanical anisotropy, we used steered MD to investigate the mechanical unfolding of ZIKV xrRNA1".*

7) To finish with a comment, and not a criticism, the finding that the stress distribution in the structure-based SMOG model is centered around PK2 provides strong evidence that the architecture of the fold is responsible for the differential resistance to force, rather than energetic interactions. As a note, there are numerous typos in the methods and main text of this section, but they can be easily fixed. (e.g. "structure-base model" should be "structure-based model". "salvation" should be "solvation".)

Response #8: We thank the referee for pointing out the typos in our manuscript. We have carefully gone through the manuscript and corrected them.

The authors should also provide a full technical description in the SI that details the simulations with SMOG models(e.g. timestep, simulation duration, temperature, etc), such that can be reproduced by others.

Response #9: We feel sorry for the confusion about simulations with the SMOG

model. Actually, we performed force distribution analysis on the trajectories obtained with AMBER14 force field, which were once used for cross-correlation based contact analysis. To evaluate intramolecular stress of xrRNA1, we just leveraged the effective contact energy functions from SMOG instead of explicit solvent force field (i.e. AMBER14) and bypassed calculation for solvent shielding effect on electrostatic interaction among atoms, which is important to treat highly charged molecules like RNA. Prior to employing SMOG model for force distribution analysis, we have made a test to check whether structure-based model could capture essence of translocation-coupled unfolding for ZIKV xrRNA1. To clarify our description about force distribution analysis, we added information in Methods Section and provided an overview about the procedure of force distribution analysis which was presented as a flowchart in **Figure S18**. As the referee suggested, we added details of the simulations with SMOG model in the SI. Additionally, we also gave brief description about the simulation result with SMOG model, as well as comparison between simulations with SMOG and physical force field (i.e AMBER14).

Additionally, since the simulations with a SMOG model are quite similar to another recently reported set of simulations describing directional translocation ("Directional translocation resistance of Zika xrRNA" by Suma at el), there should be a brief description of the similarities and differences between the current simulations and the previously published results.

Response #10: As suggested, we added discussions in the main text and SI text to compare the similarities and differences between our results and the recent published work.

Thanks again for the comments and valuable suggestions to improve our manuscript.

Yours sincerely,

Xianyang Fang, Ph.D.
Assistant Professor
School of Life
Sciences
Tsinghua University
Beijing 100084, China
Office phone : 86-10-62771071
Cell phone: 86-18612937900
Email: fangxy@tsinghua.edu.cn

REVIEWERS' COMMENTS

Reviewer #2 (Remarks to the Author):

The authors have addressed all questions. The manuscript is a nice contribution that bridges our understanding of molecular structure and dynamics.

Only two minor changes to the methods. 1) The text says "smog-server ()" but the parentheses are empty. I'm guessing the authors intended to put the link to the smog-server at this point. 2) The primary reference for the Shadow Contact Map is not given (Noel et al, 2012, DOI:10.1021/jp300852d)

There is still a good number of misspellings scattered throughout the manuscript, but I believe this can be addressed during typesetting. No further review is requested.

The authors have addressed all questions. The manuscript is a nice contribution that bridges our understanding of molecular structure and dynamics.

Re: We thank again the reviewers whose comments have greatly improved this manuscript.

Only two minor changes to the methods.

1) The text says "smog-server ()" but the parentheses are empty. I'm guessing the authors intended to put the link to the smog-server at this point.

Re: We added the link in the parentheses.

2) The primary reference for the Shadow Contact Map is not given (Noel et al, 2012, DOI:10.1021/jp300852d)

Re: According to suggestion of referee, we added the reference for the Shadow Contact Map in our manuscript.

There is still a good number of misspellings scattered throughout the manuscript, but I believe this can be addressed during typesetting. No further review is requested.

Re: We have tried our best to fix the typo in our manuscript.
